# Efficient Amide Bond Formation through a Rapid and Strong Activation of Carboxylic Acids in a Microflow Reactor**

**DOI:** 10.1002/anie.201307987

**Published:** 2013-12-02

**Authors:** Shinichiro Fuse, Yuto Mifune, Takashi Takahashi

**Affiliations:** Department of Applied Chemistry, Tokyo Institute of Technology2-12-1, Ookayama, Meguro-ku, Tokyo 152-8552 (Japan); Faculty of Pharmaceutical Sciences, Yokohama College of Pharmacy601, Matana-cho, Totsuka-ku, Yokohama-shi 245-0066 (Japan)

**Keywords:** amides, amino acids, continuous flow, natural products, peptides

## Abstract

The development of highly efficient amide bond forming methods which are devoid of side reactions, including epimerization, is important, and such a method is described herein and is based on the concept of rapid and strong activation of carboxylic acids. Various carboxylic acids are rapidly (0.5 s) converted into highly active species, derived from the inexpensive and less-toxic solid triphosgene, and then rapidly (4.3 s) reacted with various amines to afford the desired peptides in high yields (74 %–quant.) without significant epimerization (≤3 %). Our process can be carried out at ambient temperature, and only CO_2_ and HCl salts of diisopropylethyl amine are generated. In the long history of peptide synthesis, a significant number of active coupling reagents have been abandoned because the highly active electrophilic species generated are usually susceptible to side reactions such as epimerization. The concept presented herein should renew interest in the use of these reagents.

Highly active species are attractive in organic synthesis because their reactivity allows rapid reaction of sterically and electronically less-reactive substrates. In contrast, these tend to cause undesired reactions and, therefore, suppression of the side reactions is key to their utilization. Recent dramatic progress in flash chemistry[1] has enabled the utilization of highly active species without side reactions by precisely controlling the reaction time (<1 s) and temperature by the utilization of microflow reactors.

Amide bonds are ubiquitous in nature, and are a key method of linkage for peptides and proteins.[2] The development of highly efficient methods for the formation of amide bonds without side reactions, including epimerization, is regarded as a highly important endeavor.[3] The use of highly electrophilic species such as acid chlorides and acid anhydrides has a long history in peptide synthesis,[4] as they are useful for the couplings of less nucleophilic amines such as *N*-methyl amino acids,[5] which have attracted great attention in recent years because of their contributions to the improvement of the metabolic stability of peptides.[2e] However, applications of acid chlorides and anhydrides are somewhat limited because their strong electrophilicity tends to cause side reactions. The most conventional approach is the condensation of amines with carboxylic acids by active esters using coupling reagents such as carbodiimides or phosphonium or uronium salts.[2] This method is based on a concept that is referred to as the mild activation of carboxylic acids, whereby epimerization is suppressed by the mild electrophilicity of the active species (Scheme 1). Thus, the reaction time is usually long and it is sometimes difficult to couple less-reactive substrates such as *N*-methyl amino acids.

**Scheme 1:**
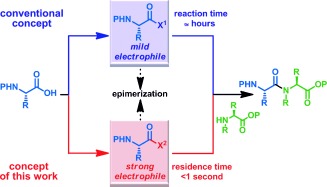
Amide bond formation based on a conventional concept of the mild activation of carboxylic acids and our concept of rapid and strong activation of carboxylic acids.

Herein, we report an efficient amide bond formation based on the concept of rapid and strong activation of carboxylic acids (Scheme 1). Various carboxylic acids, which included easily racemizable amino acids, were activated using an inexpensive and less-toxic solid triphosgene,[6] and the racemization was suppressed (≤3 %) by reducing the residence time of the highly active species[1] (0.5 s) through microflow synthesis.[7] The active species rapidly reacted (4.3 s) with less nucleophilic amines, including *N*-methyl amino acids, in good to excellent yields at ambient temperature (20 °C).

The microflow reactor that was used to test our concept is shown in Figure 1. We connected two T-shape mixers with Teflon® tubing and immersed them in a water bath (20 °C). A solution of carboxylic acid and base in solvent A was introduced into the first mixer with a syringe pump. The solution of triphosgene in solvent B was also introduced into the first mixer with a syringe pump. We speculated that the phosgene, generated in situ by the reaction between the base and triphosgene, converted the carboxylic acid into the corresponding acid chloride.[6,3n, w] We anticipated that the epimerization was suppressed by reducing the residence time of the highly electrophilic active species to less than one second. To accomplish amidation, a solution of the amine in solvent C was then introduced into the second mixer with a syringe pump. The reaction was quenched by pouring the mixture into a saturated aqueous solution of NH_4_Cl and CHCl_3_. In principle, this method only emits CO_2_ and the HCl salts of a base other than the substrate.

**Figure 1:**
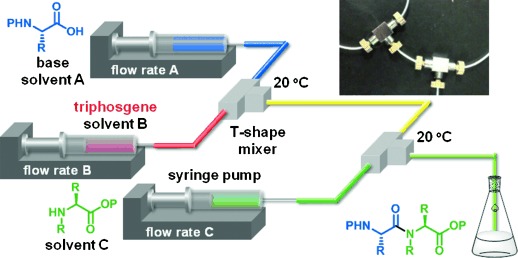
Microflow synthesis of peptides based on the concept of rapid and strong activation of carboxylic acids using triphosgene.

Initially, solvents were examined as shown in Table 1 (entries 1–10). *N*-Boc-*O*-benzyl-l-serine (**1**) and l-phenylalanine allyl ester (**2**) were employed as substrates since they have bulky side chains at the α-position. The serine **1** has an acid-labile Boc group on the amino group, and N-Boc serine is susceptible to epimerization. The combination of DMF for solvent A, and MeCN for solvents B and C afforded the best results (entry 7). Interestingly, the use of H_2_O as either solvent A or C afforded comparable results (entries 5, 8, and 10). Bases were examined in combination with an optimal solvent system (DMF/MeCN; Table 1, entries 7 and 11–17). As shown, DIEA afforded the best results (entry 7).

**Table 1:** Microflow amide bond formation with various solvents and bases.[a]
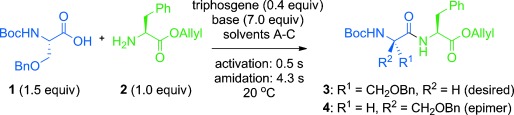

Entry	Solvent	Base	Yield [%]			
	A	B	C		3	4
1	CH_2_Cl_2_	CH_2_Cl_2_	CH_2_Cl_2_	DIEA	31	1
2	1,4-dioxane	1,4-dioxane	1,4-dioxane	DIEA	–[b]	–
3	MeCN	MeCN	MeCN	DIEA	48	2
4	*i*PrOH	MeCN	MeCN	DIEA	–[c]	–
5	MeCN	MeCN	MeCN/H_2_O (1:1)	DIEA	52	3
6	NMP	MeCN	MeCN	DIEA	<55	<10
7	DMF	MeCN	MeCN	DIEA	62	9
8	MeCN/H_2_O (9:1)	MeCN	MeCN	DIEA	56	15
9	MeCN/DMF (9:1)	MeCN	MeCN	DIEA	55	2
10	DMF/H_2_O (9:1)	MeCN	MeCN	DIEA	58	9
11	DMF	MeCN	MeCN	Et_3_N	–[b]	–
12	DMF	MeCN	MeCN	Me_2_NEt	52	10
13	DMF	MeCN	MeCN	Cy_2_NMe	63	15
14	DMF	MeCN	MeCN	lutidine	48	1
15	DMF	MeCN	MeCN	collidine	–[b]	–
16	DMF	MeCN	MeCN	DBU	24	9
17	DMF	MeCN	MeCN	DABCO	–[b]	–
18	H_2_O	MeCN	MeCN	LiOH	–[b]	–

[a] Flow rate A: 2000 μL min^−1^, flow rate B: 1200 μL min^−1^, flow rate C: 2000 μL min^−1^.

[b] Insoluble salts were generated.

[c] A complex mixture was obtained. Boc=*tert*-butoxycarbonyl, DABCO=1,4-diazabicyclo[2,2,2]octane, DBU=1,8-diazabicyclo[5.4.0]undec-7-ene, DIEA=*N*,*N*-diisopropylethylamine, DMF=*N*,*N*-dimethylformamide, NMP=*N*-methylpyrrolidone.

Quantities of **1** and DIEA were optimized (Table 2). The yield of the desired dipeptide **3** was improved with an increase in the amount of **1** (entries 1–3). In contrast, the yield of the epimer **4** decreased with a decreasing amount of DIEA (entries 3–5). The best results were obtained by employing 2.5 equivalents of **1**, and 3.0 equivalents of DIEA (entry 5). The residence time of the active species and reaction temperature were examined (see the Supporting Information). As a result, 0.5 seconds for the residence time and 20 °C for the reaction temperature proved to be the optimal combination.

**Table 2:** Optimization of quantities of carboxylic acid 1 and DIEA in microflow amide bond formation.[a[
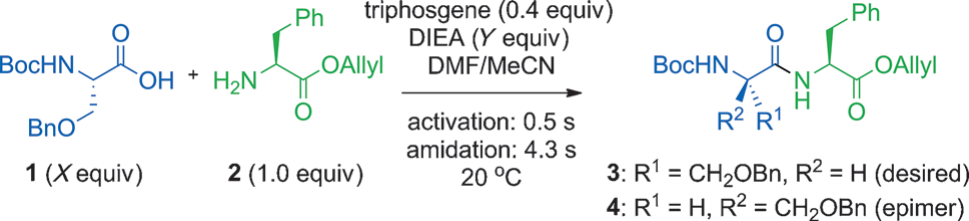

Entry	*X*	*Y*	Yield [%]	
			3	4
1	1.5	7.0	62	9
2	2.0	7.0	73	7
3	2.5	7.0	77	4
4	2.5	5.0	72	2
5	2.5	3.0	92	1

[a] Flow rate A: 2000 μL min^−1^, flow rate B: 1200 μL min^−1^, flow rate C: 2000 μL min^−1^.

The results of the scope and limitations of the established reaction conditions were examined and are shown in Table 3. The comparative batch reactions were carried out under the same reaction conditions as microflow reactions except for reaction times (activation: 30 s, amidation: 30 s). This modification was necessary because it was difficult to safely perform batch experiments (caution: large amounts of CO_2_ and phosgene evolved) and obtain reproducible results when shorter reaction times were used (<30 s). However, microflow experiments were performed safely, and reproducible results were obtained regardless of the short reaction times (activation: 0.5 s, amidation: 4.3 s). Although CO_2_ must be generated in the first T-shape mixer, neither bubbles nor gas plug flow was observed in the Teflon tubes. In the case of the coupling reactions between l-serine, l-tyrosine, or l-cysteine, as carboxylic acids, and l-phenylalanine as an amine, severe epimerization did not occur even when batch conditions were used (entries 1, 2, and 4). However, a reduction in the yield was observed under batch conditions probably because of the acidic removal of Boc or triphenylmethyl (Trt) groups. The desired dipeptides **3**, **5**, and **7** were, however, obtained in excellent yields (92 %, quant., and 94 %, respectively) under the microflow conditions (entries 1, 2, and 4). In the case of easily racemizable l-histidine, the generation of 17 % of the epimer was observed under batch conditions, but microflow conditions suppressed the epimerization to 2 % (entry 3). To our delight, highly racemizable D-phenylglycine was successfully coupled with l-phenylalanine in an excellent yield (97 %) accompanied by only 3 % of epimer (entry 5) under microflow conditions. In this case, it was necessary to perform the reaction at 10 °C using MeCN as solvent A instead of DMF. A decreased yield (74 %) with a substantial generation of the epimer (18 %) was observed by employing the batch conditions (entry 5). Less-nucleophilic bulky amines such as l-isoleucine, l-proline, and sarcosine were coupled with l-serine in good yields (89, 80, and 74 %, respectively) without detectable epimerization (entries 6–8). The corresponding batch reactions resulted in a reduction in yields (47, 58 and 53 %, respectively). Unexpectedly, l-lactic acid was coupled with l-phenylalanine in an excellent yield (98 %) without detectable epimerization, although a secondary alcohol in the lactic acid remained unprotected (entry 9). A severe reduction in yield (<28 %) was observed when the same reaction was performed under batch conditions (entry 9). It should be noted that although an excess amount (2.5–3.0 equiv) of carboxylic acid was employed under our established reaction conditions, unreacted carboxylic acid could be readily recovered using a simple acid-base extraction and phase-separation techniques during the workup process. In the case of the microflow synthesis of the dipeptide **3**, 74 % of unreacted **1** was recovered without detectable epimerization (<1 %). This proved the feasibility of our concept of a rapid and strong activation of carboxylic acids for highly efficient amide bond formation.

**Table 3:** Microflow amide bond formation with various substrates. 
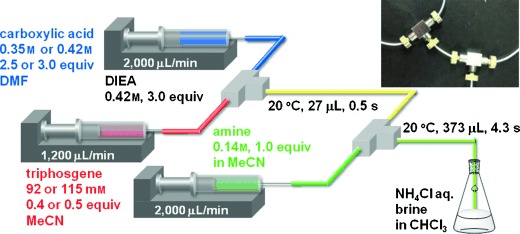

Entry	Structure of	Cond.	Yield [%]	
	desired product		(desired)	(epimer)
1	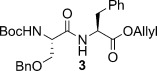	flow A[a] batch[d]	92 57	1 2
2	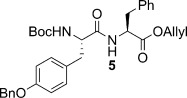	flow B[b] batch[d]	quant. 40	<1 <1
3	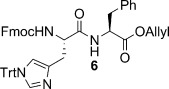	flow B[b] batch[d]	92 75	2 17
4	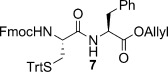	flow A[a] batch[d]	94 71	<1 1
5	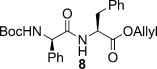	flow C[c] batch[d]	97 74	3 18
6	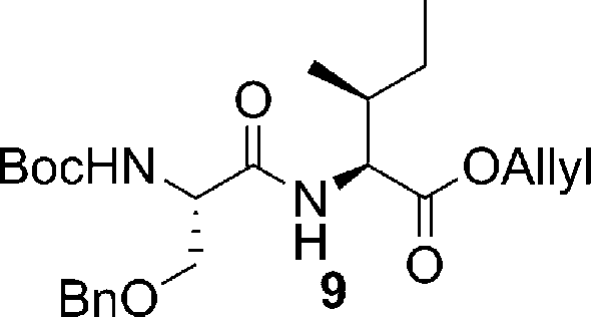	flow B[b] batch[d]	89 47	<1 1
7	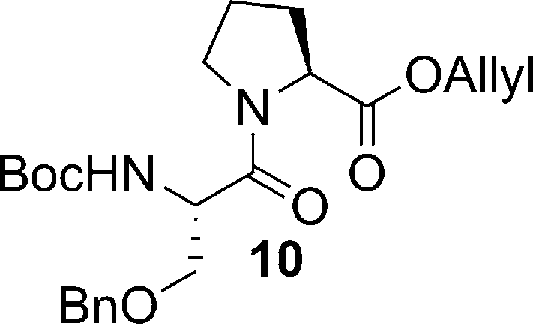	flow A[a] batch[d]	80 58	<1 1
8	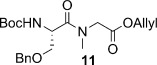	flow A[a] batch[d]	74 53	<1 <1
9	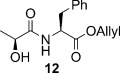	flow A[a] batch[d]	98 <28	<1

[a] Carboxylic acid: 2.5 equiv, triphosgene: 0.4 equiv.

[b] Carboxylic acid: 3.0 equiv, triphosgene: 0.5 equiv.

[c] Carboxylic acid: 2.5 equiv, triphosgene: 0.4 equiv, DIEA: 2.5 equiv, solvent A: MeCN, reaction temperature: 10 °C.

[d] Reaction time for the activation of carboxylic acid and the amidation: 30 s. Fmoc=9-fluorenylmethyloxycarbonyl.

Next, we turned our attention to the identification of the actual active species. We utilized an in situ IR detection system (Mettler-Toledo React IR 15 Microflow cell) to identify the active species. A DMF solution of an l-valine derivative, DIEA, and a MeCN solution of triphosgene were introduced to the T-shape mixer, and the reaction mixture, after 0.5 seconds of the mixing of the two solutions, was analyzed by the in situ IR detection system (see the Supporting Information). Unexpectedly, comparisons between the observed IR spectra and separately prepared reference compounds indicated that the symmetric anhydride was the actual active species.[8] This result means that a highly electrophilic anhydride was generated quantitatively in only 0.5 seconds, although the detailed pathway was unclear.

Finally, we tried to synthesize a tetrapeptide moiety of the depsipeptidic natural product auliride (**13**)[9] to extend the range of applications for our established process. Tetrapeptide contains two *N*-methyl amino acids (Scheme 2). The esterification of **14** was followed by the removal of the Fmoc group in the batch reactor because a long reaction time was required, and careful control of the reaction conditions unnecessary. Removal of the Fmoc group meant that the CH_2_Cl_2_ solvent and Et_2_NH were also removed after the completion of the reaction, and that the residue was dissolved in MeCN. The resultant solution was removed by syringe and was used for microflow amidation with *N*-Fmoc sarcosine. The desired dipeptide **15** was obtained in an excellent yield (2 steps 87 %). In this reaction, 2.6 grams of **15** were obtained by continuous running of the microflow reactor for 24 minutes. Thus, the productivity of the established process was satisfactory. To our delight, the subsequent coupling of *N*-Fmoc-*N*-methyl-D-leucine with the less-nucleophilic dipeptide **15** afforded the desired tripeptide **16** in a good yield (2 steps 83 %) without detectable epimerization. The final coupling of the tripeptide **16** with *N*-Fmoc-l-valine was somewhat slow and therefore, the crude reaction mixture obtained from the microflow reactor was collected in a dried batch reactor and stirred for 10 minutes before the reaction was quenched. The desired tetrapeptide **17** was obtained in a good yield (2 steps 60 %) without detectable epimerization. Moreover, our developed process could be used for the coupling of dipeptides (see the Supporting Information). The desired tetrapeptide **17** was obtained in a satisfactory yield without detectable epimerization.

**Scheme 2:**
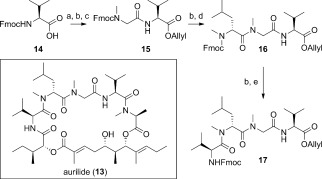
Synthesis of the tetrapeptide 17, a substructure of aurilide (13). a) Batch, AllylBr, K_2_CO_3_, DMF, RT, 19 h, 93 %. b) Batch, Et_2_NH, CH_2_Cl_2_, RT, 2.5 h. c) Flow A, *N*-Fmoc-sarcosine, 2 steps 87 %. d) Flow A, *N*-Fmoc-*N*-methyl-D-leucine, 2 steps 83 %, epimer <1 %. e) Flow and batch, *N*-Fmoc-l-valine, 2 steps 60 %, epimer <1 %.

In summary, we demonstrated highly efficient amide bond formation based on our concept of rapid and strong activation of carboxylic acids. The various carboxylic acids were rapidly (0.5 s) converted into highly active species using inexpensive, less-toxic solid triphosgene, and they were rapidly (4.3 s) reacted with various amines to afford the desired peptides in high yields (74 %–quant.) without severe epimerization (≤3 %). Moreover, our developed process was applicable to the synthesis of a tetrapeptide moiety in the depsipeptidic natural product aurilide. Our process was carried out at an ambient temperature (20 °C), and it generated only CO_2_ and HCl salts of DIEA, with the exception of recovered carboxylic acid. These excellent results could not be achieved under the conventional batch conditions because the precise control of temperature and time is difficult. In the long history of peptide synthesis, a significant number of active coupling reagents have been abandoned because the highly electrophilic active species generated by the active reagents were usually highly susceptible to side reactions such as epimerization.[2c] Our concept should prompt a renewal of interest in the use of these reagents. The wise use of microflow synthesis should dramatically increase the value of highly active reagents.
