# Supplementary material for: Efficient Amide Bond Formation through a Rapid and Strong Activation of Carboxylic Acids in a Microflow Reactor
Source: Angew Chem Int Ed Engl. 2013 Dec 2;53(3):851–5. doi: 10.1002/anie.201307987 (PMC4499250; doi:10.1002/anie.201307987)

Angewandte Chemie
Eine Zeitschrift der Gesellschaft Deutscher Chemiker

Supporting Information

© Wiley-VCH 2013

69451 Weinheim, Germany

Efficient Amide Bond Formation through a Rapid and Strong Activation of Carboxylic Acids in a Microflow Reactor**

Shinichiro Fuse, Yuto Mifune, and Takashi Takahashi*

anie_201307987_sm_miscellaneous_information.pdf

Supporting Information

Table of Contents

1. General techniques S-2
2. Micro-flow reactor setup S-2
3. General procedure for amide bond formation with various solvents and bases using micro-flow reactor S-4
4. General procedure for optimization of quantities of carboxylic acid **1** and DIEA in micro-flow amide bond formation S-6
5. General procedure for optimization of the residence time of the active species and reaction temperature in micro-flow amide bond formation S-7
6. Procedure for recovery of unreacted carboxylic acid **1** S-9
7. General procedures for amide bond formation with various substrates
  7.1. flow A and flow B S-10
  7.2. flow C S-11
  7.3. batch S-11
8. Preparation of epimers S-16
9. In situ IR analysis
  9.1. General procedure for in situ IR analysis S-21
  9.2. Synthesis of reference compounds S-21
  9.3. IR spectra S-22
10. Synthesis of tetrapeptide moiety of aurilide (**13**)
  10.1. Linear route S-26
  10.2. Convergent route S-30
  10.3. Synthesis of epimers S-34
11. HPLC charts S-40
12. NMR spectra S-47

1. General techniques

NMR spectra were recorded on a JEOL Model ECP-400 (400 MHz for ^{1}H, 100 MHz for ^{13}C) instrument in the indicated solvent. Chemical shifts are reported in units of parts per million (ppm) relative to the signal (0.00 ppm) for internal tetramethylsilane for solutions in $CDCl_3$ (7.26 ppm for ^{1}H, 77.0 ppm for ^{13}C). Multiplicities are reported by using the following abbreviations: s; singlet, d; doublet, t; triplet, q; quartet, m; multiplet, br; broad, *J*; coupling constants in Hertz (Hz). IR spectra were recorded on a Perkin-Elmer Spectrum One FT-IR spectrometer. Only the strongest and/or structurally important peaks are reported as the IR data given in cm^{-1}. Optical rotations were measured with JASCO model P-1020 polarimeter. UV absorptions were measured with a Perkin-Elmer Lambda 40 UV/VIS spectrometer. HRMS (ESI-TOF) were measured with a Waters LCT Premier™ XE.

Synthesis of epimers at chapter 8 were carried out by using parallel synthesizer, Zodiac® CCX-1101 which was purchased from Tokyo Rikakikai (EYELA) co. ltd., Japan. Zodiac has 12 glass vessels (1-15 mL scale).

All reactions were monitored by thin-layer chromatography carried out on 0.25 mm E. Merck silica gel plates (60F-254) with UV light, visualized by 10% ethanolic phosphomolybdic acid or 0.5% nynhydrin *n*-butanol solution. Flash column chromatography was performed on Silica Gel 60 N, purchased from Kanto Chemical Co. Analytical HPLC was carried out on a Waters 2695 Separations Module with a Waters 2996 Photodiode Array Detector and Waters 2414 Refractive Index Detector. Gel permeation chromatography (GPC) for qualitative analysis were performed on Japan Analytical Industry Model LC908 (recycling preparative HPLC), on a Japan Analytical Industry Model RI-5 refractive index detector and on a Japan Analytical Model 310 ultra violet detector with a polystyrene gel column (JAIGEL-1H, 20 mm×600 mm), using chloroform as solvent (3.5 mL/min). DIEA was distilled from ninhydrine and KOH.

2. Micro-flow reactor setup

Stainless steel T-shape mixers were purchased from Sanko Seiki Co. Ltd. (inner diameter: 0.25 mm). Teflon® tube with inner diameter 0.8, 0.5 and 0.25 mm was purchased from Senshu Scientific Co. Ltd. The T-shape mixers and Teflon® tube were connected with PEEK fittings purchased from GL Science Co. Ltd. Solutions were introduced to micro-flow system with the syringe pumps (Harvard Pump 11 and Harvard PHD ULTRA) equipped gastight syringes (SGE). The gastight syringes and Teflon® tube were connected with joints purchased from Techno Applications Co. Ltd. In situ IR analysis was performed using Mettler-Toledo React IR™ 15 Micro-flow cell.

The employed micro-flow system is shown in Figure S-1. The gastight syringes and T-shape mixers 1 and 2 were connected with Teflon® tube (inner diameter: 0.8 mm, length: 800 mm, volume 402 μL). T-shape mixers 1 and 2 were connected with reaction tube 1 (Teflon® tube). T-shape mixer 2 was connected with reaction tube 2 (Teflon® tube). The reaction tube 2 and Teflon® tube (inner diameter: 0.25 mm, length 100 mm, volume 5 μL) were connected to generate back pressure. T-shape mixers and reaction tube were immersed in water bath.

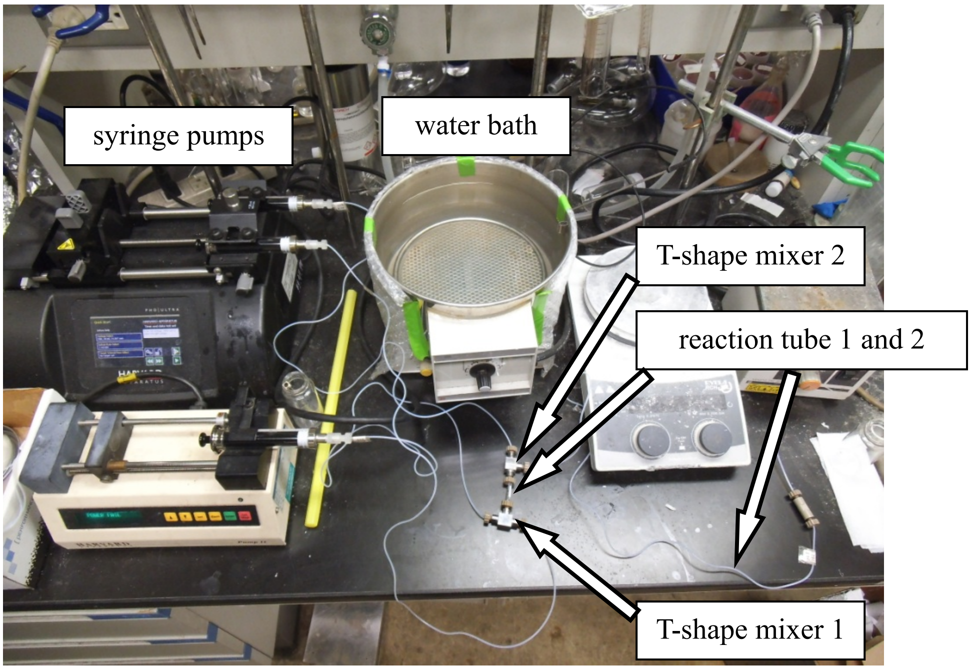

Figure S-1

The micro-flow system for in situ IR analysis is shown Figure S-2. The gastight syringes and T-shape mixer 1 were connected with Teflon® tube (inner diameter: 0.8 mm, length: 800 mm, volume 402 μL). T-shape mixer 1 and ReactIR™ 15 Micro Flow Cell were connected with reaction tube 1 (Teflon® tube).

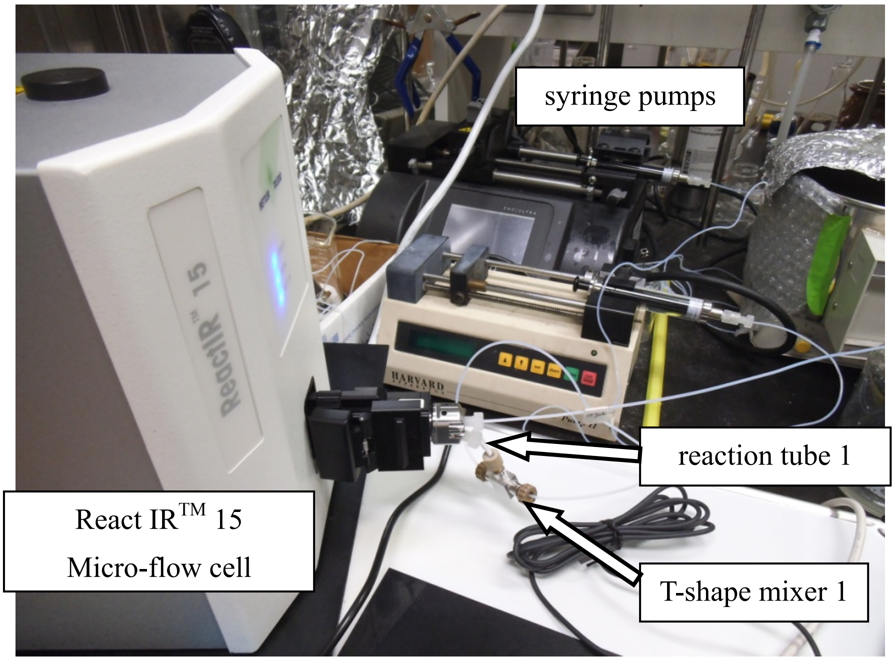

Figure S-2

3. General procedure for amide bond formation with various solvents and bases using micro-flow reactor

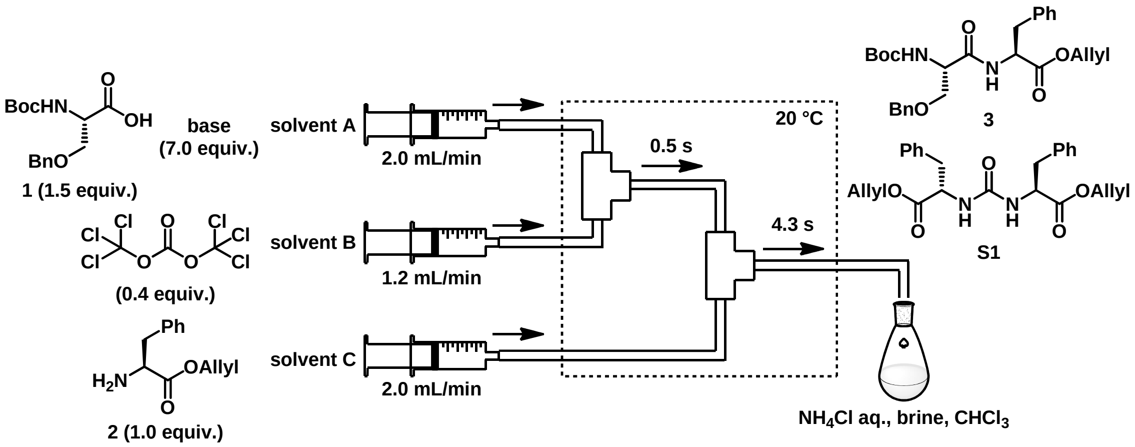

A solution of Boc-L-Ser(*O*-Bn)-OH (**1**) (0.21 M, 1.5 equiv.) and **base** (0.98 M, 7.0 equiv.) in **solvent A** (flow rate: 2.0 mL/min) and a solution of triphosgene (0.092 M, 0.4 equiv.) in **solvent B** (flow rate: 1.2 mL/min) were introduced to T-shape mixer 1 at 20 °C with the syringe pumps. The resultant mixture was passed through reaction tube 1 (inner diameter: 0.8 mm, length: 54 mm, volume: 27 μL, reaction time: 0.5 s) at the same temperature. Then, the resultant mixture and a solution of H-L-Phe-OAllyl (**2**) (0.14 M, 1.0 equiv.) in **solvent C** (flow rate: 2.0 mL/min) were introduced to T-shape mixer 2 at 20 °C. The resultant mixture was passed through reaction tube 2 (inner diameter: 0.8 mm, length: 742 mm, volume: 373 μL, reaction time: 4.3 s) at the

same temperature. After being eluted for 30-40 s to reach a steady state, the resultant mixture was poured into saturated aqueous NH_4Cl, brine and $CHCl_3$ suspension for 30 s at room temperature. The aqueous layer was extracted twice with $CHCl_3$. The combined organic layer was washed with 1 M HCl, saturated aqueous $NaHCO_3$ and brine, dried over $MgSO_4$, filtered and concentrated in vacuo. The residue was purified by column chromatography on silica gel (25% EtOAc in hexane) to give a mixture of Boc-L-Ser(*O*-Bn)-L-Phe-OAllyl (**3**), epimer **4** and (*S*,*S*)-*N*,*N′*-bis(1-allyloxycarbonyl-2-phenylethyl)urea (**S1**). Yield was determined by HPLC-UV analysis (conditions: DAICEL CHIRALPAK ID 4.6 mm×25 cm, 15% EtOH in hexane, flow rate 1 mL/min, detection wavelength 259 nm).

Boc-L-Ser(*O*-Bn)-L-Phe-OAllyl (3)

Retention time 11.48 min (epimer 14.50 min)

White solid; ^{1}H NMR (400 MHz, $CDCl_3$): δ 7.34-7.26 (m, 5H), 7.19-7.17 (m, 3H), 7.06 (m, 3H), 5.83 (m, 1H), 5.35 (br, 1H), 5.30-5.22 (m, 2H), 4.86 (ddd, *J* = 5.8, 6.4, 5.4 Hz, 1H), 4.58-4.45 (m, 4H), 4.28 (br, 1H), 3.90 (brd, *J* = 6.8 Hz, 1H), 3.54 (dd, *J* = 6.8, 8.8 Hz, 1H), 3.14 (dd, *J* = 5.4, 13.7 Hz, 1H), 3.06 (dd, *J* = 5.8, 13.7 Hz, 1H), 1.42 (s, 9H); ^{13}C NMR (100 MHz, $CDCl_3$): δ 170.6, 170.0, 155.3, 137.3, 135.6, 131.3, 129.3, 128.4, 127.8, 127.8, 127.0, 118.9, 80.2, 73.4, 69.8, 65.9, 53.7, 53.4, 37.7, 28.2; IR (neat): 3326, 2978, 1715, 1676, 1496, 1367, 1168, 772, 743, 700 cm^{-1}; $[\alpha]^{26}_D$ = +31.8 (c 1.06, $CHCl_3$); mp 87-89 °C; UV ($CHCl_3$): λ_{max} = 259 nm (ε = 464 L mol^{-1} cm^{-1}); HRMS (ESI-TOF): calcd for $[C_{27}H_{34}N_2O_6 + H]^+$ 483.2495, found 483.2490.

(*S*,*S*)-*N*,*N′*-bis(1-allyloxycarbonyl-2-phenylethyl)urea (S1)

Retention time 8.78 min

White solid; ^{1}H NMR (400 MHz, $CDCl_3$): δ 7.24-7.08 (m, 6H), 7.07 (d, *J* = 5.9 Hz, 4H), 5.82 (m, 2H), 5.42 (d, *J* = 7.8 Hz, 2H), 5.29-5.21 (m, 4H), 4.81 (m, 2H), 4.49 (m, 4H), 2.99 (brd, *J* = 4.3 Hz, 4H); ^{13}C NMR (100 MHz, $CDCl_3$): δ 172.5, 156.2, 136.0, 131.4, 129.4, 128.4, 126.9, 118.8, 65.9, 53.9, 38.6; IR (neat): 3358, 1731, 1635, 1564, 1558, 1190, 699 cm^{-1}; $[\alpha]^{24}_D$ = +52.0 (c 0.96, $CHCl_3$); mp 148-150 °C; UV ($CHCl_3$): λ_{max} = 259 nm (ε = 337 L mol^{-1} cm^{-1}); HRMS (ESI-TOF): calcd for $[C_{25}H_{28}N_2O_5 + H]^+$ 437.2076, found 437.2076.

Table S-1

entry	solvent A	solvent B	solvent C	base	yield **3** (%)	yield **4** (%)	yield **S1** (%)
1	CH_2Cl_2	CH_2Cl_2	CH_2Cl_2	DIEA	31	1	62
2	dioxane	dioxane	dioxane	DIEA	-[a]	-	-
3	MeCN	MeCN	MeCN	DIEA	48	2	30

4	*i*-PrOH	MeCN	MeCN	DIEA	-[b]	-	-
5	MeCN	MeCN	MeCN/H_2O (1/1)	DIEA	52	3	15
6	NMP	MeCN	MeCN	DIEA	<55	<10	<2
7	DMF	MeCN	MeCN	DIEA	62	9	2
8	MeCN/H_2O (9/1)	MeCN	MeCN	DIEA	56	15	19
9	MeCN/DMF (9/1)	MeCN	MeCN	DIEA	55	2	23
10	DMF/H_2O (9/1)	MeCN	MeCN	DIEA	58	9	2
11	DMF	MeCN	MeCN	Et_3N	-[a]	-	-
12	DMF	MeCN	MeCN	Me_2NEt	52	10	2
13	DMF	MeCN	MeCN	Cy_2NMe	63	15	2
14	DMF	MeCN	MeCN	lutidine	48	1	0
15	DMF	MeCN	MeCN	collidine	-[a]	-	-
16	DMF	MeCN	MeCN	DBU	24	9	8
17	DMF	MeCN	MeCN	DABCO	-[a]	-	-
18	H_2O	MeCN	MeCN	LiOH	-[a]	-	-

[a] Insoluble salts were generated. [b] A complex mixture was obtained.

4. General procedure for optimization of quantities of carboxylic acid 1 and DIEA in micro-flow amide bond formation

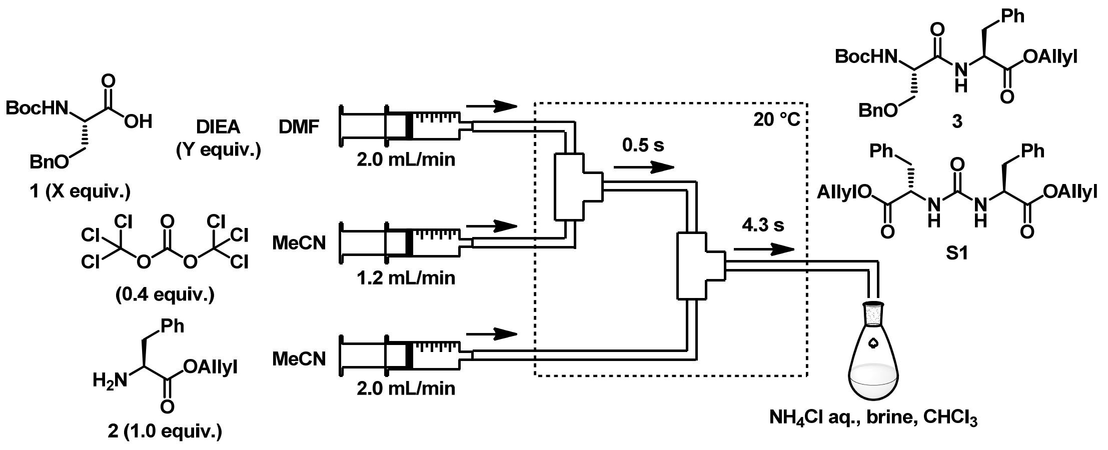

A solution of Boc-L-Ser(*O*-Bn)-OH (**1**) (**X equiv.**) and DIEA (**Y equiv.**) in DMF (flow rate: 2.0 mL/min) and a solution of triphosgene (0.092 M, 0.4 equiv.) in MeCN (flow rate: 1.2 mL/min) were introduced to T-shape mixer 1 at 20 °C with the syringe pumps. The resultant

mixture was passed through reaction tube 1 (inner diameter: 0.8 mm, length: 54 mm, volume: 27 μL, reaction time: 0.5 s) at the same temperature. Then, the resultant mixture and a solution of H-L-Phe-OAllyl (**2**) (0.14 M, 1.0 equiv.) in MeCN (flow rate: 2.0 mL/min) were introduced to T-shape mixer 2 at 20 °C. The resultant mixture was passed through reaction tube 2 (inner diameter: 0.8 mm, length: 742 mm, volume: 373 μL, reaction time: 4.3 s) at the same temperature. After being eluted for 30-40 s to reach a steady state, the resultant mixture was poured into saturated aqueous NH_4Cl, brine and $CHCl_3$ suspension for 30 s at room temperature. The aqueous layer was extracted twice with $CHCl_3$. The combined organic layer was washed with 1 M HCl, saturated aqueous $NaHCO_3$ and brine, dried over $MgSO_4$, filtered and concentrated in vacuo. The residue was purified by column chromatography on silica gel (25% EtOAc in hexane) to give a mixture of Boc-L-Ser(*O*-Bn)-L-Phe-OAllyl (**3**), epimer **4** and (*S*,*S*)-*N*,*N'*-bis(1-allyloxycarbonyl-2-phenylethyl)urea (**S1**). Yield was determined by HPLC-UV analysis (conditions: DAICEL CHIRALPAK ID 4.6 mm×25 cm, 15% EtOH in hexane, flow rate 1 mL/min, detection wavelength 259 nm).

Table S-2

entry	X (equiv.)	Y (equiv.)	yield **3** (%)	yield **4** (%)	yield **S1** (%)
1	1.5	7.0	62	9	2
2	2.0	7.0	73	7	2
3	2.5	7.0	77	4	0
4	2.5	5.0	72	2	0
5	2.5	3.0	92	1	0

5. General procedure for optimization of the residence time of the active species and reaction temperature in micro-flow amide bond formation

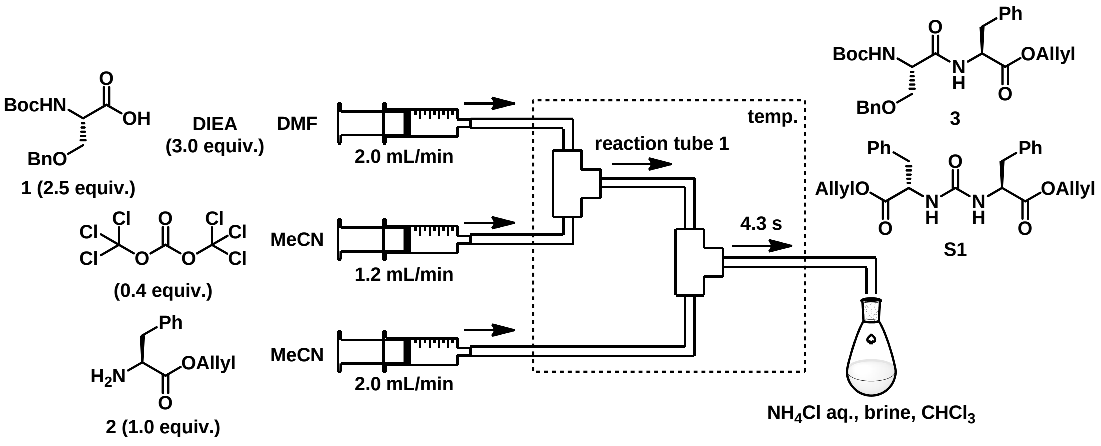

A solution of Boc-L-Ser(*O*-Bn)-OH (**1**) (0.35 M, 2.5 equiv.) and DIEA (0.42 M, 3.0 equiv.) in DMF (flow rate: 2.0 mL/min) and a solution of triphosgene (0.092 M, 0.4 equiv.) in MeCN (flow rate: 1.2 mL/min) were introduced to T-shape mixer 1 at **temp.** with the syringe pumps. The resultant mixture was passed through **reaction tube 1** at the same temperature. Then, the resultant mixture and a solution of H-L-Phe-OAllyl (**2**) (0.14 M, 1.0 equiv.) in MeCN (flow rate: 2.0 mL/min) were introduced to T-shape mixer 2 at **temp.** The resultant mixture was passed through reaction tube 2 (inner diameter: 0.8 mm, length: 742 mm, volume: 373 μL, reaction time: 4.3 s) at the same temperature. After being eluted for 30-40 s to reach a steady state, the resultant mixture was poured into saturated aqueous NH_4Cl, brine and $CHCl_3$ suspension for 30 s at room temperature. The aqueous layer was extracted twice with $CHCl_3$. The combined organic layer was washed with 1 M HCl, saturated aqueous $NaHCO_3$ and brine, dried over $MgSO_4$, filtered and concentrated in vacuo. The residue was purified by column chromatography on silica gel (25% EtOAc in hexane) to give a mixture of Boc-L-Ser(*O*-Bn)-L-Phe-OAllyl (**3**), epimer **4** and (*S*,*S*)-*N*,*N'*-bis(1-allyloxycarbonyl-2-phenylethyl)urea (**S1**). Yield was determined by HPLC-UV analysis (conditions: DAICEL CHIRALPAK ID 4.6 mm×25 cm, 15% EtOH in hexane, flow rate 1 mL/min, detection wavelength 259 nm).

Table S-3

entry	reaction tube 1	temp. (°C)	yield **3** (%)	yield **4** (%)	yield **S1** (%)
1	inner diameter: 0.5 mm, length: 41 mm, volume: 8 μL, reaction time: 0.15 s	20	66	0	4
2		30	77	1	0
3		40	78	1	0
4		50	83	2	0
5	inner diameter: 0.8 mm, length: 54 mm, volume: 27 μL, reaction time: 0.5 s	20	92	1	0
6		30	86	1	0
7		40	84	1	0
8		50	80	2	0
9	inner diameter: 0.8 mm, length: 159 mm, volume: 80 μL, reaction time: 1.5 s	20	93	1	0
10		30	78	1	0
11		40	77	1	0
12		50	69	2	0

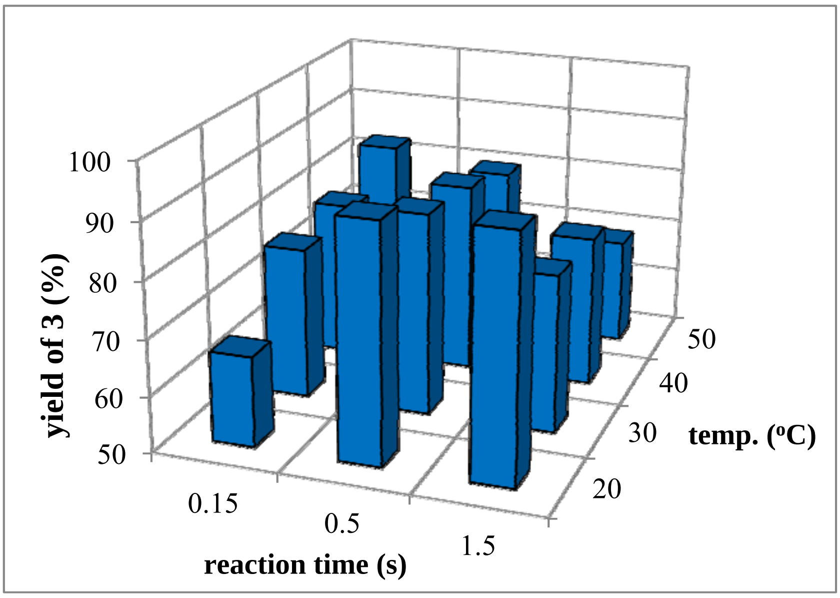

Figure S-3

6. Procedure for recovery of unreacted carboxylic acid 1

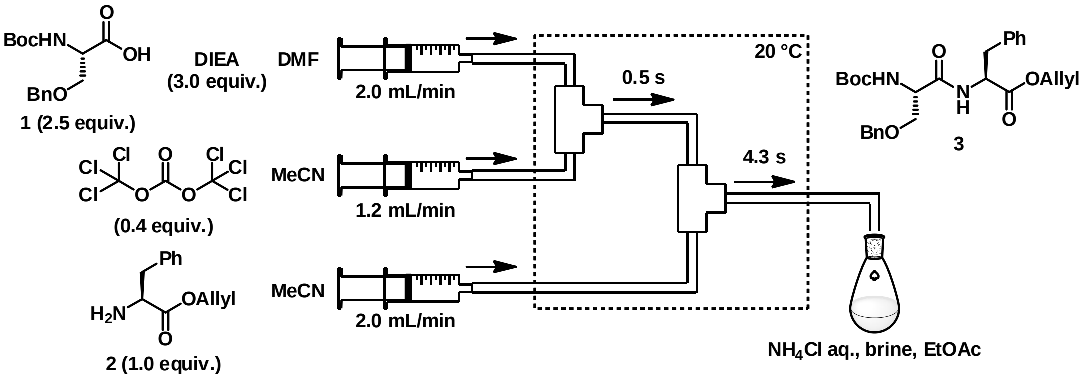

A solution of Boc-L-Ser(*O*-Bn)-OH (**1**) (0.35 M, 2.5 equiv.) and DIEA (0.42 M, 3.0 equiv.) in DMF (flow rate: 2.0 mL/min) and a solution of triphosgene (0.092 M, 0.4 equiv.) in MeCN (flow rate: 1.2 mL/min) were introduced to T-shape mixer 1 at 20 °C with the syringe pumps. The resultant mixture was passed through reaction tube 1 (inner diameter: 0.8 mm, length: 54 mm, volume: 27 μL, reaction time: 0.5 s) at the same temperature. Then, the resultant mixture and a solution of H-L-Phe-OAllyl (**2**) (0.14 M, 1.0 equiv.) in MeCN (flow rate: 2.0 mL/min) were introduced to T-shape mixer 2 at 20 °C. The resultant mixture was passed through reaction tube 2 (inner diameter: 0.8 mm, length: 742 mm, volume: 373 μL, reaction time: 4.3 s) at the same temperature. After being eluted for 40 s to reach a steady state, the resultant mixture was poured into saturated aqueous NH_4Cl, brine and EtOAc suspension for 30 s at room temperature. The aqueous layer was extracted twice with EtOAc. The combined organic layer was washed with 1 M HCl, saturated aqueous $NaHCO_3$ and brine, dried over $MgSO_4$, filtered and

concentrated in vacuo. The residue was purified by column chromatography on silica gel (25% EtOAc in hexane) to give Boc-L-Ser(*O*-Bn)-L-Phe-OAllyl (**3**) (58 mg, 0.120 mmol, 85%, epimer **4** <1%). Yield was determined by HPLC-UV analysis (conditions: DAICEL CHIRALPAK ID 4.6 mm×25 cm, 15% EtOH in hexane, flow rate 1 mL/min, detection wavelength 259 nm). The combined aqueous layer was acidified with 3 M HCl at 0 °C and extracted with EtOAc. The organic layer was washed with brine, dried over $MgSO_4$, filtered and concentrated in vacuo to give Boc-L-Ser(*O*-Bn)-OH (**1**) (50 mg, 0.169 mmol, ee = >99%, NMR spectra were shown at chapter 12) as a colorless oil. Enantiomeric excess was determined by HPLC-UV analysis (conditions: DAICEL CHIRALPAK ID 4.6 mm×25 cm, 0.1% TFA and 7% IPA in hexane, flow rate 1 mL/min, detection wavelength 258 nm, retention time 11.40 min, epimer 12.73 min).

7. General procedures for amide bond formation with various substrates

7.1. flow A and flow B

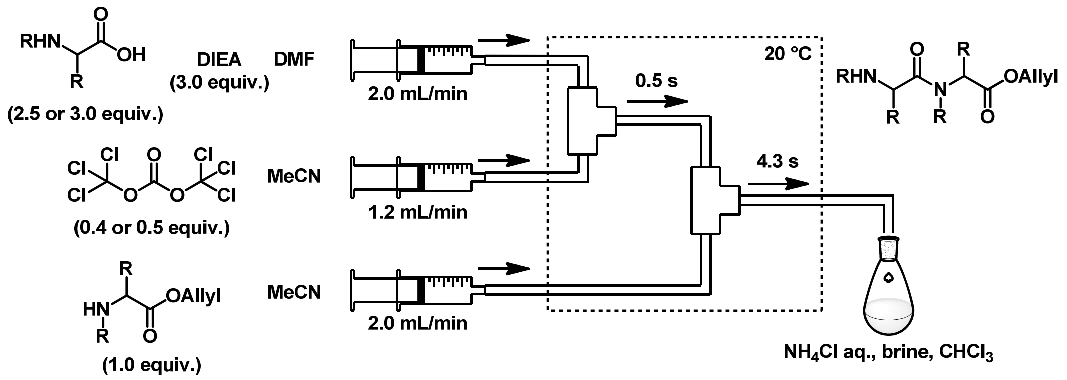

A solution of **carboxylic acid** (0.35 M, 2.5 equiv. for flow A or 0.42 M, 3.0 equiv. for flow B) and DIEA (0.42 M, 3.0 equiv.) in DMF (flow rate: 2.0 mL/min) and a solution of triphosgene (0.092 M, 0.4 equiv. for flow A or 0.117 M, 0.5 equiv. for flow B) in MeCN (flow rate: 1.2 mL/min) were introduced to T-shape mixer 1 at 20 °C with the syringe pumps. The resultant mixture was passed through reaction tube 1 (inner diameter: 0.8 mm, length: 54 mm, volume: 27 μL, reaction time: 0.5 s) at the same temperature. Then, the resultant mixture and a solution of **amine** (0.14 M, 1.0 equiv.) in MeCN (flow rate: 2.0 mL/min) were introduced to T- shape mixer 2 at 20 °C. The resultant mixture was passed through reaction tube 2 (inner diameter: 0.8 mm, length: 742 mm, volume: 373 μL, reaction time: 4.3 s) at the same temperature. After being eluted for 40 s to reach a steady state, the resultant mixture was poured into saturated aqueous NH_4Cl, brine and $CHCl_3$ suspension for 30-60 s at room temperature. The aqueous layer was extracted twice with $CHCl_3$. The combined organic layer was washed with 1 M HCl, saturated aqueous $NaHCO_3$ and brine, dried over $MgSO_4$, filtered and concentrated in vacuo. The residue was purified by column chromatography on silica gel to give a mixture of **desired product** and

epimer. Yield was determined by HPLC-UV analysis.

7.2. flow C

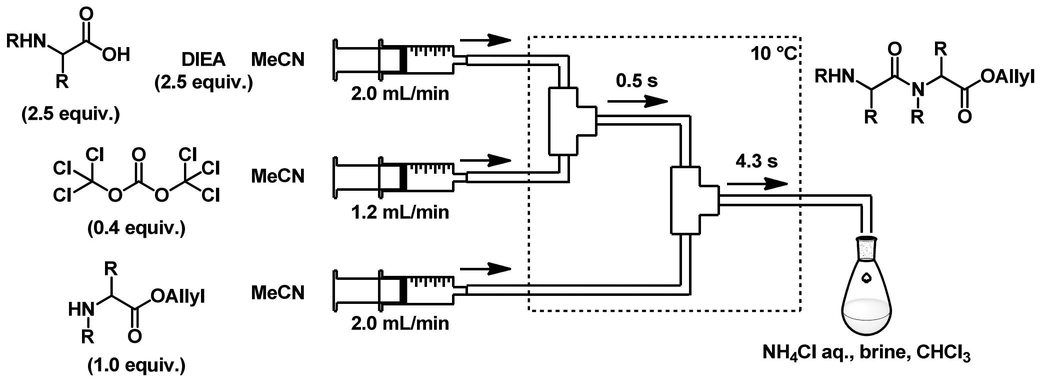

A solution of **carboxylic acid** (0.35 M, 2.5 equiv.) and DIEA (0.35 M, 2.5 equiv.) in MeCN (flow rate: 2.0 mL/min) and a solution of triphosgene (0.092 M, 0.4 equiv.) in MeCN (flow rate: 1.2 mL/min) were introduced to T-shape mixer 1 at 10 °C with the syringe pumps. The resultant mixture was passed through reaction tube 1 (inner diameter: 0.8 mm, length: 54 mm, volume: 27 μL, reaction time: 0.5 s) at the same temperature. Then, the resultant mixture and a solution of **amine** (0.14 M, 1.0 equiv.) in MeCN (flow rate: 2.0 mL/min) were introduced to T- shape mixer 2 at 10 °C. The resultant mixture was passed through reaction tube 2 (inner diameter: 0.8 mm, length: 742 mm, volume: 373 μL, reaction time: 4.3 s) at the same temperature. After being eluted for 40 s to reach a steady state, the resultant mixture was poured into saturated aqueous NH_4Cl, brine and $CHCl_3$ suspension for 30 s at room temperature. The aqueous layer was extracted twice with $CHCl_3$. The combined organic layer was washed with 1 M HCl, saturated aqueous $NaHCO_3$ and brine, dried over $MgSO_4$, filtered and concentrated in vacuo. The residue was purified by column chromatography on silica gel to give a mixture of **desired product** and **epimer**. Yield was determined by HPLC-UV analysis.

7.3. batch

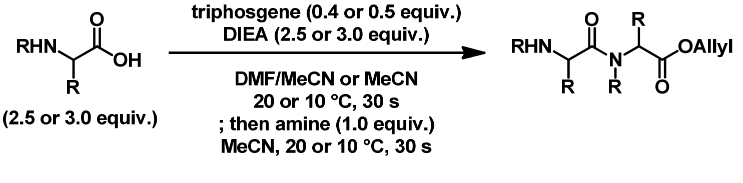

(*Quantities of compounds, solvents and temperature were identical to those of flow A, B or C.*) To a solution of **carboxylic acid** (2.5 or 3.0 equiv.) and DIEA (2.5 or 3.0 equiv.) in DMF or MeCN (6.4 mL/mmol of amine), a solution of triphosgene (0.4 or 0.5 equiv.) in MeCN (4.3 mL/mmol of amine) was added at 20 or 10 °C under argon (*caution*: large amounts of CO_2 and phosgene evolved). After being stirred at the same temperature for 30 s, a solution of **amine** (1.0

equiv.) in MeCN (7.1 mL/mmol of amine) was added at 20 or 10 °C under argon. After being stirred at the same temperature for 30 s, the reaction mixture was quenched with saturated aqueous NH_4Cl and the aqueous layer was extracted twice with $CHCl_3$. The combined organic layer was washed with 1 M HCl, saturated aqueous $NaHCO_3$ and brine, dried over $MgSO_4$, filtered and concentrated in vacuo. The residue was purified by column chromatography on silica gel to give a mixture of **desired product** and **epimer**. Yield was determined by HPLC-UV analysis.

Boc-L-Tyr(*O*-Bn)-L-Phe-OAllyl (5)

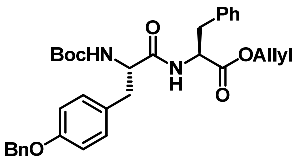

5

Chromatographic conditions: 2% MeOH in $CHCl_3$

HPLC conditions: DAICEL CHIRALPAK ID 4.6 mm×25 cm, 12% EtOH in hexane, flow rate 1 mL/min, detection wavelength 277 nm, retention time 16.13 min (epimer 21.37 min)

flow B: 81 mg, quant. (epimer <1%)

batch: 31 mg, 0.055 mmol, 40% (epimer <1%)

White solid; ^{1}H NMR (400 MHz, $CDCl_3$): δ 7.42-7.31 (m, 5H), 7.30-7.20 (m, 3H), 7.09 (d, *J* = 8.8 Hz, 2H), 7.00 (d, *J* = 7.8 Hz, 2H), 6.88 (d, *J* = 8.8 Hz, 2H), 6.35 (br, 1H), 5.81 (m, 1H), 5.28-5.21 (m, 2H), 5.01 (s, 2H), 4.99 (br, 1H), 4.80 (ddd, *J* = 5.8, 6.4, 6.8 Hz, 1H), 4.55 (m, 2H), 4.29 (br, 1H), 3.07 (dd, *J* = 6.3, 14.1 Hz, 1H), 3.03 (dd, *J* = 5.8, 13.7 Hz, 1H), 2.96 (m, 2H), 1.39 (s, 9H); ^{13}C NMR (100 MHz, $CDCl_3$): δ 170.8, 170.6, 157.8, 155.2, 136.9, 135.6, 131.3, 130.4, 129.2, 128.7, 128.5, 128.5, 127.9, 127.4, 127.0, 119.0, 115.0, 80.1, 70.0, 65.9, 55.7, 53.2, 37.9, 37.4, 28.2; IR (neat): 3333, 1742, 1664, 1513, 1244, 1178, 772, 749, 698 cm^{-1}; $[\alpha]^{25}_{D}$ = +20.4 (c 1.02, $CHCl_3$); mp 97-100 °C; HRMS (ESI-TOF): calcd for $[C_{33}H_{38}N_2O_6 + H]^+$ 559.2808, found 559.2813.

Fmoc-L-His(*N*-Trt)-L-Phe-OAllyl (6)

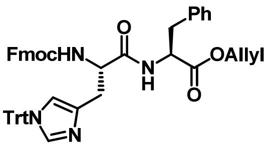

6

Chromatographic conditions: 2% MeOH in $CHCl_3$

HPLC conditions: DAICEL CHIRALPAK ID 4.6 mm×25 cm, 35% EtOAc in hexane, flow rate 1 mL/min, detection wavelength 265 nm, retention time 45.72 min (epimer 51.05 min)

flow B: 106 mg, 0.131 mmol, 92% (epimer 2%)

batch: 104 mg, 0.129 mmol, 75% (epimer 17%)

White solid; ^{1}H NMR (400 MHz, $CDCl_3$): δ 7.91 (brd, *J* = 4.9 Hz, 1H), 7.74 (d, *J* = 7.8 Hz, 2H), 7.58 (brd, *J* = 6.8 Hz, 2H), 7.39-7.24 (m, 15H), 7.20-7.08 (m, 10H), 6.71 (br, 1H), 6.67 (br, 1H), 5.76 (m, 1H), 5.22-5.14 (m, 2H), 4.84 (ddd, *J* = 6.4, 6.4, 6.8 Hz, 1H), 4.54-4.49 (m, 3H), 4.30 (d, *J* = 7.3 Hz, 2H), 4.20 (br, 1H), 3.12-2.93 (m, 4H); ^{13}C NMR (100 MHz, $CDCl_3$): δ 171.0, 170.7, 143.9, 143.9, 142.2, 141.2, 138.3, 136.8, 135.9, 131.4, 129.7, 129.3, 128.4, 128.0, 127.6, 127.0, 126.9, 125.2, 125.2, 120.0, 119.5, 118.7, 75.3, 67.2, 65.8, 54.8, 53.5, 47.1, 38.0, 30.5; IR (neat): 3307, 3064, 3031, 2948, 1728, 1674, 1520, 1496, 1449, 1246, 758, 702 cm^{-1}; $[\alpha]^{25}_D$ = +12.4 (c 1.02, $CHCl_3$); mp 81-83 °C; HRMS (ESI-TOF): calcd for $[C_{52}H_{46}N_4O_5 + H]^+$ 807.3546, found 807.3555.

Fmoc-L-Cys(*S*-Trt)-L-Phe-OAllyl (7)

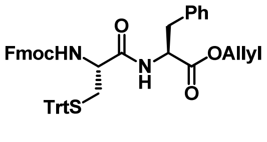

7

Chromatographic conditions: 25% EtOAc in hexane

HPLC conditions: DAICEL CHIRALPAK ID 4.6 mm×25 cm, 12% EtOH in hexane, flow rate 1 mL/min, detection wavelength 265 nm, retention time 19.78 min (epimer 26.77 min)

flow A: 101 mg, 0.131 mmol, 94% (epimer <1%)

batch: 78 mg, 0.101 mmol, 71% (epimer 1%)

White solid; ^{1}H NMR (400 MHz, $CDCl_3$): δ 7.73 (m, 2H), 7.55 (brd, *J* = 6.8 Hz, 2H), 7.41-7.34 (m, 8H), 7.28-7.11 (m, 14H), 7.03 (m, 2H), 6.33 (d, *J* = 7.8 Hz, 1H), 5.79 (m, 1H), 5.26-5.17 (m, 2H), 4.91 (brd, *J* = 6.8 Hz, 1H), 4.77 (ddd, *J* = 5.9, 6.4, 7.3 Hz, 1H), 4.53 (brd, *J* = 5.4 Hz, 2H), 4.37-4.27 (m, 2H), 4.17 (t, *J* = 6.8 Hz, 1H), 3.68 (br, 1H), 3.09 (dd, *J* = 5.9, 14.2 Hz, 1H), 3.03 (dd, *J* = 5.8, 14.2 Hz, 1H), 2.65 (dd, *J* = 7.8, 13.7 Hz, 1H), 2.59 (dd, *J* = 5.8, 13.7 Hz, 1H); ^{13}C NMR (100 MHz, $CDCl_3$): δ 170.4, 169.7, 155.7, 144.3, 143.6, 141.2, 135.5, 131.3, 129.5, 129.3, 128.4, 128.0, 127.7, 127.0, 126.9, 125.0, 125.0, 119.9, 119.1, 67.3, 67.0, 66.0, 53.4, 53.2, 47.0, 37.7, 33.4; IR (neat): 3309, 3063, 3031, 2931, 1740, 1668, 1520, 1447, 1250, 1216, 742, 758, 701 cm^{-1}; $[\alpha]^{25}_D$ = +11.0 (c 1.04, $CHCl_3$); mp 72-75 °C; HRMS (ESI-TOF): calcd for $[C_{49}H_{44}N_2O_5S + H]^+$ 773.3049, found 773.3067.

Boc-D-Phg-L-Phe-OAllyl (8)

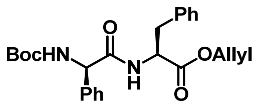

8

Chromatographic conditions: 2% MeOH in $CHCl_3$

HPLC conditions: DAICEL CHIRALPAK ID 4.6 mm×25 cm, 10% EtOH in hexane, flow rate 1 mL/min, detection wavelength 259 nm, retention time 20.10 min (epimer 16.62 min)

flow C: 63 mg, 97% (epimer 3%)

batch: 56 mg, 0.128 mmol, 74% (epimer 18%)

Colorless amorphous; ^{1}H NMR (400 MHz, $CDCl_3$): δ 7.35-7.30 (m, 5H), 7.14 (t, *J* = 7.3 Hz, 1H), 7.06 (dd, *J* = 7.8, 7.3 Hz, 2H), 6.66 (brd, *J* = 6.8 Hz, 2H), 6.16 (br, 1H), 5.86 (m, 1H), 5.80 (br, 1H), 5.33-5.25 (m, 2H), 5.13 (br, 1H), 4.91 (ddd, *J* = 5.4, 5.8, 7.4 Hz, 1H), 4.59 (brd, *J* = 5.8 Hz, 2H), 2.98 (m, 2H), 1.40 (s, 9H); ^{13}C NMR (100 MHz, $CDCl_3$): δ 170.6, 169.3, 154.9, 138.4, 135.0, 131.3, 129.1, 129.1, 128.4, 128.3, 127.2, 126.9, 119.2, 80.0, 66.2, 58.6, 53.0, 37.6, 28.2; IR (neat): 3312, 3021, 2979, 1740, 1716, 1663, 1497, 1216, 1170, 770, 700, 669 cm^{-1}; $[\alpha]^{18}_D$ = -14.1 (c 0.95, $CHCl_3$); HRMS (ESI-TOF): calcd for $[C_{25}H_{30}N_2O_5 + H]^+$ 439.2233, found 439.2235.

Boc-L-Ser(*O*-Bn)-L-Ile-OAllyl (9)

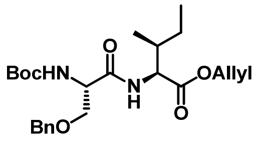

9

Chromatographic conditions: 2% MeOH in $CHCl_3$

HPLC conditions: DAICEL CHIRALPAK ID 4.6 mm×25 cm, 5% IPA in hexane, flow rate 1 mL/min, detection wavelength 258 nm, retention time 23.93 min (epimer 28.08 min)

flow B: 56 mg, 0.125 mmol, 89% (epimer <1%)

batch: 30 mg, 0.067 mmol, 47% (epimer 1%)

White solid; ^{1}H NMR (400 MHz, $CDCl_3$): δ 7.32 (m, 5H), 7.12 (br, 1H), 5.89 (m, 1H), 5.44 (br, 1H), 5.35-5.23 (m, 2H), 4.66-4.56 (m, 5H), 4.31 (br, 1H), 3.92 (m, 1H), 3.59 (m, 1H), 1.89 (m, 1H), 1.45 (s, 9H), 1.38 (m, 1H), 1.08 (m, 1H), 0.89-0.84 (m, 6H); ^{13}C NMR (100 MHz, $CDCl_3$): δ 171.1, 170.2, 155.4, 137.3, 131.6, 128.3, 127.8, 118.8, 80.1, 73.4, 69.9, 65.6, 56.7, 53.6, 37.7, 28.2, 24.9, 15.4, 11.4; IR (neat): 3841, 3737, 3294, 2969, 2935, 2877, 1732, 1708, 1651, 1509, 1367, 1245, 1168, 735, 698 cm^{-1}; $[\alpha]^{24}_D$ = +21.3 (c 1.02, $CHCl_3$); mp 75-76 °C; HRMS (ESI-TOF): calcd for $[C_{24}H_{36}N_2O_6 + H]^+$ 449.2652, found 449.2654.

Boc-L-Ser(*O*-Bn)-L-Pro-OAllyl (10)

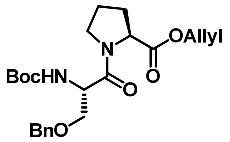

10

Chromatographic conditions: 2% MeOH in $CHCl_3$

HPLC conditions: DAICEL CHIRALPAK ID 4.6 mm×25 cm, 12% EtOH in hexane, flow rate 1 mL/min, detection wavelength 258 nm, retention time 13.88 min (epimer 19.77 min)

flow A: 49 mg, 0.113 mmol, 80% (epimer <1%)

batch: 36 mg, 0.083 mmol, 58% (epimer 1%)

Colorless oil; ^{1}H NMR (400 MHz, $CDCl_3$, major rotamer): δ 7.37-7.24 (m, 5H), 5.88 (m, 1H), 5.39 (brd, J = 8.3 Hz, 1H), 5.33-5.20 (m, 2H), 4.70 (ddd, J = 6.4, 6.4, 8.3 Hz, 1H), 4.64-4.43 (m, 5H), 3.77-3.63 (m, 4H), 2.22 (m, 1H), 2.00 (m, 3H), 1.43 (s, 9H); ^{13}C NMR (100 MHz, $CDCl_3$, mixture of rotamers): δ 171.3, 169.3, 155.3, 137.8, 131.8, 128.3, 127.6, 127.5, 127.4, 118.8, 118.3, 79.7, 73.3, 73.2, 71.7, 70.4, 66.2, 65.5, 59.1, 59.0, 52.0, 47.0, 46.4, 30.7, 29.0, 28.3, 24.8, 22.2; IR (neat): 3841, 3311, 2978, 2877, 1745, 1710, 1651, 1441, 1366, 1171, 1103, 741, 699 cm^{-1}; $[\alpha]^{24}_D$ = -41.9 (c 1.00, $CHCl_3$); HRMS (ESI-TOF): calcd for $[C_{23}H_{32}N_2O_6 + H]^+$ 433.2339, found 433.2334.

Boc-L-Ser(*O*-Bn)-Sar-OAllyl (11)

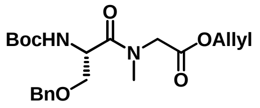

11

Chromatographic conditions: 30% EtOAc in hexane

HPLC conditions: DAICEL CHIRALPAK ID 4.6 mm×25 cm, 12% EtOH in hexane, flow rate 1 mL/min, detection wavelength 258 nm, retention time 12.67 min (epimer 17.30 min)

flow A: 42 mg, 0.103 mmol, 74% (epimer <1%)

batch: 30 mg, 0.074 mmol, 53% (epimer <1%)

Colorless oil; ^{1}H NMR (400 MHz, $CDCl_3$, major rotamer): δ 7.35-7.26 (m, 5H), 5.89 (m, 1H), 5.43 (brd, J = 8.3 Hz, 1H), 5.34-5.23 (m, 2H), 4.93 (ddd, J = 6.3, 6.3, 7.8 Hz, 1H), 4.63-4.48 (m, 4H), 4.15 (m, 2H), 3.64 (m, 2H), 3.14 (s, 3H), 1.44 (s, 9H); ^{13}C NMR (100 MHz, $CDCl_3$, mixture of rotamers): δ 171.3, 168.4, 155.1, 137.8, 131.5, 128.3, 127.6, 127.5, 118.7, 79.7, 73.2, 71.1, 70.6, 66.0, 65.7, 51.4, 50.1, 49.8, 36.6, 28.3; IR (neat): 3431, 2978, 2931, 1749, 1711, 1652, 1490, 1367, 1171, 773 cm^{-1}; $[\alpha]^{24}_D$ = +14.1 (c 0.98, $CHCl_3$); HRMS (ESI-TOF): calcd for $[C_{21}H_{30}N_2O_6 + H]^+$ 407.2182, found 407.2185.

L-Lac-L-Phe-OAllyl (12)

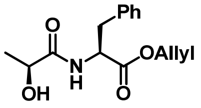

12

Chromatographic conditions: 4% MeOH in $CHCl_3$

HPLC conditions: DAICEL CHIRALPAK ID 4.6 mm×25 cm, 5% EtOH in hexane, flow rate 1 mL/min, detection wavelength 259 nm, retention time 15.95 min (epimer 18.15 min)

flow A: 76 mg, 0.274 mmol, 98% (epimer <1%)

batch: 22 mg, <28%

Colorless oil; ^{1}H NMR (400 MHz, $CDCl_3$): δ 7.30-7.22 (m, 3H), 7.12 (d, *J* = 7.8Hz, 2H), 6.92 (brd, *J* = 6.8 Hz, 1H), 5.87 (m, 1H), 5.34-5.25 (m, 2H), 4.90 (ddd, *J* = 6.4, 6.8, 7.8 Hz, 1H), 4.62 (d, *J* = 5.8 Hz, 2H), 4.20 (q, *J* = 6.8 Hz, 1H), 3.20 (dd, *J* = 5.8, 14.2 Hz, 1H), 3.10 (dd, *J* = 6.4, 14.2 Hz, 1H), 2.54 (br, 1H), 1.34 (d, *J* = 6.8 Hz, 3H); ^{13}C NMR (100 MHz, $CDCl_3$): δ 174.5, 171.3, 135.6, 131.3, 129.2, 128.5, 127.1, 119.0, 68.2, 66.1, 52.6, 37.9, 20.8; IR (neat): 3396, 1740, 1660, 1526, 1197, 1123, 938, 745, 702 cm^{-1}; $[\alpha]^{25}_D$ = +28.1 (c 0.99, $CHCl_3$); HRMS (ESI-TOF): calcd for $[C_{15}H_{19}N_2O_6 + H]^+$ 278.1392, found 278.1397.

8. Preparation of epimers

General procedure for the synthesis of epimers

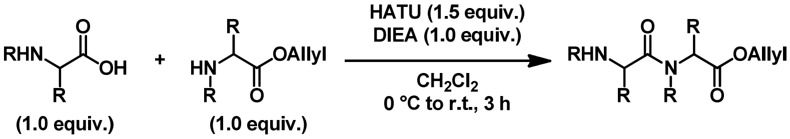

To a solution of **amine** (0.20 mmol, 1.0 equiv.) and **carboxylic acid** (0.20 mmol, 1.0 equiv.), DIEA (34 μL, 0.20 mmol, 1.0 equiv.) in CH_2Cl_2 (2.0 mL), *O*-(7-azabenzotriazol-1-yl)-1,1,3,3-tetramethyluronium hexafluorophosphate (HATU) (114 mg, 0.30 mmol, 1.5 equiv.) was added at 0 °C by using Zodiac® CCX-1101. After being stirred at room temperature for 3 h, the reaction mixture was diluted with CH_2Cl_2 (10 mL) and added 1 M HCl at 0 °C. The combined organic layer was washed with saturated aqueous $NaHCO_3$ and brine, dried over Na_2SO_4, filtered and concentrated in vacuo. The residue was purified by column chromatography on silica gel to give **product**.

Boc-D-Ser(*O*-Bn)-L-Phe-OAllyl (4)

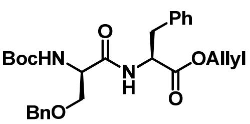

4

Chromatographic conditions: 25% EtOAc in hexane

95 mg, 0.196 mmol, 98%

Colorless oil; ^{1}H NMR (400 MHz, $CDCl_3$): δ 7.34-7.18 (m, 8H), 7.09 (d, *J* = 7.8 Hz, 2H), 6.96 (brd, *J* = 7.3 Hz, 1H), 5.82 (m, 1H), 5.36 (br, 1H), 5.30-5.21 (m, 2H), 4.90 (ddd, *J* = 6.4, 5.9, 7.8 Hz, 1H), 4.57 (d, *J* = 5.9 Hz, 2H), 4.50 (d, *J* = 12.2 Hz, 1H), 4.43 (d, *J* = 12.2 Hz, 1H), 4.30 (br, 1H), 3.85 (br, 1H), 3.51 (dd, *J* = 6.4, 9.8 Hz, 1H), 3.14 (dd, *J* = 5.9, 14.2 Hz, 1H), 3.08 (dd, *J* = 6.4, 14.2 Hz, 1H), 1.43 (s, 9H); ^{13}C NMR (100 MHz, $CDCl_3$): δ 170.7, 169.9, 155.4, 137.3, 135.6, 131.3, 129.2, 128.4, 128.3, 127.7, 127.6, 127.0, 118.9, 80.1, 73.2, 69.6, 65.9, 53.9, 53.2, 37.8, 28.2; IR (neat): 3321, 2979, 2933, 1742, 1716, 1674, 1497, 1367, 1248, 1169, 1112, 743, 700 cm^{-1}; $[\alpha]^{27}_{D}$ = +5.8 (c 1.17, $CHCl_3$); HRMS (ESI-TOF): calcd for $[C_{27}H_{34}N_2O_6 + H]^+$ 483.2495, found 483.2537.

Boc-D-Tyr(*O*-Bn)-L-Phe-OAllyl (S2)

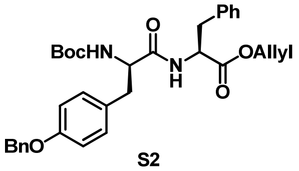

S2

Chromatographic conditions: 25% EtOAc in hexane

100 mg, 0.179 mmol, 89%

White solid; ^{1}H NMR (400 MHz, $CDCl_3$): δ 7.40-7.27 (m, 5H), 7.25-7.17 (m, 3H), 7.04 (d, *J* = 8.8 Hz, 2H), 6.97 (d, *J* = 7.3 Hz, 2H), 6.87 (d, *J* = 8.8 Hz, 2H), 6.56 (br, 1H), 5.80 (m, 1H), 5.28-5.19 (m, 2H), 5.06 (br, 1H), 5.00 (s, 2H), 4.85 (ddd, *J* = 6.3, 7.8, 5.8 Hz, 1H), 4.53 (d, *J* = 5.8 Hz, 2H), 4.35 (br, 1H), 3.03 (dd, *J* = 5.8, 14.2 Hz, 1H), 2.96 (dd, *J* = 6.3, 14.2 Hz, 1H), 2.92 (m, 2H), 1.37 (s, 9H); ^{13}C NMR (100 MHz, $CDCl_3$): δ 171.0, 170.7, 157.7, 155.2, 136.9, 135.5, 131.3, 130.3, 129.2, 128.8, 128.4, 127.8, 127.3, 127.0, 118.8, 114.9, 79.9, 69.9, 65.9, 55.7, 53.0, 37.8, 37.4, 28.2; IR (neat): 3318, 2978, 1740, 1661, 1513, 1368, 1244, 1177, 1024, 740, 699 cm^{-1}; $[\alpha]^{24}_{D}$ = +28.8 (c 1.01, $CHCl_3$); mp 117-119 °C; HRMS (ESI-TOF): calcd for $[C_{33}H_{38}N_2O_6 + H]^+$ 559.2808, found 559.2810.

Fmoc-D-His(*N*-Trt)-L-Phe-OAllyl (S3)

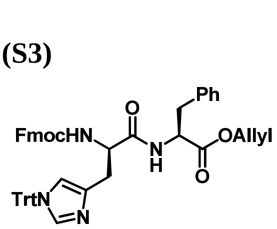

S3

Chromatographic conditions: 40% EtOAc in hexane

129 mg, 0.160 mmol, 80%

White solid; ^{1}H NMR (400 MHz, $CDCl_3$): δ 7.74 (d, *J* = 7.3 Hz, 2H), 7.69 (d, *J* = 7.8 Hz, 1H), 7.58 (br, 2H),7.38-7.34 (m, 3H), 7.29-7.22 (m, 11H), 7.16-7.07 (m, 11H), 6.88 (br, 1H), 6.62 (br, 1H), 5.80 (m, 1H), 5.26-5.16 (m, 2H), 4.86 (ddd, *J* = 6.4, 6.4, 7.3 Hz, 1H), 4.55-4.46 (m, 3H), 4.31 (m, 2H), 4.18 (br, 1H), 3.09 (br, 2H), 3.02 (dd, *J* = 5.4, 15.6 Hz, 1H), 2.93 (dd, *J* = 5.9, 14.7 Hz, 1H); ^{13}C NMR (100 MHz, $CDCl_3$): δ 170.9, 170.8, 156.1, 143.8, 142.2, 141.1, 138.4, 136.7, 135.8, 131.4, 129.6, 129.3, 128.3, 127.9, 127.6, 127.0, 126.9, 125.2, 125.1, 119.8, 119.4, 118.8, 75.2, 67.2, 65.8, 55.1, 53.2, 47.0, 37.9, 30.3; IR (neat): 3303, 3064, 3030, 1732, 1673, 1495, 1447, 1242, 757, 702 cm^{-1}; $[\alpha]^{24}_{D}$ = -7.0 (c 1.05, $CHCl_3$); mp 76-79 °C; HRMS (ESI-TOF): calcd for $[C_{52}H_{46}N_4O_5 + H]^+$ 807.3546, found 807.3541.

Fmoc-D-Cys(*S*-Trt)-L-Phe-OAllyl (S4)

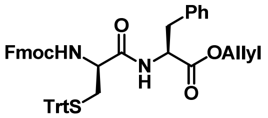

S4

Chromatographic conditions: 20-25% EtOAc in hexane

154 mg, 0.199 mmol, 99%

White solid; ^{1}H NMR (400 MHz, $CDCl_3$): δ 7.71 (dd, *J* = 6.8, 6.8 Hz, 2H), 7.53 (brd, *J* = 6.8 Hz, 2H), 7.40-7.32 (m, 8H), 7.23-7.16 (m, 11H), 7.16-7.00 (m, 5H), 6.26 (brd, *J* = 6.8 Hz, 1H), 5.80 (m, 1H), 5.26-5.17 (m, 3H), 4.79 (ddd, *J* = 5.8, 6.4, 7.3 Hz, 1H), 4.54 (d, *J* = 5.9 Hz, 2H), 4.35-4.25 (m, 2H), 4.14 (dd, *J* = 7.3, 6.8 Hz, 1H), 3.60 (br, 1H), 3.08 (dd, *J* = 5.9, 14.2 Hz, 1H), 3.02 (dd, *J* = 5.8, 14.2 Hz, 1H), 2.65 (dd, *J* = 8.3, 13.6 Hz, 1H), 2.56 (dd, *J* = 5.4, 13.6 Hz, 1H); ^{13}C NMR (100 MHz, $CDCl_3$): δ 170.4, 169.7, 155.7, 144.2, 143.7, 143.6, 141.1, 135.4, 131.2, 129.5, 129.2, 128.3, 128.0, 127.6, 127.0, 126.8, 125.0, 119.8, 119.0, 67.3, 67.0, 65.9, 53.9, 53.1, 46.9, 37.6, 34.1; IR (neat): 3316, 3062, 3030, 1732, 1668, 1516, 1447, 1248, 1215, 1197, 1034, 742, 701 cm^{-1}; $[\alpha]^{25}_{D}$ = +2.1 (c 1.02, $CHCl_3$); mp 67-71 °C; HRMS (ESI-TOF): calcd for $[C_{49}H_{44}N_2O_5S + H]^+$ 773.3049, found 773.3051.

Boc-L-Phg-L-Phe-OAllyl (S5)

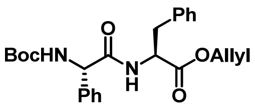

S5

Chromatographic conditions: 25% EtOAc in hexane

77 mg, 0.176 mmol, 88%

White solid; ^{1}H NMR (400 MHz, $CDCl_3$): δ 7.28-7.20 (m, 8H), 7.06 (d, *J* = 7.8 Hz, 2H), 6.41 (d, *J* = 7.8 Hz, 1H), 5.81-5.71 (m, 2H), 5.24-5.18 (m, 2H), 5.15 (br, 1H), 4.81 (ddd, *J* = 6.3, 6.4, 7.3

Hz, 1H), 4.50 (d, *J* = 5.9 Hz, 2H), 3.14 (dd, *J* = 5.9, 14.2 Hz, 1H), 3.05 (dd, *J* = 6.4, 14.2 Hz, 1H), 1.41 (s, 9H); ^{13}C NMR (100 MHz, $CDCl_3$): δ 170.5, 169.7, 155.0, 137.7, 135.5, 131.2, 129.2, 128.8, 128.5, 128.2, 127.1, 127.0, 118.9, 80.0, 65.9, 58.6, 53.4, 37.6, 28.2; IR (neat): 3319, 2979, 1743, 1716, 1663, 1497, 1367, 1247, 1170, 700 cm^{-1}; $[\alpha]^{25}_D$ = +64.2 (c 1.03, $CHCl_3$); mp 133-135 °C; HRMS (ESI-TOF): calcd for $[C_{25}H_{30}N_2O_5 + H]^+$ 439.2233, found 439.2247.

Boc-D-Ser(*O*-Bn)-L-Ile-OAllyl (S6)

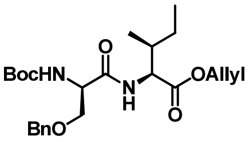

S6

Chromatographic conditions: 25% EtOAc in hexane

112 mg, quant.

Colorless oil; ^{1}H NMR (400 MHz, $CDCl_3$): δ 7.31 (m, 5H), 7.12 (brd, *J* = 8.3 Hz, 1H), 5.88 (m, 1H), 5.45 (br, 1H), 5.35-5.22 (m, 2H), 4.61 (m, 3H), 4.58 (d, *J* = 12.7 Hz, 1H), 4.50 (d, *J* = 12.2 Hz, 1H), 4.31 (br, 1H), 3.89 (br, 1H), 3.61 (dd, *J* = 6.4, 9.3 Hz, 1H), 1.90 (m, 1H), 1.45 (s, 9H), 1.39 (m, 1H), 1.09 (m, 1H), 0.89-0.85 (m, 6H); ^{13}C NMR (100 MHz, $CDCl_3$): δ 171.1, 169.9, 155.3, 137.3, 131.5, 128.3, 127.8, 127.7, 118.7, 80.1, 73.4, 69.7, 65.6, 56.5, 54.0, 37.8, 28.2, 24.8, 15.3, 11.4; IR (neat): 3319, 2969, 2935, 2878, 1739, 1716, 1668, 1515, 1498, 1367, 1250, 1170, 1107, 738, 699 cm^{-1}; $[\alpha]^{26}_D$ = +0.3 (c 1.20, $CHCl_3$); HRMS (ESI-TOF): calcd for $[C_{24}H_{36}N_2O_6 + H]^+$ 449.2652, found 449.2657.

Boc-D-Ser(*O*-Bn)-L-Pro-OAllyl (S7)

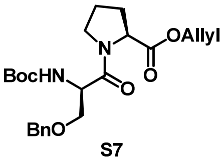

S7

Chromatographic conditions: 30% EtOAc in hexane

80 mg, 0.185 mmol, 92%

Colorless oil; ^{1}H NMR (400 MHz, $CDCl_3$, major rotamer): δ 7.27 (m, 5H), 5.89 (m, 1H), 5.40 (brd, *J* = 6.4 Hz, 1H), 5.34-5.21 (m, 2H), 4.72 (m, 1H), 4.61 (br, 2H), 4.53-4.46 (m, 3H), 3.70-3.57 (m, 4H), 2.13 (m, 1H), 1.98 (m, 2H), 1.81 (m, 1H), 1.42 (s, 9H); ^{13}C NMR (100 MHz, $CDCl_3$, major rotamer): δ 171.4, 169.3, 155.0, 137.8, 131.7, 128.2, 127.6, 127.4, 118.2, 79.6, 73.2, 70.8, 65.5, 59.0, 51.6, 47.0, 29.1, 28.2, 24.5; IR (neat): 2978, 1746, 1713, 1646, 1446, 1172 cm^{-1}; $[\alpha]^{26}_D$ = -40.1 (c 0.95, $CHCl_3$); HRMS (ESI-TOF): calcd for $[C_{23}H_{32}N_2O_6 + H]^+$

433.2339, found 433.2341.

Boc-D-Ser(*O*-Bn)-Sar-OAllyl (S8)

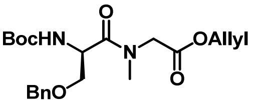

S8

Chromatographic conditions: 30% EtOAc in hexane

69 mg, 0.170 mmol, 85%

Colorless oil; ^{1}H NMR (400 MHz, $CDCl_3$, major rotamer): δ 7.35-7.26 (m, 5H), 5.89 (m, 1H), 5.43 (brd, *J* = 8.3 Hz, 1H), 5.33-5.22 (m, 2H), 4.93 (ddd, *J* = 6.3, 6.4, 7.3 Hz, 1H), 4.63-4.48 (m, 4H), 4.15 (m, 2H), 3.64 (m, 2H), 3.14 (s, 3H), 1.43 (s, 9H); ^{13}C NMR (100 MHz, $CDCl_3$, mixture of rotamers): δ 171.3, 168.4, 155.1, 137.8, 131.5, 128.3, 127.6, 127.5, 118.7, 79.7, 73.2, 70.6, 66.0, 65.7, 50.1, 49.7, 36.6, 28.3; IR (neat): 2979, 2935, 1749, 1713, 1652, 1495, 1367, 1172 cm^{-1}; $[\alpha]^{26}_{D}$ = -11.7 (c 1.32, $CHCl_3$); HRMS (ESI-TOF): calcd for $[C_{21}H_{30}N_2O_6 + H]^+$ 407.2182, found 407.2185.

DL-Lac-L-Phe-OAllyl (S9)

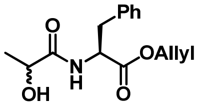

S9

Chromatographic conditions: 4% MeOH in $CHCl_3$

54 mg, 0.195 mmol, 98%

Colorless oil; ^{1}H NMR (400 MHz, $CDCl_3$, mixture of diastereomers): δ 7.30-7.22 (m, 6H), 7.12 (d, *J* = 7.8Hz, 4H), 6.94 (brd, *J* = 7.8 Hz, 1H), 6.85 (brd, *J* = 7.8 Hz, 1H), 5.87 (m, 2H), 5.33-5.25 (m, 4H), 4.90 (m, 2H), 4.61 (d, *J* = 5.9 Hz, 4H), 4.18 (m, 2H), 3.15 (m, 4H), 1.37 (d, *J* = 6.8 Hz, 3H), 1.33 (d, *J* = 6.8 Hz, 3H); ^{13}C NMR (100 MHz, $CDCl_3$, mixture of diastereomers): δ 174.3, 174.2, 171.3, 171.1, 135.7, 135.6, 131.3, 129.3, 129.2, 128.6, 128.5, 127.1, 119.1, 119.1, 68.3, 68.2, 66.1, 66.1, 52.8, 52.7, 37.9, 37.9, 21.0, 21.0; IR (neat): 3394, 1742, 1660, 1525, 1274, 1196, 1122, 702 cm^{-1}; HRMS (ESI-TOF): calcd for $[C_{15}H_{19}N_2O_6 + H]^+$ 278.1392, found 278.1391.

9. In situ IR analysis

9.1. General procedure for in situ IR analysis

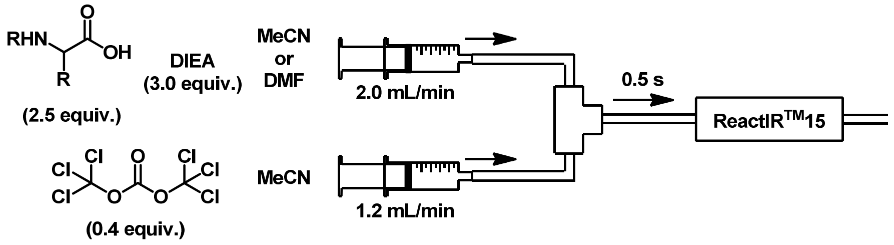

A solution of **carboxylic acid** (0.35 M, 2.5 equiv.) and DIEA (0.42 M, 3.0 equiv.) in MeCN or DMF (flow rate: 2.0 mL/min) and a solution of triphosgene (0.092 M, 0.4 equiv.) in MeCN (flow rate: 1.2 mL/min) were introduced to T-shape mixer 1 at room temperature with the syringe pumps. The resultant mixture was passed through reaction tube 1 (inner diameter: 0.8 mm, length: 54 mm, volume: 27 μL, reaction time: 0.5 s) at the same temperature. After being eluted for 40 s to reach a steady state, the resultant mixture was analyzed by Mettler-Toledo React IR™ 15 Micro-flow cell.

9.2. Synthesis of reference compounds

Fmoc-L-Val-Cl (S10)

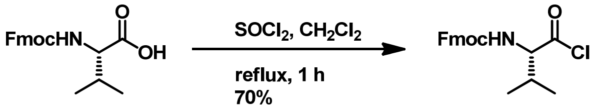

14 S10

To a solution of Fmoc-L-Val-OH (**14**) (300 mg, 0.84 mmol, 1.0 equiv.) in CH_2Cl_2 (5 mL), $SOCl_2$ (609 μL, 8.4 mmol, 10 equiv.) was added at room temperature under argon. After being stirred at reflux temperature for 1 h, the reaction mixture was cooled and concentrated in vacuo. The residue was recrystallized from hexane/CH_2Cl_2 to give Fmoc-L-Val-Cl[1] (**S10**) (212 mg, 0.592 mmol, 70%) as a white solid.

^{1}H NMR (400 MHz, $CDCl_3$): δ 7.76 (d, J = 7.8 Hz, 2H), 7.58 (brd, J = 6.8 Hz, 2H), 7.40 (dd, J = 7.3, 7.4 Hz, 2H), 7.31 (dd, J = 7.3, 7.3 Hz, 2H), 5.19 (brd, J = 8.3 Hz, 1H), 4.52-4.42 (m, 3H), 4.23 (t, J = 6.3 Hz, 1H), 2.40 (m, 1H), 1.04 (d, J = 6.4 Hz, 3H), 0.94 (d, J = 5.9 Hz, 3H); ^{13}C NMR (100 MHz, $CDCl_3$): δ 174.6, 156.0, 143.6, 141.3, 127.8, 127.1, 124.9, 120.0, 67.9, 67.3, 47.1, 29.9, 19.3, 17.0; IR (neat): 3335, 2961, 1720, 1521, 1451, 1314, 1240, 1101, 1026, 726 cm^{-1}; $[\alpha]^{19}_D$ = -6.7 (c 1.00, CH_2Cl_2); mp 95-97 °C.

[1] L. A. Carpino, B. Cohen, K. E. Stephens Jr., S. Y. Sadat-Aalaee, J-H. Tien, D. C. Langridge, *J. Org. Chem.* **1986**, *51*, 3732-3734.

(Fmoc-L-Val)$_2$O (S11)

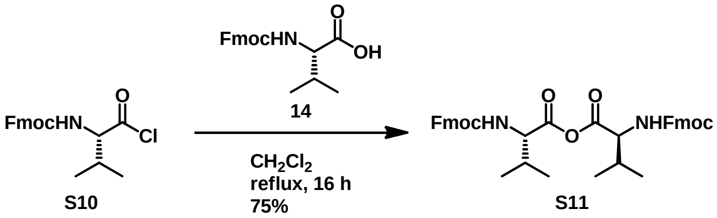

To Fmoc-L-Val-Cl (**S10**) (50 mg, 0.140 mmol, 1.0 equiv.) and Fmoc-L-Val-OH (**14**) (50 mg, 0.140 mmol, 1.0 equiv.), CH_2Cl_2 (1.0 mL) was added at room temperature under argon and stirred at reflux temperature. The reaction was monitored by IR. After being stirred at reflux temperature for 16 h, the reaction mixture was cooled and concentrated in vacuo. The residue was recrystallized from hexane/CH_2Cl_2 to give (Fmoc-L-Val-)$_2$O[2] (**S11**) (70 mg, 0.106 mmol, 75%) as a white solid.

^{1}H NMR (400 MHz, $CDCl_3$): δ 7.75 (d, J = 7.3 Hz, 4H), 7.57 (brd, J = 6.8 Hz, 4H), 7.39 (dd, J = 7.3, 7.3 Hz, 4H), 7.30 (dd, J = 6.8, 7.3 Hz, 4H), 5.26 (brd, J = 8.8 Hz, 2H), 4.49-4.37 (m, 6H), 4.22 (t, J = 6.8 Hz, 2H), 2.25 (m, 2H), 1.03 (d, J = 6.4 Hz, 6H), 0.96 (d, J = 7.3 Hz, 6H); ^{13}C NMR (100 MHz, $CDCl_3$): δ 167.5, 156.2, 143.7, 141.3, 127.7, 127.1, 125.0, 120.0, 67.3, 59.6, 47.1, 30.7, 19.1, 17.3; IR (neat): 3322, 3054, 2967, 1825, 1712, 1520, 1451, 1266, 1046, 738, 705 cm^{-1}; $[\alpha]^{19}_D$ = -3.5 (c 1.00, CH_2Cl_2); mp 163-165 °C; HRMS (ESI-TOF): calcd for $[C_{40}H_{40}N_2O_7 + H]^+$ 661.2914, found 661.2917.

9.3. IR spectra

In situ IR

Carboxylic acid: Fmoc-L-Val-OH

Solvent: MeCN

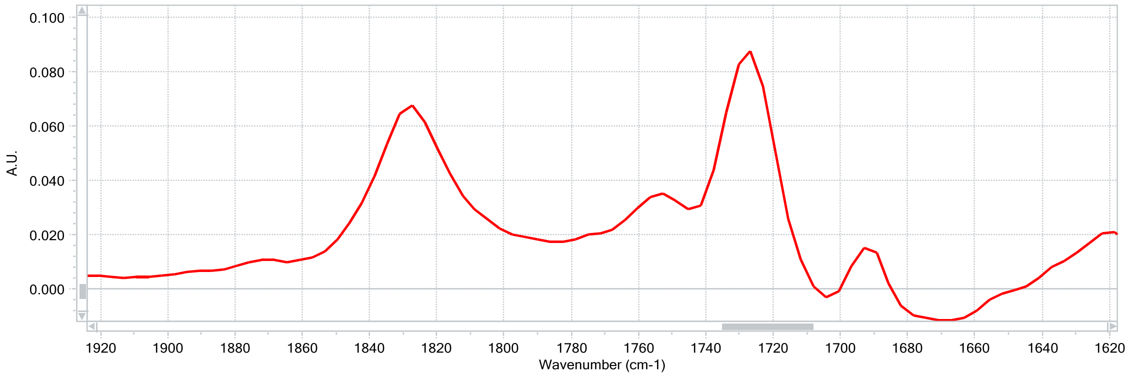

[2] A. Paquet, F. M. F. Chen, N. L. Benoiton, *Can. J. Chem.* **1984**, *62*, 1335-1338.

In situ IR

Carboxylic acid: Fmoc-L-Val-OH

Solvent: DMF/MeCN

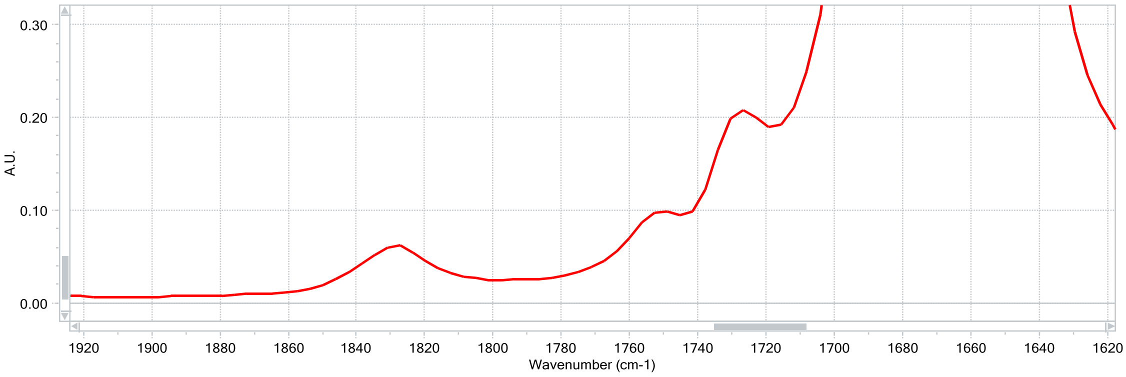

Fmoc-L-Val-Cl (**S10**) in MeCN

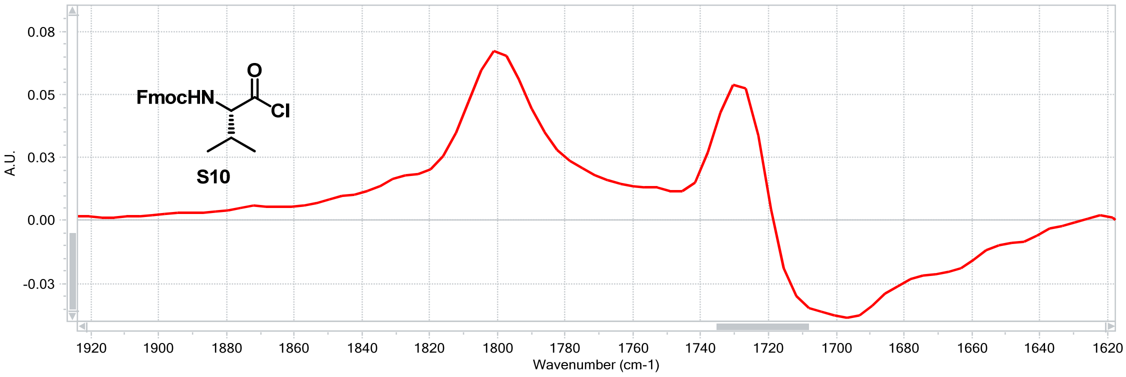

(Fmoc-L-Val)$_2$O (**S11**) in CH_2Cl_2

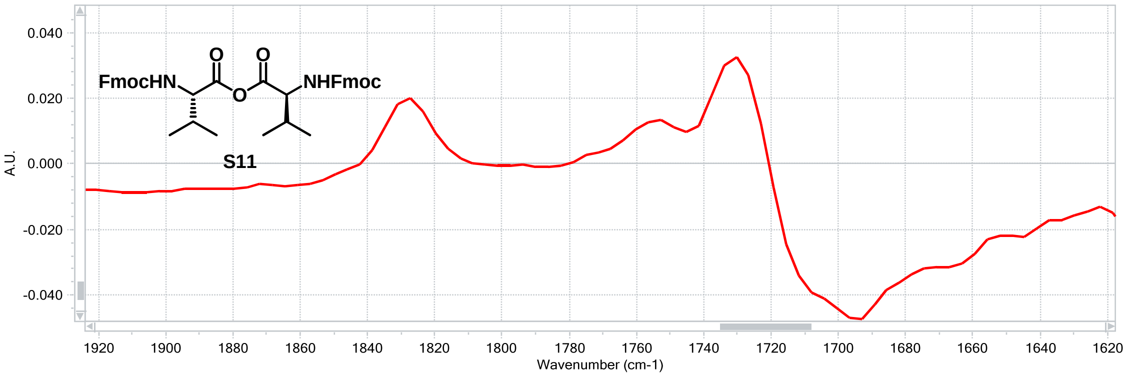

In situ IR spectra were in good agreement with reference symmetric anhydride **S11**. In situ IR spectra using other carboxylic acid were shown below.

Carboxylic acid: Boc-L-Ser(*O*-Bn)-OH

Solvent: MeCN

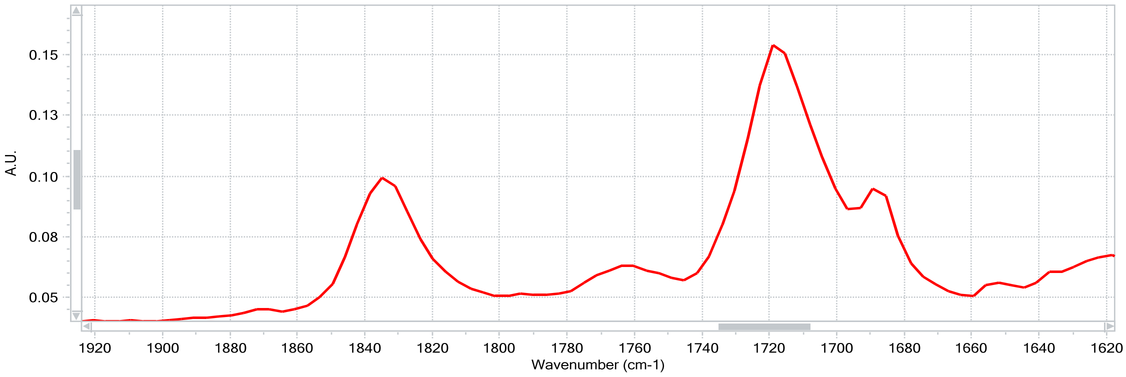

Carboxylic acid: Boc-L-Tyr(*O*-Bn)-OH

Solvent: MeCN

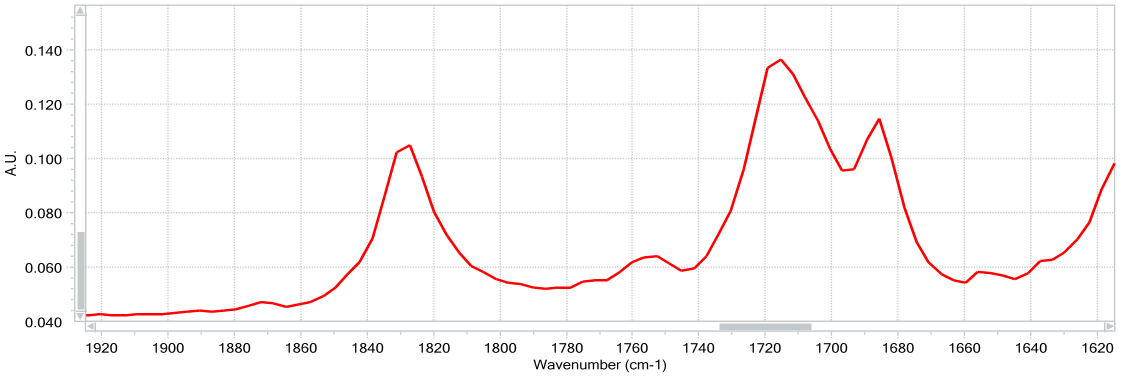

Carboxylic acid: Fmoc-L-His(*N*-Trt)-OH

Solvent: DMF/MeCN

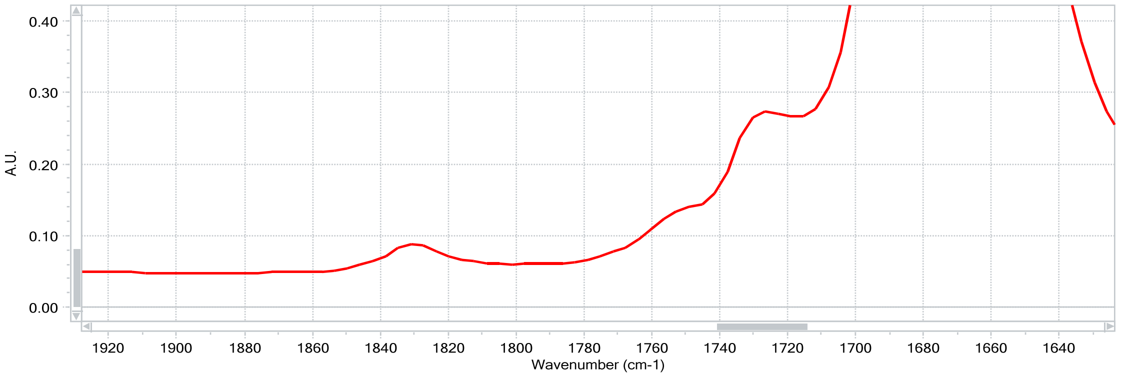

Carboxylic acid: Fmoc-L-Cys(*S*-Trt)-OH

Solvent: MeCN

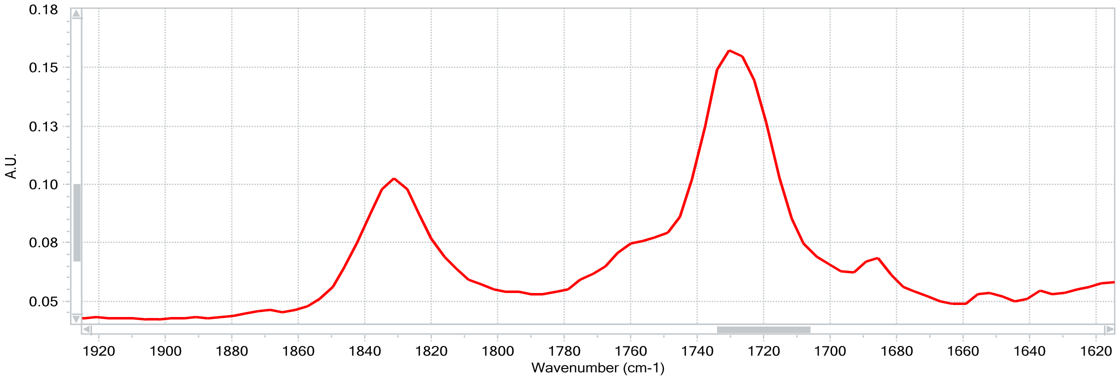

Carboxylic acid: Boc-D-Phg-OH

Solvent: MeCN

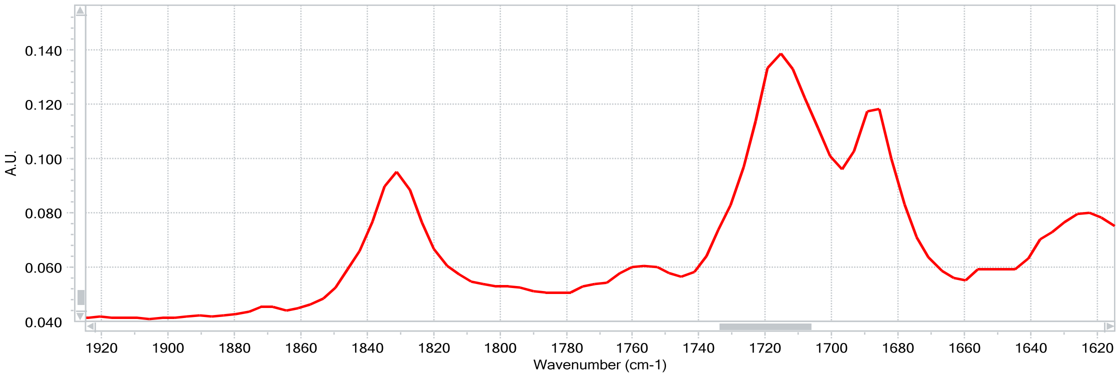

Carboxylic acid: L-Lac-OH

Solvent: MeCN

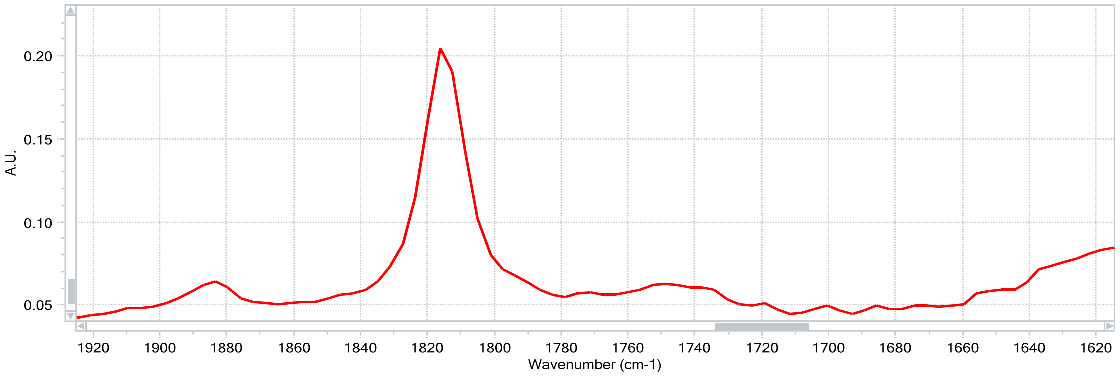

10. Synthesis of tetrapeptide moiety of aurilide (13)

10.1. Linear route

Fmoc-L-Val-OAllyl (S12)

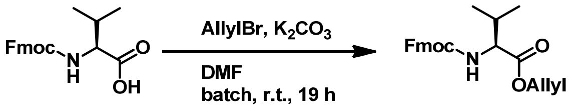

14 **S12**

To a suspension of Fmoc-L-Val-OH (**14**) (5.0 g, 14.7 mmol, 1.0 equiv.) and K_2CO_3 (3.1 g, 22.3 mmol, 1.5 equiv.) in DMF (36.8 mL), allyl bromide (1.37 mL, 16.2 mmol, 1.1 equiv.) was added at room temperature under argon. After being stirred at the same temperature for 19 h, the reaction mixture was filtered through a pad of Celite, acidified with 1 M HCl at 0 °C and the aqueous layer was extracted with EtOAc. The combined organic layer was washed with 10% aqueous $Na_2S_2O_3$, saturated aqueous $NaHCO_3$ and brine, dried over $MgSO_4$, filtered and concentrated in vacuo. The residue was purified by column chromatography on silica gel (15% EtOAc in hexane) to give Fmoc-L-Val-OAllyl (**S12**) (5.18 g, 13.7 mmol, 93%) as a colorless oil.

^{1}H NMR (400 MHz, $CDCl_3$): δ 7.74 (d, *J* = 7.8 Hz, 2H), 7.59 (d, *J* = 7.3 Hz, 2H), 7.38 (dd, *J* = 7.3, 7.8 Hz, 2H), 7.29 (dd, *J* = 7.8, 7.3 Hz, 2H), 5.90 (m, 1H), 5.37-5.23 (m, 3H), 4.64 (m, 2H), 4.39 (brd, *J* = 6.8 Hz, 2H), 4.34 (dd, *J* = 4.9, 9.3 Hz, 1H), 4.22 (t, *J* = 6.8 Hz, 1H), 2.19 (m, 1H), 0.98 (d, *J* = 6.8 Hz, 3H), 0.91 (d, *J* = 6.8 Hz, 3H); ^{13}C NMR (100 MHz, $CDCl_3$): δ 171.8, 156.2, 143.8, 141.3, 131.5, 127.7, 127.0, 125.0, 119.9, 119.0, 67.0, 65.8, 59.0, 47.2, 31.3, 18.9, 17.5; IR (neat): 3345, 2965, 1725, 1521, 1451, 1239, 1195, 1095, 1032, 990, 759, 740 cm^{-1}; $[\alpha]^{22}_D$ = -3.6 (c 1.35, $CHCl_3$); HRMS (ESI-TOF): calcd for $[C_{23}H_{25}NO_4 + H]^+$ 380.1862, found 380.1867.

Fmoc-Sar-L-Val-OAllyl (15)

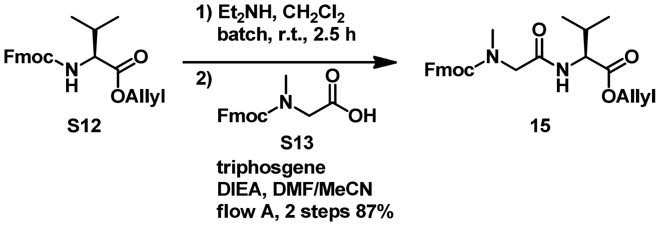

S12 **15**

To a solution of Fmoc-L-Val-OAllyl (**S12**) (2.59 g, 6.83 mmol, 1.0 equiv.) in CH_2Cl_2 (7.0 mL), diethylamine (7.0 mL) was added at room temperature under argon. After being stirred at the same temperature for 2.5 h, the reaction mixture was concentrated in vacuo. The residue was azeotroped twice with toluene and used for the next reaction without further purification.

A solution of Fmoc-Sar-OH (**S13**) (0.35 M, 2.5 equiv.) and DIEA (0.42 M, 3.0 equiv.) in DMF (flow rate: 2.0 mL/min) and a solution of triphosgene (0.092 M, 0.4 equiv.) in MeCN (flow rate: 1.2 mL/min) were introduced to T-shape mixer 1 at 20 °C with the syringe pumps. The resultant

mixture was passed through reaction tube 1 (inner diameter: 0.8 mm, length: 54 mm, volume: 27 μL, reaction time: 0.5 s) at the same temperature. Then, the resultant mixture and a solution of crude amine (0.14 M, 1.0 equiv.) in MeCN (flow rate: 2.0 mL/min) were introduced to T-shape mixer 2 at 20 °C. The resultant mixture was passed through reaction tube 2 (inner diameter: 0.8 mm, length: 742 mm, volume: 373 μL, reaction time: 4.3 s) at the same temperature. After being eluted for 40 s to reach a steady state, the resultant mixture was poured into saturated aqueous NH_4Cl, brine and $CHCl_3$ suspension for 23 min 44 s at room temperature. The aqueous layer was extracted twice with $CHCl_3$. The combined organic layer was washed with 1 M HCl, saturated aqueous $NaHCO_3$ and brine, dried over $MgSO_4$, filtered and concentrated in vacuo. The residue was purified by column chromatography on silica gel (1.5-2% MeOH in $CHCl_3$) to give Fmoc-Sar-L-Val-OAllyl (**15**) (2.60 g, 5.77 mmol, 2 steps 87%) as a colorless oil.

^{1}H NMR (400 MHz, $CDCl_3$, major rotamer): δ 7.76 (d, J = 7.3 Hz, 2H), 7.58 (br, 2H), 7.39 (dd, J = 7.3, 7.8 Hz, 2H), 7.30 (dd, J = 7.3, 7.3 Hz, 2H), 6.58 (br, 1H), 5.87 (m, 1H), 5.33-5.22 (m, 2H), 4.60 (br, 3H), 4.44 (br, 2H), 4.27 (br, 1H), 4.05-3.91 (m, 2H), 3.09 (s, 3H), 2.19 (m, 1H), 0.93 (d, J = 6.7 Hz, 3H), 0.87 (d, J = 6.8 Hz, 3H); ^{13}C NMR (100 MHz, $CDCl_3$, mixture of rotamers): δ 171.3, 168.8, 157.0, 143.8, 141.3, 131.7, 131.5, 127.7, 127.1, 124.9, 120.0, 119.0, 118.8, 68.1, 65.9, 65.7, 56.9, 56.5, 54.6, 53.3, 47.1, 43.7, 36.7, 35.6, 31.2, 19.1, 19.0, 17.6; IR (neat): 3322, 2965, 1740, 1706, 1534, 1451, 1405, 1224, 1148, 740 cm^{-1}; $[\alpha]^{24}_D$ = +6.3 (c 0.40, $CHCl_3$); HRMS (ESI-TOF): calcd for $[C_{26}H_{30}N_2O_5 + H]^+$ 451.2233, found 451.2234.

Fmoc-D-(*N*-Me)Leu-Sar-L-Val-OAllyl (16)

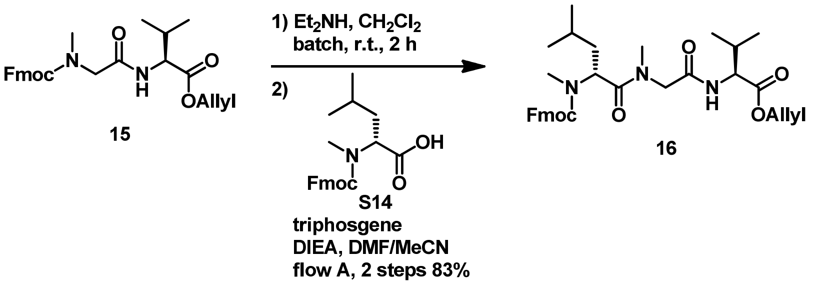

To a solution of Fmoc-Sar-L-Val-OAllyl (**15**) (369 mg, 0.819 mmol, 1.0 equiv.) in CH_2Cl_2 (1.6 mL), diethylamine (1.6 mL) was added at room temperature under argon. After being stirred at the same temperature for 2 h, the reaction mixture was concentrated in vacuo. The residue was azeotroped twice with toluene and used for the next reaction without further purification.

A solution of Fmoc-D-(*N*-Me)Leu-OH (**S14**) (0.35 M, 2.5 equiv.) and DIEA (0.42 M, 3.0 equiv.) in DMF (flow rate: 2.0 mL/min) and a solution of triphosgene (0.092 M, 0.4 equiv.) in MeCN (flow rate: 1.2 mL/min) were introduced to T-shape mixer 1 at 20 °C with the syringe

pumps. The resultant mixture was passed through reaction tube 1 (inner diameter: 0.8 mm, length: 54 mm, volume: 27 μL, reaction time: 0.5 s) at the same temperature. Then, the resultant mixture and a solution of crude amine (0.14 M, 1.0 equiv.) in MeCN (flow rate: 2.0 mL/min) were introduced to T-shape mixer 2 at 20 °C. The resultant mixture was passed through reaction tube 2 (inner diameter: 0.8 mm, length: 742 mm, volume: 373 μL, reaction time: 4.3 s) at the same temperature. After being eluted for 40 s to reach a steady state, the resultant mixture was poured into saturated aqueous NH_4Cl, brine and $CHCl_3$ suspension for 137 s at room temperature. The aqueous layer was extracted twice with $CHCl_3$. The combined organic layer was washed with 1 M HCl, saturated aqueous $NaHCO_3$ and brine, dried over $MgSO_4$, filtered and concentrated in vacuo. The residue was purified by column chromatography on silica gel (2% MeOH in $CHCl_3$) to give Fmoc-D-(*N*-Me)Leu-Sar-L-Val-OAllyl (**16**) (308 mg, 0.533 mmol, 2 steps 83%, epimer <1%) as a colorless amorphous. Yield was determined by HPLC-UV analysis (conditions: DAICEL CHIRALCEL OD-H 4.6 mm×25 cm, 4% EtOH in hexane, flow rate 1 mL/min, detection wavelength 266 nm, retention time 32.45 min, epimer 37.93 min).

^{1}H NMR (400 MHz, $CDCl_3$, major rotamer): δ 7.74 (d, *J* = 7.3 Hz, 2H), 7.59 (d, *J* = 7.3 Hz, 2H), 7.38 (dd, *J* = 7.3, 7.8 Hz, 2H), 7.29 (dd, *J* = 7.3, 7.3 Hz, 2H), 6.71 (d, *J* = 8.8 Hz, 1H), 5.86 (m, 1H), 5.32-5.16 (m, 3H), 4.59 (br, 2H), 4.51 (brt, *J* = 4.4 Hz, 1H), 4.44 (m, 2H), 4.23 (m, 1H), 4.06 (d, *J* = 18.5 Hz, 1H), 4.00 (d, *J* = 15.2 Hz, 1H), 3.14 (s, 3H), 2.88 (s, 3H), 2.16 (br, 1H), 1.74-1.11 (m, 3H), 0.96-0.82 (m, 12H); ^{13}C NMR (100 MHz, $CDCl_3$, mixture of rotamers): δ 172.4, 171.1, 168.5, 156.6, 143.8, 143.7, 141.3, 131.5, 127.6, 127.2, 127.0, 124.9, 124.1, 119.9, 118.8, 67.5, 65.8, 65.6, 57.0, 52.6, 52.5, 52.2, 47.2, 37.7, 36.5, 35.4, 30.9, 29.5, 29.3, 24.6, 24.4, 22.9, 22.4, 22.2, 21.9, 18.9, 17.5, 17.4; IR (neat): 3323, 2960, 1741, 1695, 1658, 1533, 1452, 1397, 1310, 1195, 1157, 758, 741 cm^{-1}; $[\alpha]^{24}_D$ = +54.7 (c 1.29, $CHCl_3$); HRMS (ESI-TOF): calcd for $[C_{33}H_{43}N_3O_6 + H]^+$ 578.3230, found 578.3232.

Fmoc-L-Val-D-(*N*-Me)Leu-Sar-L-Val-OAllyl (17)

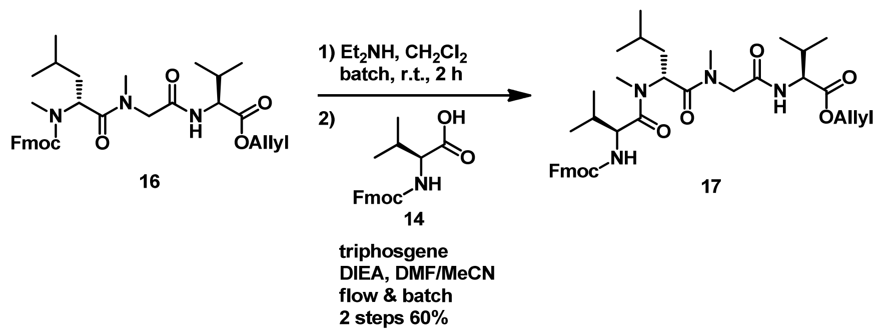

To a solution of Fmoc-D-(*N*-Me)Leu-Sar-L-Val-OAllyl (**16**) (335 mg, 0.580 mmol, 1.0 equiv.) in CH_2Cl_2 (0.9 mL), diethylamine (0.9 mL) was added at room temperature under argon. After being stirred at the same temperature for 2 h, the reaction mixture was concentrated in vacuo.

The residue was azeotroped twice with toluene and used for the next reaction without further purification.

A solution of Fmoc-L-Val-OH (**14**) (0.35 M, 2.5 equiv.) and DIEA (0.42 M, 3.0 equiv.) in DMF (flow rate: 2.0 mL/min) and a solution of triphosgene (0.092 M, 0.4 equiv.) in MeCN (flow rate: 1.2 mL/min) were introduced to T-shape mixer 1 at 20 °C with the syringe pumps. The resultant mixture was passed through reaction tube 1 (inner diameter: 0.8 mm, length: 54 mm, volume: 27 μL, reaction time: 0.5 s) at the same temperature. Then, the resultant mixture and a solution of crude amine (0.14 M, 1.0 equiv.) in MeCN (flow rate: 2.0 mL/min) were introduced to T-shape mixer 2 at 20 °C. The resultant mixture was passed through reaction tube 2 (inner diameter: 0.8 mm, length: 2224 mm, volume: 1118 μL, reaction time: 12.9 s) at the same temperature. After being eluted for 40 s to reach a steady state, the resultant mixture was poured into dry flask for 73 s at room temperature. After being stirred at the same temperature for 10 min, the reaction mixture was quenched with saturated aqueous NH_4Cl. The aqueous layer was extracted twice with $CHCl_3$. The combined organic layer was washed with 1 M HCl, saturated aqueous $NaHCO_3$ and brine, dried over $MgSO_4$, filtered and concentrated in vacuo. The residue was purified by column chromatography on silica gel (2% MeOH in $CHCl_3$) to give Fmoc-L-Val-D-(*N*-Me)Leu-Sar-L-Val-OAllyl (**17**) (139 mg, 0.205 mmol, 2 steps 60%, epimer <1%) as a colorless oil. Yield was determined by HPLC-UV analysis (conditions: DAICEL CHIRALPAK ID 4.6 mm×25 cm, 10% EtOH in hexane, flow rate 1 mL/min, detection wavelength 266 nm, retention time 24.40 min, epimer 29.52 min).

^{1}H NMR (400 MHz, $CDCl_3$, major rotamer): δ 7.75 (d, *J* = 7.3 Hz, 2H), 7.59 (d, *J* = 7.3 Hz, 2H), 7.39 (dd, *J* = 7.3, 7.3 Hz, 2H), 7.30 (dd, *J* = 6.3, 6.8 Hz, 2H), 6.68 (d, *J* = 8.8 Hz, 1H), 5.86 (m, 1H), 5.73 (d, *J* = 9.3 Hz, 1H), 5.48 (dd, *J* = 6.4, 8.3 Hz, 1H), 5.31-5.20 (m, 2H), 4.56 (m, 4H), 4.42 (dd, *J* = 7.3, 10.2 Hz, 1H), 4.29 (dd, *J* = 7.3, 10.2 Hz, 1H), 4.21 (t, *J* = 6.8 Hz, 1H), 4.07 (d, *J* = 15.1 Hz, 1H), 3.98 (d, *J* = 15.1 Hz, 1H), 3.15 (s, 3H), 3.11 (s, 3H), 2.18 (m, 1H), 2.01 (m, 1H), 1.75-1.41 (m, 3H), 0.98-0.86 (m, 18H); ^{13}C NMR (100 MHz, $CDCl_3$, mixture of rotamers): δ 172.5, 172.1, 171.3, 168.3, 156.3, 143.9, 143.8, 141.3, 131.5, 127.6, 127.1, 127.0, 125.1, 125.0, 120.0, 119.9, 118.9, 118.8, 66.9, 65.7, 65.6, 62.5, 61.9, 57.0, 56.7, 56.1, 52.5, 51.1, 47.2, 45.6, 43.0, 37.7, 36.6, 31.2, 31.1, 30.8, 24.9, 23.1, 21.9, 19.6, 19.0, 18.9, 17.7, 17.5, 17.0; IR (neat): 3317, 2963, 1721, 1694, 1635, 1531, 1241, 1196, 742 cm^{-1}; $[\alpha]^{24}_D$ = +29.6 (c 1.01, $CHCl_3$); HRMS (ESI-TOF): calcd for $[C_{38}H_{52}N_4O_7 + H]^+$ 677.3914, found 677.3912.

10.2. Convergent route

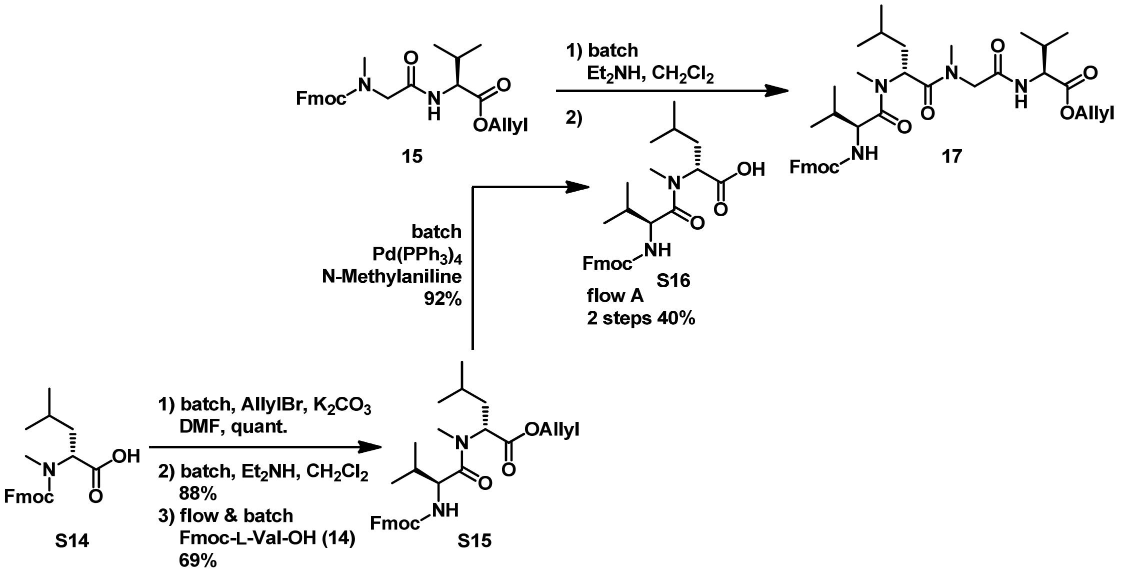

Scheme S-1

Fmoc-D-(*N*-Me)Leu-OAllyl (S17)

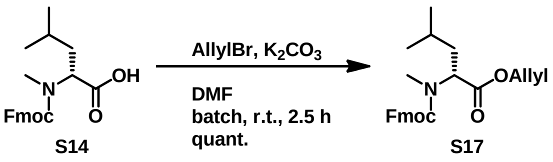

To a suspension of Fmoc-D-(*N*-Me)Leu-OH (**S14**) (1.84 g, 5.0 mmol, 1.0 equiv.) and K_2CO_3 (1.04 g, 7.5 mmol, 1.5 equiv.) in DMF (12.5 mL), allyl bromide (465 μL, 5.5 mmol, 1.1 equiv.) was added at room temperature under argon. After being stirred at the same temperature for 2.5 h, the reaction mixture was filtered through a pad of Celite, acidified with 1 M HCl at 0 °C and the aqueous layer was extracted with EtOAc. The combined organic layer was washed with 10% aqueous $Na_2S_2O_3$, saturated aqueous $NaHCO_3$ and brine, dried over $MgSO_4$, filtered and concentrated in vacuo. The residue was purified by column chromatography on silica gel (10% EtOAc in hexane) to give Fmoc-D-(*N*-Me)Leu-OAllyl (**S17**) (2.34 g, quant.) as a colorless oil.

^{1}H NMR (400 MHz, $CDCl_3$, major rotamer): δ 7.76 (m, 2H), 7.60 (m, 2H), 7.40 (m, 2H), 7.31 (m, 2H), 5.89 (m, 1H), 5.34-5.21 (m, 2H), 4.95 (dd, *J* =8.3, 7.8 Hz, 1H), 4.61 (m, 2H), 4.41 (m, 2H), 4.28 (dd, *J* = 6.8, 7.3 Hz, 1H), 2.87 (s, 3H), 1.73 (dd, *J* = 7.3, 7.8 Hz, 1H), 1.64-1.45 (m, 2H), 0.96 (d, *J* = 6.8 Hz, 3H), 0.94 (d, *J* = 6.4 Hz, 3H); ^{13}C NMR (100 MHz, $CDCl_3$, mixture of rotamers): δ 171.7, 171.3, 156.8, 156.3, 144.0, 143.9, 143.8, 141.3, 131.8, 131.7, 127.6, 127.0, 125.0, 124.9, 124.7, 119.9, 118.4, 67.6, 67.4, 65.5, 56.6, 56.5, 47.2, 37.6, 37.4, 30.3, 30.0, 24.8, 24.6, 23.2, 23.0, 21.3, 21.0; IR (neat): 2957, 1703, 1742, 1452, 1402, 1317, 1155, 988, 740 cm^{-1}; $[\alpha]^{23}_D$ = +16.0 (c 0.94, $CHCl_3$); HRMS (ESI-TOF): calcd for $[C_{25}H_{29}NO_4 + H]^+$ 408.2175, found

408.2175.

H-D-(*N*-Me)Leu-OAllyl (S18)

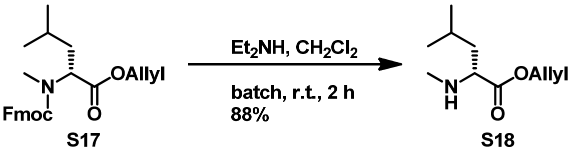

To a solution of Fmoc-D-(*N*-Me)Leu-OAllyl (**S17**) (204 mg, 0.500 mmol, 1.0 equiv.) in CH_2Cl_2 (1.0 mL), diethylamine (1.0 mL) was added at room temperature under argon. After being stirred at the same temperature for 2 h, the reaction mixture was concentrated in vacuo. The residue was purified by column chromatography on silica gel (3% MeOH in $CHCl_3$) to give H-D-(*N*-Me)Leu-OAllyl (**S18**) (82 mg, 0.443 mmol, 88%) as a colorless oil.

^{1}H NMR (400 MHz, $CDCl_3$): δ 5.93 (m, 1H), 5.36-5.23 (m, 2H), 4.63 (d, J = 5.4 Hz, 2H), 3.21 (dd, J = 7.3, 7.3 Hz, 1H), 2.36 (br, 3H), 1.73 (m, 1H), 1.51-1.44 (m, 3H), 0.93 (d, J = 6.8 Hz, 3H), 0.91 (d, J = 6.8 Hz, 3H); ^{13}C NMR (100 MHz, $CDCl_3$): δ 175.2, 132.0, 118.2, 64.9, 61.6, 42.5, 34.5, 24.7, 22.5, 22.2; IR (neat): 2959, 2872, 1737, 1171, 991 cm^{-1}; $[\alpha]^{23}_D$ = +4.6 (c 1.12, $CHCl_3$); HRMS (ESI-TOF): calcd for $[C_{10}H_{19}NO_2 + H]^+$ 186.1494, found 186.1495.

Fmoc-L-Val-D-(*N*-Me)Leu-OAllyl (S15)

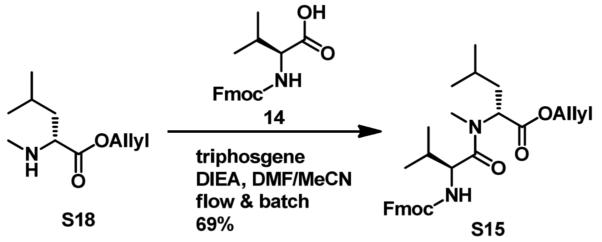

A solution of Fmoc-L-Val-OH (**14**) (0.35 M, 2.5 equiv.) and DIEA (0.42 M, 3.0 equiv.) in DMF (flow rate: 2.0 mL/min) and a solution of triphosgene (0.092 M, 0.4 equiv.) in MeCN (flow rate: 1.2 mL/min) were introduced to T-shape mixer 1 at 20 °C with the syringe pumps. The resultant mixture was passed through reaction tube 1 (inner diameter: 0.8 mm, length: 54 mm, volume: 27 μL, reaction time: 0.5 s) at the same temperature. Then, the resultant mixture and a solution of H-D-(*N*-Me)Leu-OAllyl (**S18**) (0.14 M, 1.0 equiv.) in MeCN (flow rate: 2.0 mL/min) were introduced to T-shape mixer 2 at 20 °C. The resultant mixture was passed through reaction tube 2 (inner diameter: 0.8 mm, length: 2224 mm, volume: 1118 μL, reaction time: 12.9 s) at the same temperature. After being eluted for 40 s to reach a steady state, the resultant mixture was poured into dry flask for 30 s at room temperature. After being stirred at the same temperature for 10 min, the reaction mixture was quenched with saturated aqueous

NH_4Cl. The aqueous layer was extracted twice with $CHCl_3$. The combined organic layer was washed with 1 M HCl, saturated aqueous $NaHCO_3$ and brine, dried over $MgSO_4$, filtered and concentrated in vacuo. The residue was purified by column chromatography on silica gel (2% MeOH in $CHCl_3$) to give Fmoc-L-Val-D-(*N*-Me)Leu-OAllyl (**S15**) (48 mg, 0.095 mmol, 69%, epimer <1%) as a colorless oil. Yield was determined by HPLC-UV analysis (conditions: DAICEL CHIRALPAK ID 4.6 mm×25 cm, 3% EtOH in hexane, flow rate 1 mL/min, detection wavelength 266 nm, retention time 40.20 min, epimer 33.83 min).

^{1}H NMR (400 MHz, $CDCl_3$, major rotamer): δ 7.75 (d, *J* = 7.3 Hz, 2H), 7.60 (d, *J* = 7.8 Hz, 2H), 7.39 (dd, *J* = 7.4, 7.8 Hz, 2H), 7.31 (dd, *J* = 7.3, 7.3 Hz, 2H), 5.86 (m, 1H), 5.64 (d, *J* = 9.3 Hz, 1H), 5.29-5.17 (m, 3H), 4.65 (dd, *J* = 4.9, 8.8 Hz, 1H), 4.58 (d, *J* = 5.9 Hz, 2H), 4.42-4.30 (m, 2H), 4.22 (dd, *J* = 6.8, 7.4 Hz, 1H), 3.03 (s, 3H), 2.01 (m, 1H), 1.77 (m, 2H), 1.45 (m ,1H), 1.00 (d, *J* = 6.8 Hz, 3H), 0.96 (d, *J* = 6.8 Hz, 3H), 0.93-0.89 (m, 6H); ^{13}C NMR (100 MHz, $CDCl_3$, mixture of rotamers): δ 172.8, 171.0, 156.4, 143.9, 143.8, 141.2, 131.7, 127.6, 127.0, 125.1, 119.9, 118.5, 67.0, 65.7, 55.9, 54.9, 47.2, 37.3, 31.6, 31.4, 24.9, 23.3, 21.1, 19.7, 16.8; IR (neat): 3313, 2961, 1738, 1645, 1528, 1451, 1410, 1238, 1030, 759, 741 cm^{-1}; $[\alpha]^{23}_D$ = +19.9 (c 0.96, $CHCl_3$); HRMS (ESI-TOF): calcd for $[C_{30}H_{38}N_2O_5 + H]^+$ 507.2859, found 507.2846.

Fmoc-L-Val-D-(*N*-Me)Leu-OH (S16)

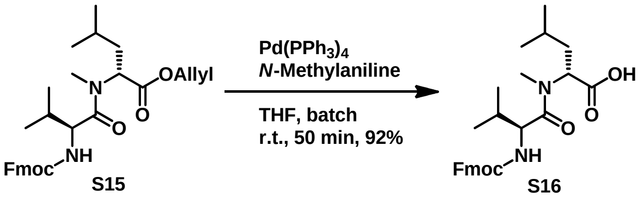

To a solution of Fmoc-L-Val-D-(*N*-Me)Leu-OAllyl (**S15**) (494 mg, 0.987 mmol, 1.0 equiv.) and *N*-methylaniline (267 μL, 2.47 mmol, 2.5 equiv.) in THF (4.9 mL), tetrakis(triphenylphosphine)palladium (114 mg, 0.099 mmol, 0.1 equiv.) was added at room temperature under argon. After being stirred at the same temperature for 50 min, the reaction mixture was concentrated in vacuo. The residue was purified by column chromatography on silica gel (5% MeOH and 0.1% formic acid in $CHCl_3$) to give Fmoc-L-Val-D-(*N*-Me)Leu-OH (**S16**) (424 mg, 0.909 mmol, 92%) contaminated with a trace amount of triphenylphosphine oxide as a white solid.

^{1}H NMR (400 MHz, $CDCl_3$, major rotamer): δ 7.73 (d, *J* = 7.3 Hz, 2H), 7.59 (m, 2H), 7.37 (m, 2H), 7.29 (m, 2H), 5.82 (d, *J* = 9.3 Hz, 1H), 5.22 (br, 1H), 4.62 (dd, *J* = 5.4, 8.8 Hz, 1H), 4.38-4.26 (m, 2H), 4.19 (t, *J* = 7.3 Hz, 1H), 3.02 (s, 3H), 2.00 (m, 1H), 1.76 (m, 2H), 1.42 (m, 1H), 0.98 (d, *J* = 6.8 Hz, 3H), 0.93 (d, *J* = 6.8 Hz, 3H), 0.90 (brd, *J* = 6.4 Hz, 6H); ^{13}C NMR (100 MHz, $CDCl_3$, mixture of rotamers): δ 174.9, 173.3, 156.6, 143.9, 143.8, 141.2, 127.6,

127.0, 125.2, 119.9, 67.1, 56.1, 55.0, 47.1, 37.1, 31.8, 31.3, 24.9, 23.3, 21.1, 19.6, 16.9; IR (neat): 3306, 2961, 1717, 1646, 1611, 1451, 1247, 757, 741 cm^{-1}; $[\alpha]^{23}_{D}$ = +21.3 (c 0.69, $CHCl_3$); mp 75-78 °C; HRMS (ESI-TOF): calcd for $[C_{27}H_{34}N_2O_5 + H]^+$ 467.2546, found 467.2548.

Fmoc-L-Val-D-(*N*-Me)Leu-Sar-L-Val-OAllyl (17)

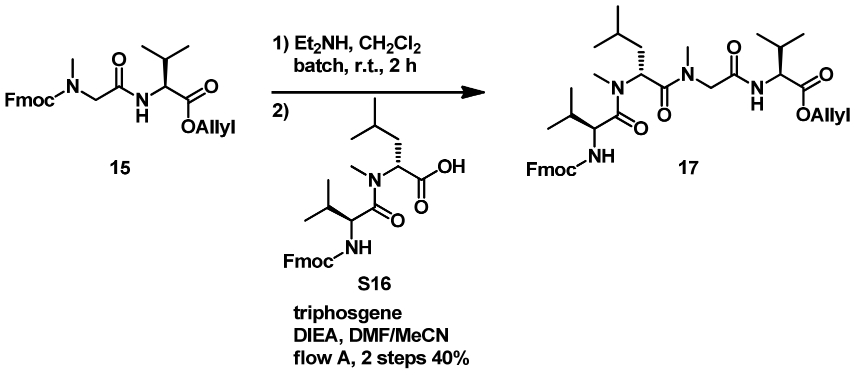

To a solution of Fmoc-Sar-L-Val-OAllyl (**15**) (151 mg, 0.336 mmol, 1.0 equiv.) in CH_2Cl_2 (0.7 mL), diethylamine (0.7 mL) was added at room temperature under argon. After being stirred at the same temperature for 2 h, the reaction mixture was concentrated in vacuo. The residue was azeotroped twice with toluene and used for the next reaction without further purification.

A solution of Fmoc-L-Val-D-(*N*-Me)Leu-OH (**S16**) (0.35 M, 2.5 equiv.) and DIEA (0.42 M, 3.0 equiv.) in DMF (flow rate: 2.0 mL/min) and a solution of triphosgene (0.092 M, 0.4 equiv.) in MeCN (flow rate: 1.2 mL/min) were introduced to T-shape mixer 1 at 20 °C with the syringe pumps. The resultant mixture was passed through reaction tube 1 (inner diameter: 0.8 mm, length: 54 mm, volume: 27 μL, reaction time: 0.5 s) at the same temperature. Then, the resultant mixture and a solution of crude amine (0.14 M, 1.0 equiv.) in MeCN (flow rate: 2.0 mL/min) were introduced to T-shape mixer 2 at 20 °C. The resultant mixture was passed through reaction tube 2 (inner diameter: 0.8 mm, length: 742 mm, volume: 373 μL, reaction time: 4.3 s) at the same temperature. After being eluted for 40 s to reach a steady state, the resultant mixture was poured into saturated aqueous NH_4Cl, brine and $CHCl_3$ suspension for 30 s at room temperature. The aqueous layer was extracted twice with $CHCl_3$. The combined organic layer was washed with 1 M HCl, saturated aqueous $NaHCO_3$ and brine, dried over $MgSO_4$, filtered and concentrated in vacuo. The residue was purified by column chromatography on silica gel (2% MeOH in $CHCl_3$) and GPC to give Fmoc-L-Val-D-(*N*-Me)Leu-Sar-L-Val-OAllyl (**17**) (38 mg, 0.056 mmol, 2 steps 40%, epimer <1%) as a colorless oil. Yield was determined by HPLC-UV analysis (conditions: DAICEL CHIRALPAK ID 4.6 mm×25 cm, 10% EtOH in hexane, flow rate 1 mL/min, detection wavelength 266 nm, retention time 25.15 min, epimer 35.85 min).

10.3. Synthesis of epimers

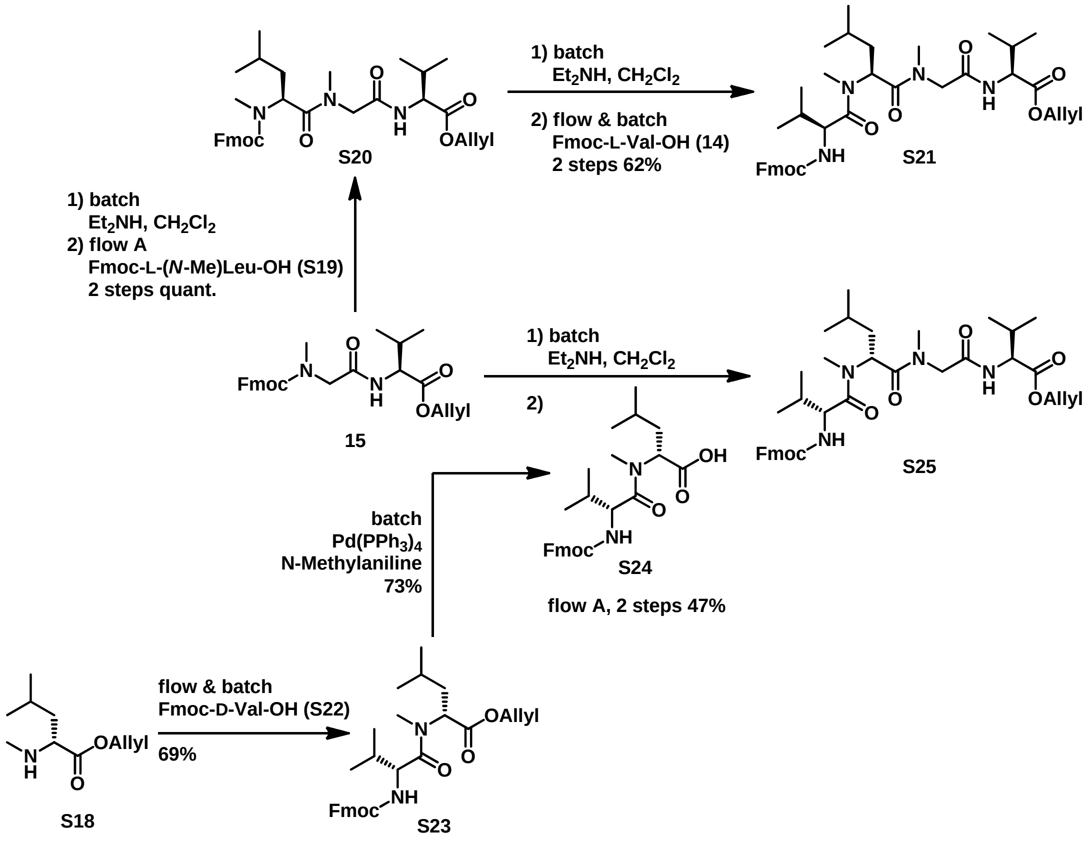

Scheme S-2

Fmoc-L-(*N*-Me)Leu-Sar-L-Val-OAllyl (S20)

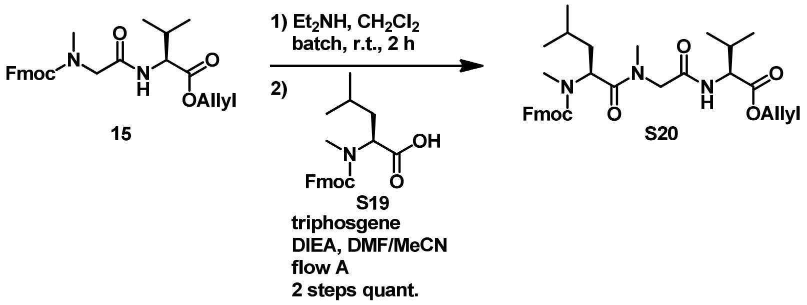

To a solution of Fmoc-Sar-L-Val-OAllyl (**15**) (431 mg, 0.957 mmol, 1.0 equiv.) in CH_2Cl_2 (1.9 mL), diethylamine (1.9 mL) was added at room temperature under argon. After being stirred at the same temperature for 2 h, the reaction mixture was concentrated in vacuo. The residue was azeotroped twice with toluene and used for the next reaction without further purification.

A solution of Fmoc-L-(*N*-Me)Leu-OH (**S19**) (0.35 M, 2.5 equiv.) and DIEA (0.42 M, 3.0 equiv.) in DMF (flow rate: 2.0 mL/min) and a solution of triphosgene (0.092 M, 0.4 equiv.) in

MeCN (flow rate: 1.2 mL/min) were introduced to T-shape mixer 1 at 20 °C with the syringe pumps. The resultant mixture was passed through reaction tube 1 (inner diameter: 0.8 mm, length: 54 mm, volume: 27 μL, reaction time: 0.5 s) at the same temperature. Then, the resultant mixture and a solution of crude amine (0.14 M, 1.0 equiv.) in MeCN (flow rate: 2.0 mL/min) were introduced to T-shape mixer 2 at 20 °C. The resultant mixture was passed through reaction tube 2 (inner diameter: 0.8 mm, length: 742 mm, volume: 373 μL, reaction time: 4.3 s) at the same temperature. After being eluted for 40 s to reach a steady state, the resultant mixture was poured into saturated aqueous NH_4Cl, brine and $CHCl_3$ suspension for 164 s at room temperature. The aqueous layer was extracted twice with $CHCl_3$. The combined organic layer was washed with 1 M HCl, saturated aqueous $NaHCO_3$ and brine, dried over $MgSO_4$, filtered and concentrated in vacuo. The residue was purified by column chromatography on silica gel (2% MeOH in $CHCl_3$) to give Fmoc-L-(*N*-Me)Leu-Sar-L-Val-OAllyl (**S20**) (445 mg, 2 steps quant.) as a colorless oil.

^{1}H NMR (400 MHz, $CDCl_3$, major rotamer): δ 7.75 (d, *J* = 7.8 Hz, 2H), 7.58 (d, *J* = 7.3 Hz, 2H), 7.39 (dd, *J* = 7.3, 7.8 Hz, 2H), 7.30 (dd, *J* = 7.3, 7.8 Hz, 2H), 6.71 (d, *J* = 8.8 Hz, 1H), 5.85 (m, 1H), 5.31-5.19 (m, 2H), 5.14 (dd, *J* = 5.9, 8.8 Hz, 1H), 4.56 (m, 2H), 4.51 (dd, *J* = 4.9, 8.8 Hz, 1H), 4.45 (m, 2H), 4.24 (m, 1H), 4.21 (d, *J* = 15.1 Hz, 1H), 3.87 (d, *J* = 15.6 Hz, 1H), 3.14 (s, 3H), 2.89 (s, 3H), 2.15 (m, 1H), 1.72-1.40 (m, 3H), 0.96-0.86 (m, 12H); ^{13}C NMR (100 MHz, $CDCl_3$, mixture of rotamers): δ 172.1, 171.1, 168.4, 156.5, 143.7, 141.2, 131.5, 128.1, 127.6, 127.2, 127.0, 126.9, 124.9, 124.1, 119.9, 118.8, 118.7, 67.5, 65.8, 65.6, 57.0, 56.9, 52.8, 52.5, 52.3, 47.2, 37.7, 36.4, 35.3, 30.9, 29.4, 24.6, 24.3, 22.9, 22.3, 22.1, 21.9, 18.8, 17.6, 17.4; IR (neat): 3327, 2960, 1742, 1695, 1533, 1452, 1397, 1310, 1157, 759, 741 cm^{-1}; $[\alpha]^{22}_D$ = -43.6 (c 0.86, $CHCl_3$); HRMS (ESI-TOF): calcd for $[C_{33}H_{43}N_3O_6 + H]^+$ 578.3230, found 578.3233.

Fmoc-L-Val-L-(*N*-Me)Leu-Sar-L-Val-OAllyl (S21)

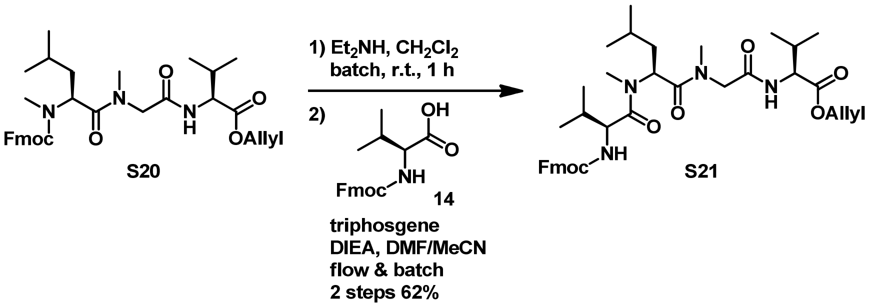

To a solution of Fmoc-L-(*N*-Me)Leu-Sar-L-Val-OAllyl (**S20**) (289 mg, 0.500 mmol, 1.0 equiv.) in CH_2Cl_2 (1.0 mL), diethylamine (1.0 mL) was added at room temperature under argon. After being stirred at the same temperature for 1 h, the reaction mixture was concentrated in vacuo. The residue was azeotroped twice with toluene and used for the next reaction without

further purification.

A solution of Fmoc-L-Val-OH (**S14**) (0.35 M, 2.5 equiv.) and DIEA (0.42 M, 3.0 equiv.) in DMF (flow rate: 2.0 mL/min) and a solution of triphosgene (0.092 M, 0.4 equiv.) in MeCN (flow rate: 1.2 mL/min) were introduced to T-shape mixer 1 at 20 °C with the syringe pumps. The resultant mixture was passed through reaction tube 1 (inner diameter: 0.8 mm, length: 54 mm, volume: 27 μL, reaction time: 0.5 s) at the same temperature. Then, the resultant mixture and a solution of crude amine (0.14 M, 1.0 equiv.) in MeCN (flow rate: 2.0 mL/min) were introduced to T-shape mixer 2 at 20 °C. The resultant mixture was passed through reaction tube 2 (inner diameter: 0.8 mm, length: 2224 mm, volume: 1118 μL, reaction time: 12.9 s) at the same temperature. After being eluted for 40 s to reach a steady state, the resultant mixture was poured into dry flask for 68 s at room temperature. After being stirred at the same temperature for 10 min, the reaction mixture was quenched with saturated aqueous NH_4Cl. The aqueous layer was extracted twice with $CHCl_3$. The combined organic layer was washed with 1 M HCl, saturated aqueous $NaHCO_3$ and brine, dried over $MgSO_4$, filtered and concentrated in vacuo. The residue was purified by column chromatography on silica gel (2% MeOH in $CHCl_3$) to give Fmoc-L-Val-L-(*N*-Me)Leu-Sar-L-Val-OAllyl (**S21**) (134 mg, 0.198 mmol, 2 steps 62%) as a white solid.

^{1}H NMR (400 MHz, $CDCl_3$, major rotamer): δ 7.75 (d, *J* = 7.8 Hz, 2H), 7.60 (d, *J* = 7.3 Hz, 2H), 7.39 (dd, *J* = 7.4, 7.3 Hz, 2H), 7.30 (dd, *J* = 7.8, 7.4 Hz, 2H), 6.70 (d, *J* = 8.3 Hz, 1H), 5.89 (m, 1H), 5.63 (d, *J* = 9.3 Hz, 1H), 5.45 (dd, *J* = 7.3, 7.3 Hz, 1H), 5.35-5.24 (m, 2H), 4.63-4.52 (m, 4H), 4.38, (m, 2H), 4.35 (d, *J* = 15.6 Hz, 1H), 4.21 (t, *J* = 7.3 Hz, 1H), 3.74 (d, *J* = 15.6 Hz, 1H), 3.12 (s, 3H), 3.09 (s, 3H), 2.19 (m, 1H), 2.08 (m, 1H), 1.71 (m, 1H), 1.60 (m, 1H), 1.48 (m, 1H), 0.99-0.89 (m, 18H); ^{13}C NMR (100 MHz, $CDCl_3$, mixture of rotamers): δ 172.3, 171.5, 171.3, 168.4, 156.4, 143.8, 143.7, 141.2, 131.5, 128.9, 128.1, 127.6, 127.0, 125.0, 119.9, 118.9, 67.0, 65.7, 57.0, 56.0, 52.5, 51.5, 47.1, 37.6, 36.3, 30.9, 24.7, 22.7, 22.3, 19.7, 18.9, 17.6, 17.0; IR (neat): 3314, 2963, 1718, 1694, 1636, 1531, 1451, 1409, 1238, 1198, 1149, 1109, 1029, 757, 742 cm^{-1}; $[\alpha]^{22}_D$ = -42.9 (c 1.02, $CHCl_3$); mp 54-56 °C; HRMS (ESI-TOF): calcd for $[C_{38}H_{52}N_4O_7 + H]^+$ 677.3914, found 677.3916.

Fmoc-D-Val-D-(*N*-Me)Leu-OAllyl (S23)

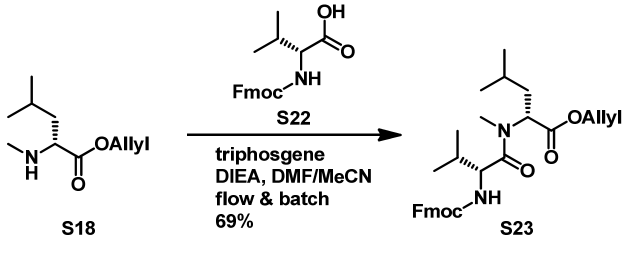

A solution of Fmoc-D-Val-OH (**S22**) (0.35 M, 2.5 equiv.) and DIEA (0.42 M, 3.0 equiv.) in DMF (flow rate: 2.0 mL/min) and a solution of triphosgene (0.092 M, 0.4 equiv.) in MeCN (flow rate: 1.2 mL/min) were introduced to T-shape mixer 1 at 20 °C with the syringe pumps. The resultant mixture was passed through reaction tube 1 (inner diameter: 0.8 mm, length: 54 mm, volume: 27 μL, reaction time: 0.5 s) at the same temperature. Then, the resultant mixture and a solution of H-D-(*N*-Me)Leu-OAllyl (**S18**) (0.14 M, 1.0 equiv.) in MeCN (flow rate: 2.0 mL/min) were introduced to T-shape mixer 2 at 20 °C. The resultant mixture was passed through reaction tube 2 (inner diameter: 0.8 mm, length: 2224 mm, volume: 1118 μL, reaction time: 12.9 s) at the same temperature. After being eluted for 40 s to reach a steady state, the resultant mixture was poured into dry flask for 30 s at room temperature. After being stirred at the same temperature for 10 min, the reaction mixture was quenched with saturated aqueous NH_4Cl. The aqueous layer was extracted twice with $CHCl_3$. The combined organic layer was washed with 1 M HCl, saturated aqueous $NaHCO_3$ and brine, dried over $MgSO_4$, filtered and concentrated in vacuo. The residue was purified by column chromatography on silica gel (2% MeOH in $CHCl_3$) to give Fmoc-D-Val-D-(*N*-Me)Leu-OAllyl (**S23**) (48 mg, 0.095 mmol, 69%) as a colorless oil.

^{1}H NMR (400 MHz, $CDCl_3$, major rotamer): δ 7.76 (d, *J* = 7.3 Hz, 2H), 7.60 (d, *J* = 7.3 Hz, 2H), 7.40 (dd, *J* = 7.3, 7.4 Hz, 2H), 7.31 (dd, *J* = 7.3, 7.8 Hz, 2H), 5.88 (m, 1H), 5.56 (d, *J* = 9.2 Hz, 1H), 5.37 (dd, *J* = 3.9, 9.8 Hz, 1H), 5.33-5.23 (m, 2H), 4.59 (brd, *J* = 4.9 Hz, 2H), 4.56 (m, 1H), 4.36 (m, 2H), 4.22 (t, *J* =6.8 Hz, 1H), 3.02 (s, 3H), 2.08 (m, 1H), 1.79-1.67 (m, 2H), 1.48 (m, 1H), 1.06-0.90 (m, 12H); ^{13}C NMR (100 MHz, $CDCl_3$, mixture of rotamers): δ 172.8, 171.2, 156.4, 143.9, 143.8, 141.3, 131.6, 127.7, 127.0, 125.1, 119.9, 118.8, 67.0, 65.8, 55.8, 54.7, 47.2, 36.9, 31.3, 31.2, 24.8, 23.2, 21.4, 19.5, 17.3; IR (neat): 3312, 2960, 2873, 1738, 1645, 1527, 1451, 1411, 1262, 1235, 1030, 741 cm^{-1}; $[\alpha]^{22}_D$ = +28.1 (c 0.95, $CHCl_3$); HRMS (ESI-TOF): calcd for $[C_{30}H_{38}N_2O_5 + H]^+$ 507.2859, found 507.2856.

Fmoc-D-Val-D-(*N*-Me)Leu-OH (S24)

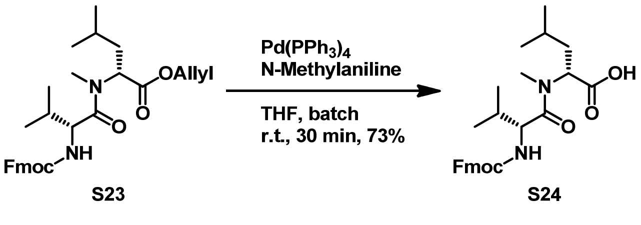

To a solution of Fmoc-D-Val-D-(*N*-Me)Leu-OAllyl (**S23**) (695 mg, 1.37 mmol, 1.0 equiv.) and *N*-methylaniline (371 μL, 3.43 mmol, 2.5 equiv.) in THF (6.9 mL), tetrakis(triphenylphosphine)palladium (162 mg, 0.14 mmol, 0.1 equiv.) was added at room temperature under argon. After being stirred at the same temperature for 30 min, the reaction

mixture was concentrated in vacuo. The residue was purified by column chromatography on silica gel (5% MeOH and 0.1% formic acid in $CHCl_3$) to give Fmoc-D-Val-D-(*N*-Me)Leu-OH (**S24**) (465 mg, 0.997 mmol, 73%) contaminated with a trace amount of triphenylphosphine oxide as a white solid.

^{1}H NMR (400 MHz, $CDCl_3$, major rotamer): δ 7.75 (d, *J* = 7.3 Hz, 2H), 7.60 (d, *J* = 7.3 Hz, 2H), 7.39 (dd, *J* = 7.3, 7.8 Hz, 2H), 7.30 (dd, *J* = 7.8, 7.3 Hz, 2H), 5.87 (d, *J* = 9.2 Hz, 1H), 5.33 (dd, *J* = 4.4, 10.2 Hz, 1H), 4.54 (dd, *J* = 7.3, 9.3 Hz, 1H), 4.33 (d, *J* = 7.3 Hz, 2H), 4.21 (t, *J* = 7.3 Hz, 1H), 3.04 (s, 3H), 2.07 (m, 1H), 1.75 (m, 2H), 1.48 (m, 1H), 0.99 (d, *J* = 6.8 Hz, 3H), 0.95 (d, *J* = 6.8 Hz, 3H), 0.89 (d, *J* = 6.8 Hz, 3H), 0.86 (d, *J* = 6.4 Hz, 3H); ^{13}C NMR (100 MHz, $CDCl_3$, major rotamer): δ 175.3, 173.7, 156.6, 143.9, 143.8, 127.6, 127.0, 125.1, 119.9, 67.1, 56.1, 54.7, 47.1, 36.8, 31.5, 31.0, 24.8, 23.1, 21.3, 19.3, 17.6; IR (neat): 3300, 2961, 1715, 1646, 1607, 1532, 1451, 1413, 1263, 1235, 1121, 1030, 758, 741 cm^{-1}; $[\alpha]^{23}_D$ = +21.9 (c 1.08, $CHCl_3$); mp 75-78 °C; HRMS (ESI-TOF): calcd for $[C_{27}H_{34}N_2O_5 + H]^+$ 467.2546, found 467.2547.

Fmoc-D-Val-D-(*N*-Me)Leu-Sar-L-Val-OAllyl (S25)

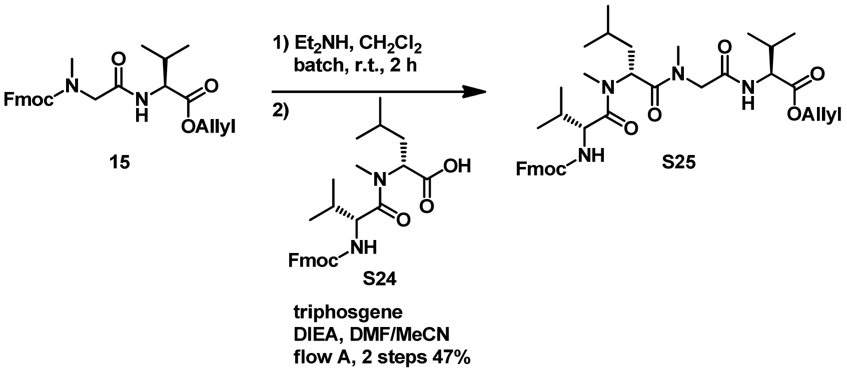

To a solution of Fmoc-Sar-L-Val-OAllyl (**15**) (151 mg, 0.336 mmol, 1.0 equiv.) in CH_2Cl_2 (0.7 mL), diethylamine (0.7 mL) was added at room temperature under argon. After being stirred at the same temperature for 2 h, the reaction mixture was concentrated in vacuo. The residue was azeotroped twice with toluene and used for the next reaction without further purification.

A solution of Fmoc-D-Val-D-(*N*-Me)Leu-OH (**S24**) (0.35 M, 2.5 equiv.) and DIEA (0.42 M, 3.0 equiv.) in DMF (flow rate: 2.0 mL/min) and a solution of triphosgene (0.092 M, 0.4 equiv.) in MeCN (flow rate: 1.2 mL/min) were introduced to T-shape mixer 1 at 20 °C with the syringe pumps. The resultant mixture was passed through reaction tube 1 (inner diameter: 0.8 mm, length: 54 mm, volume: 27 μL, reaction time: 0.5 s) at the same temperature. Then, the resultant mixture and a solution of crude amine (0.14 M, 1.0 equiv.) in MeCN (flow rate: 2.0 mL/min) were introduced to T-shape mixer 2 at 20 °C. The resultant mixture was passed through reaction tube 2 (inner diameter: 0.8 mm, length: 742 mm, volume: 373 μL, reaction time: 4.3 s) at the

same temperature. After being eluted for 40 s to reach a steady state, the resultant mixture was poured into saturated aqueous NH_4Cl, brine and $CHCl_3$ suspension for 30 s at room temperature. The aqueous layer was extracted twice with $CHCl_3$. The combined organic layer was washed with 1 M HCl, saturated aqueous $NaHCO_3$ and brine, dried over $MgSO_4$, filtered and concentrated in vacuo. The residue was purified by column chromatography on silica gel (2% MeOH in $CHCl_3$) and GPC to give Fmoc-D-Val-D-(*N*-Me)Leu-Sar-L-Val-OAllyl (**S25**) (45 mg, 0.066 mmol, 2 steps 47%) as a colorless oil.

^{1}H NMR (400 MHz, $CDCl_3$, mixture of rotamers): δ 7.75 (d, *J* = 7.8 Hz, 2H), 7.59 (m, 2H), 7.39 (dd, *J* = 7.8, 7.3 Hz, 2H), 7.30 (dd, *J* = 7.3, 7.3 Hz, 2H), 6.63-6.55 (m, 1H), 5.88 (m, 1H), 5.58 (m, 1H), 5.50 (dd, *J* = 6.4, 8.8 Hz, 1H), 5.35-5.20 (m, 2H), 4.64-4.21 (m, 7H), 4.09 (m, 1H), 3.95 (m, 1H), 3.15-3.07 (m, 6H), 2.17 (m, 1H), 2.02 (m, 1H), 1.65 (m, 2H), 1.47 (m, 1H), 1.00-0.85 (m, 18H); ^{13}C NMR (100 MHz, $CDCl_3$, mixture of rotamers): δ 172.4, 171.8, 171.2, 168.3, 156.4, 156.2, 143.8, 141.3, 131.6, 131.5, 127.6, 127.0, 125.1, 119.9, 119.0, 118.8, 67.0, 66.9, 65.7, 57.1, 56.0, 52.6, 52.3, 51.2, 50.9, 47.2, 37.8, 37.5, 36.6, 31.3, 31.1, 30.6, 24.8, 23.0, 22.8, 22.3, 21.7, 19.8, 19.6, 18.9, 17.6, 17.0, 16.8; IR (neat): 3320, 2963, 1719, 1694, 1636, 1532, 1451, 1409, 1236, 1198, 758, 742 cm^{-1}; $[\alpha]^{23}_D$ = +6.9 (c 1.08, $CHCl_3$); HRMS (ESI-TOF): calcd for $[C_{38}H_{52}N_4O_7 + H]^+$ 677.3914, found 677.3913.

11. HPLC charts

Boc-L-Ser(*O*-Bn)-L-Phe-OAllyl (3)

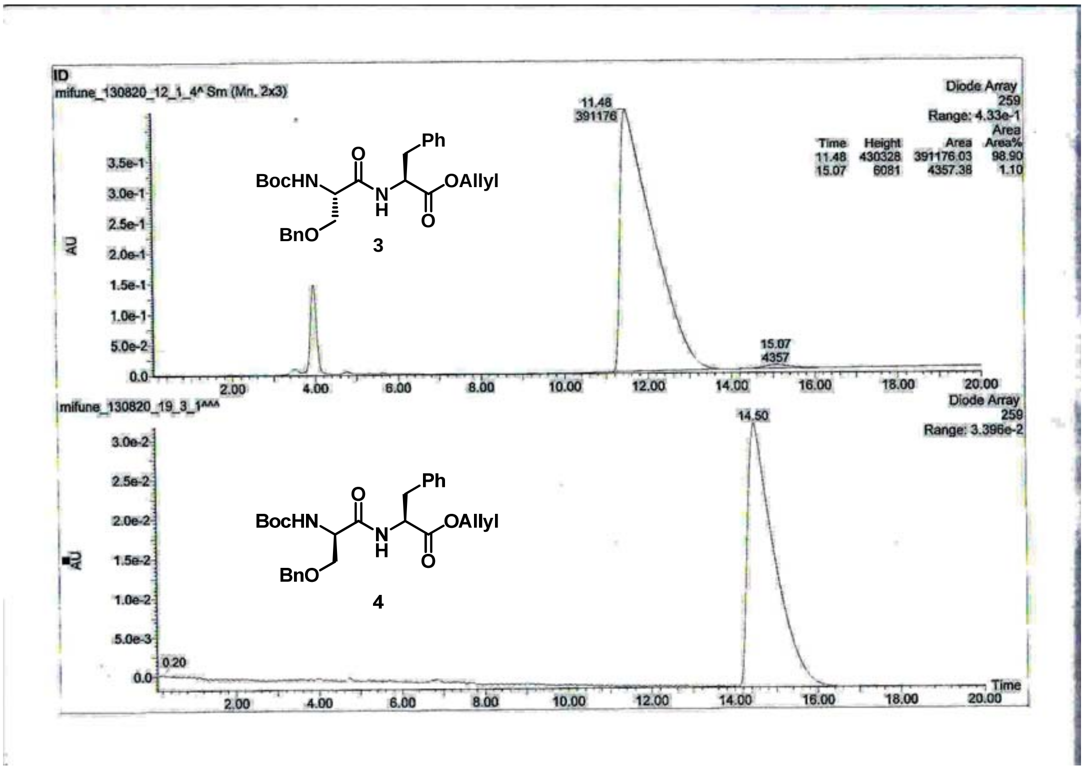

Boc-L-Tyr(*O*-Bn)-L-Phe-OAllyl (5)

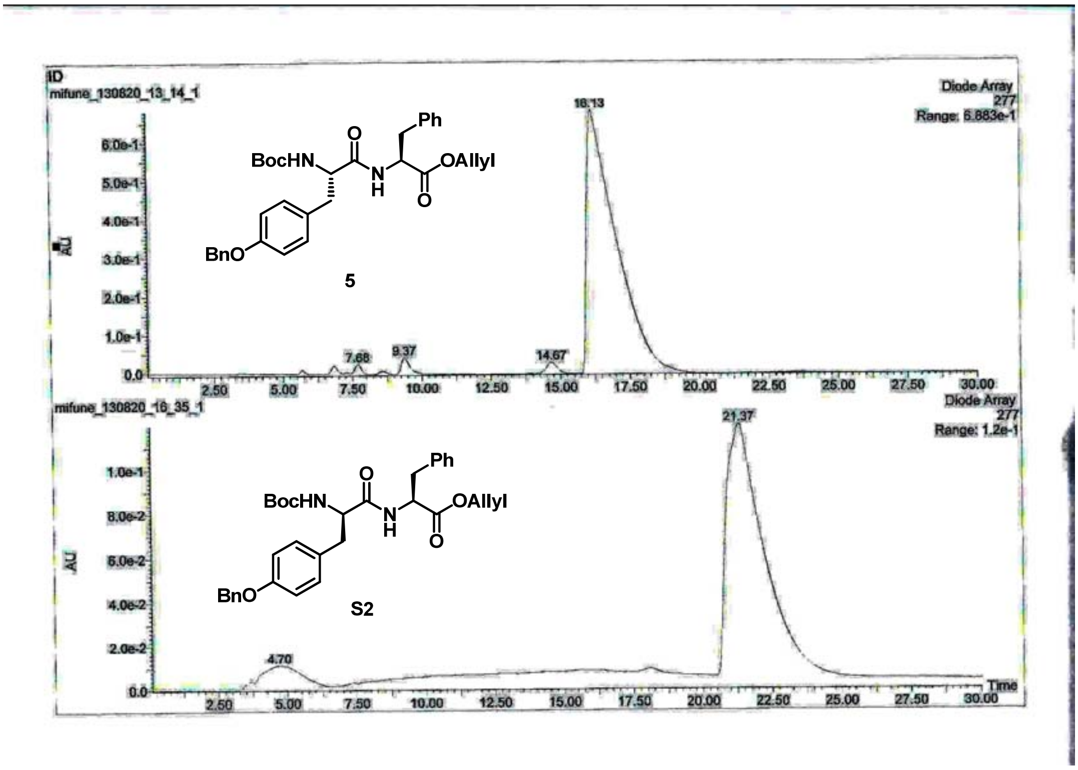

Fmoc-L-His(*N*-Trt)-L-Phe-OAllyl (6)

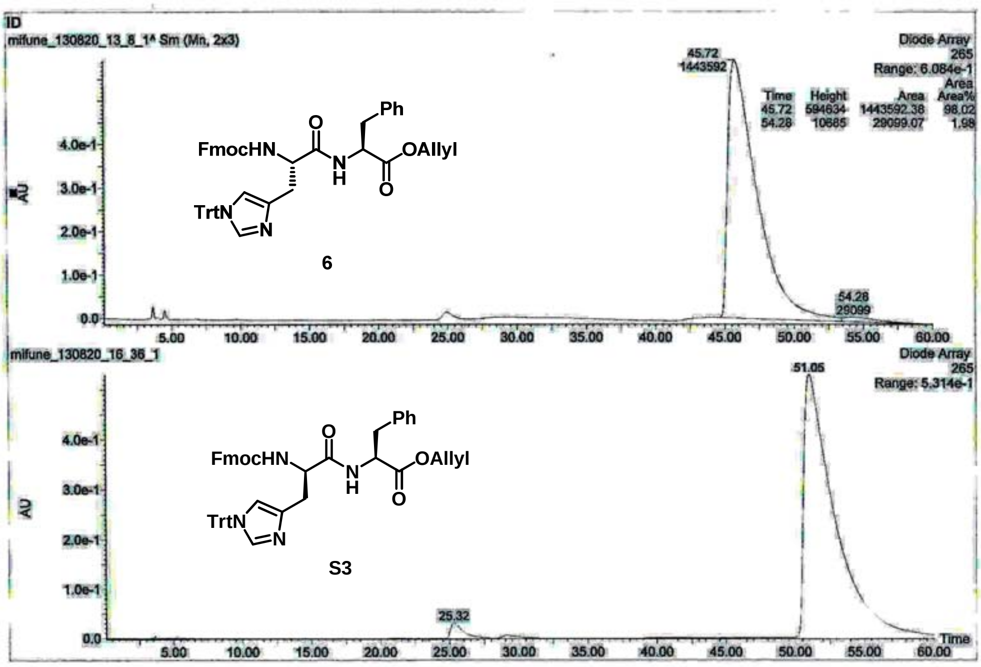

Fmoc-L-Cys(*S*-Trt)-L-Phe-OAllyl (7)

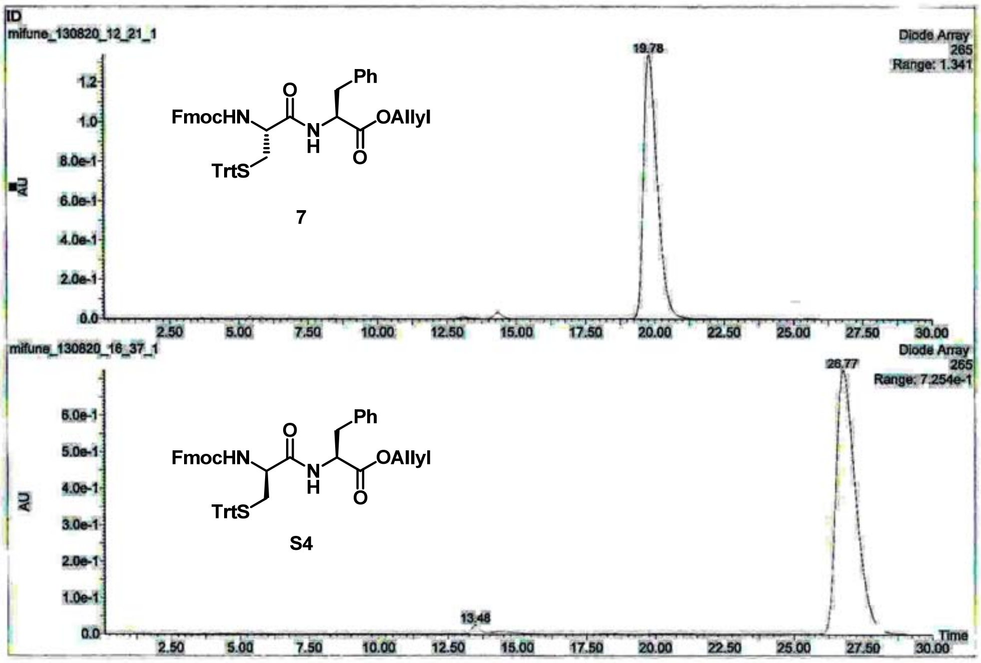

Boc-D-Phg-L-Phe-OAllyl (8)

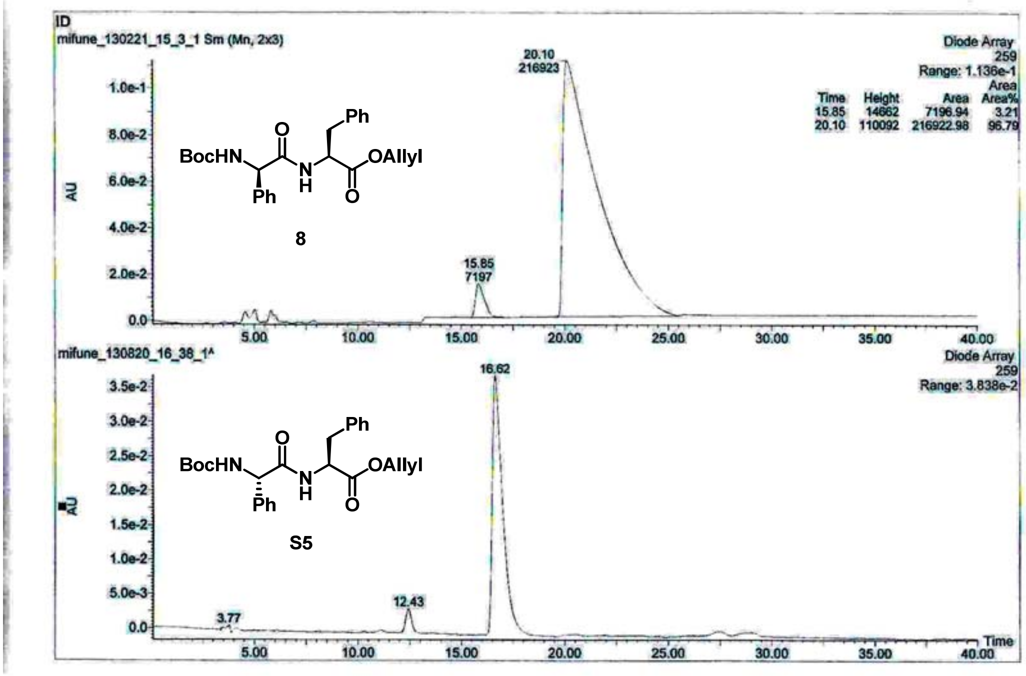

Boc-L-Ser(*O*-Bn)-L-Ile-OAllyl (9)

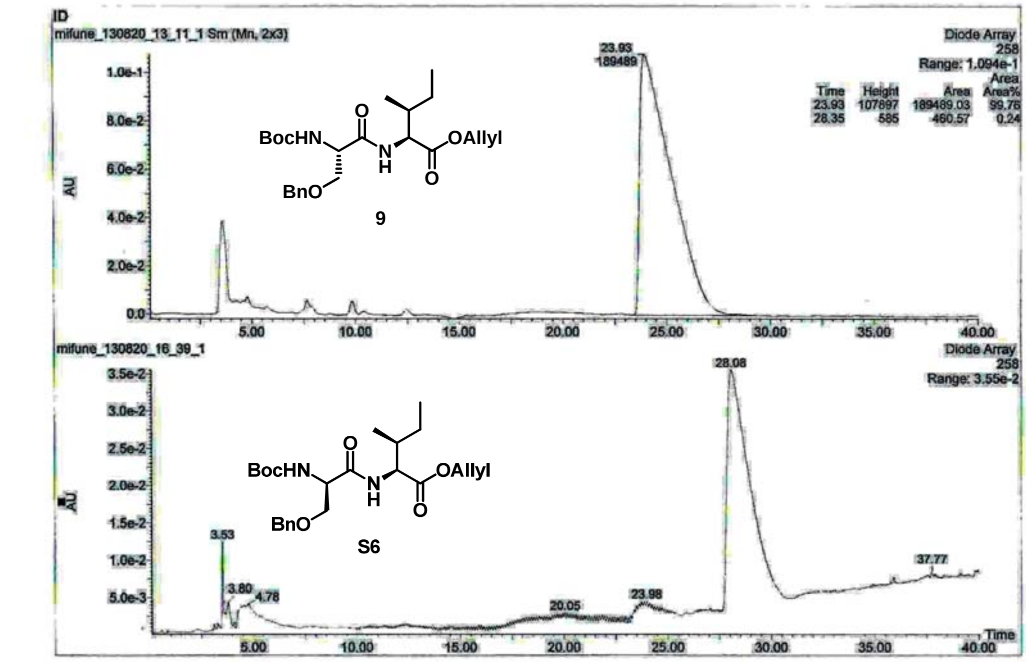

Boc-L-Ser(*O*-Bn)-L-Pro-OAllyl (10)

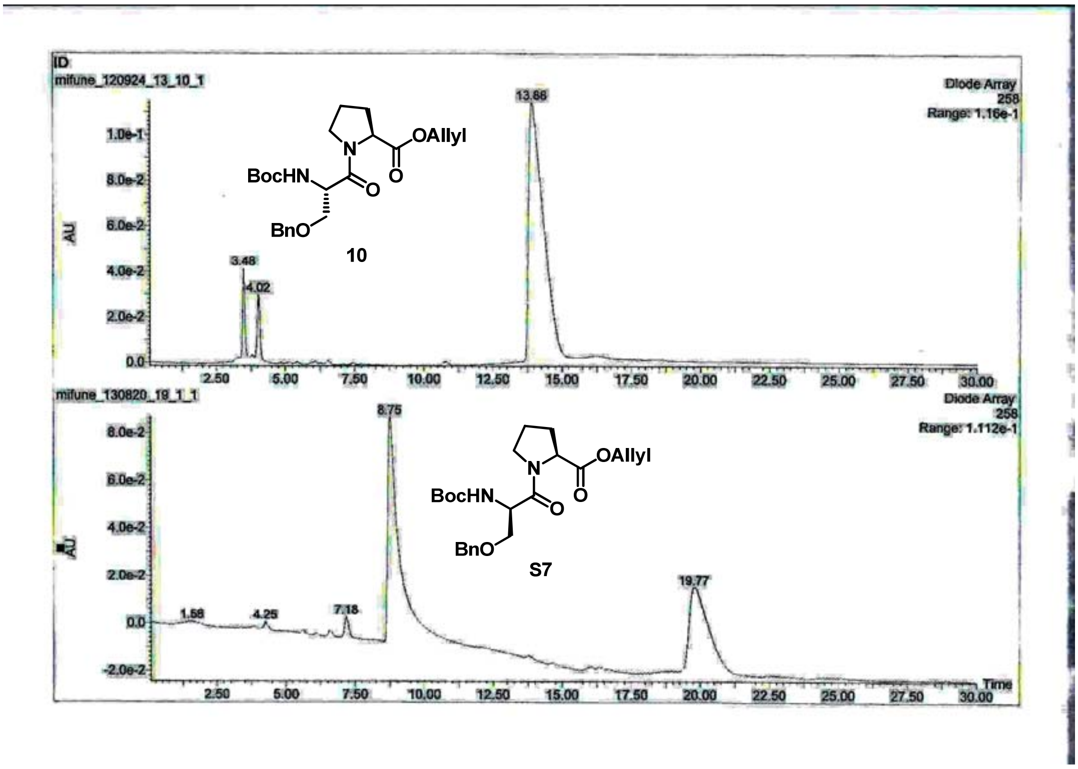

Boc-L-Ser(*O*-Bn)-Sar-OAllyl (11)

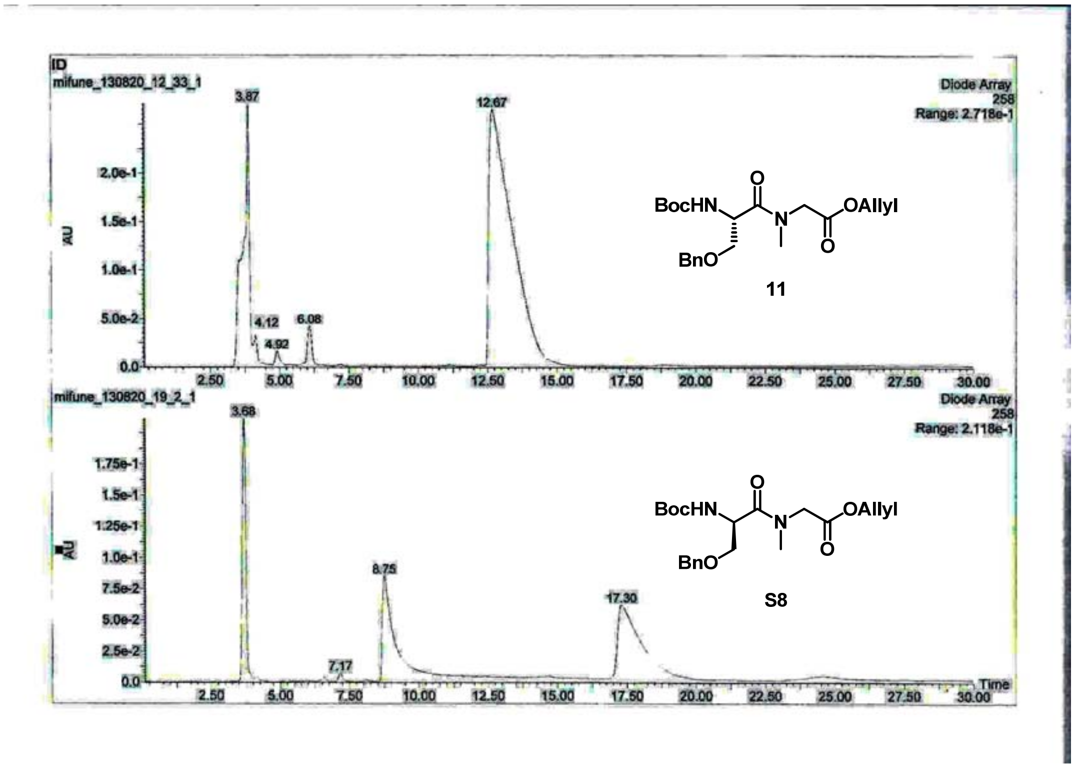

L-Lac-L-Phe-OAllyl (12)

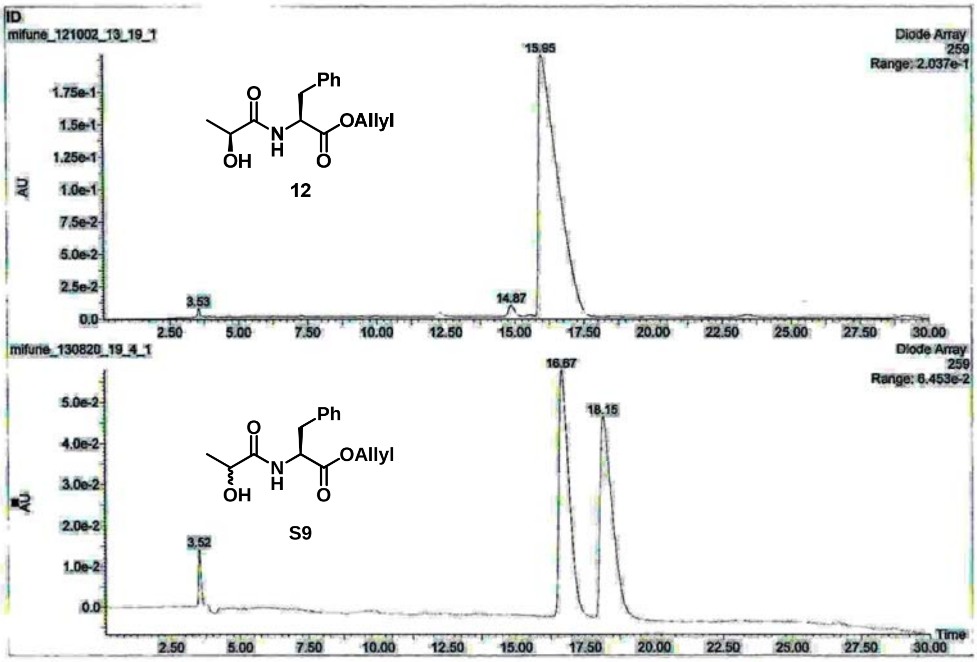

Fmoc-D-(*N*-Me)Leu-Sar-L-Val-OAllyl (16)

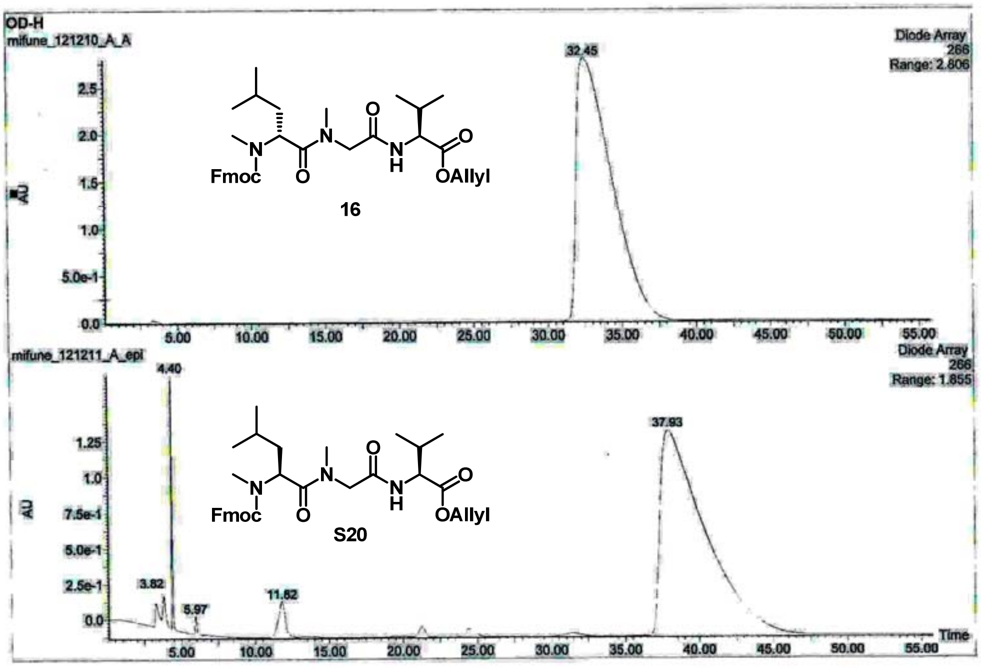

Fmoc-L-Val-D-(*N*-Me)Leu-Sar-L-Val-OAllyl (17) (Linear route)

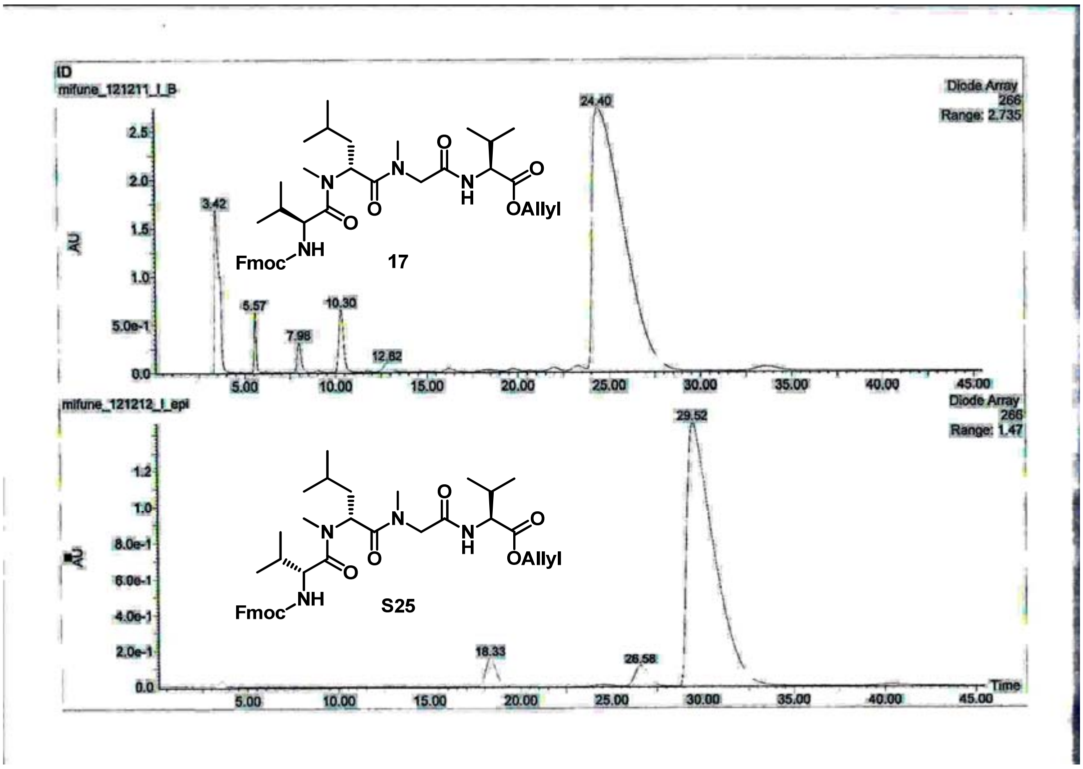

Fmoc-L-Val-D-(*N*-Me)Leu-OAllyl (S15)

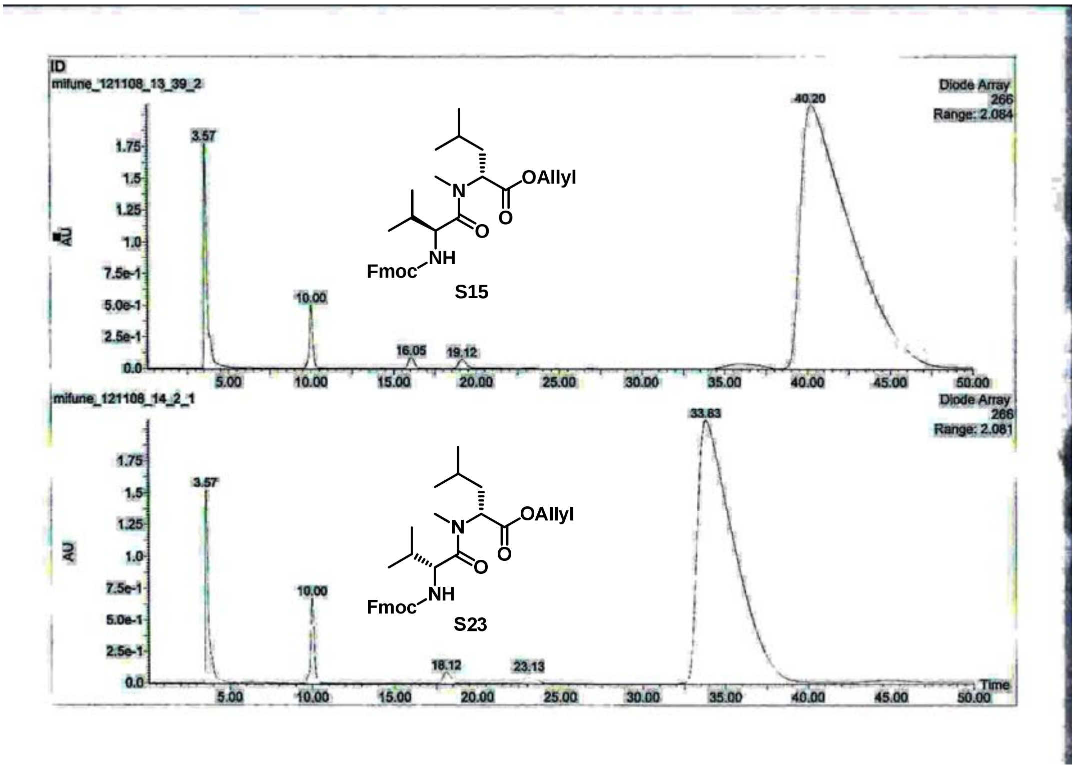

Fmoc-L-Val-D-(*N*-Me)Leu-Sar-L-Val-OAllyl (17) (Convergent route)

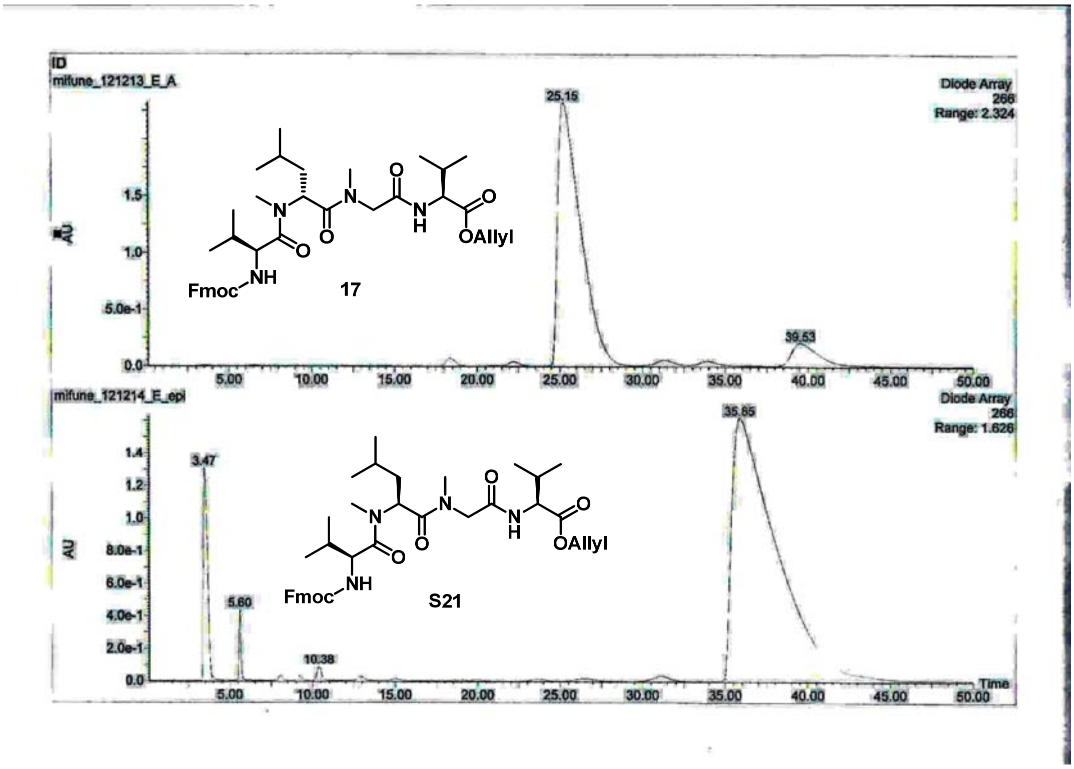

12. NMR spectra

Boc-L-Ser(*O*-Bn)-L-Phe-OAllyl (3)

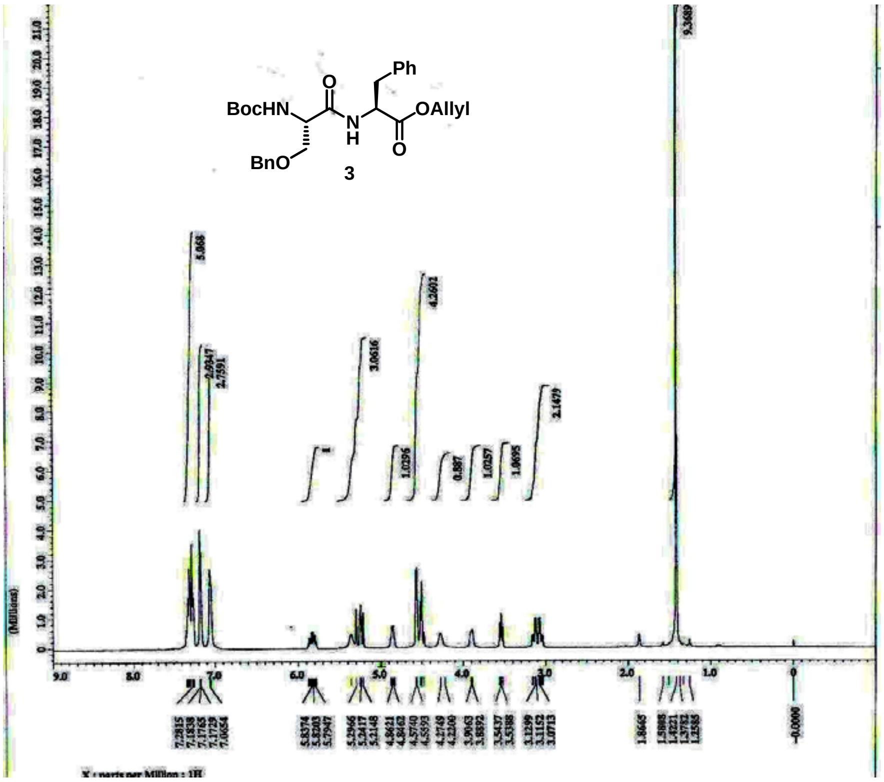

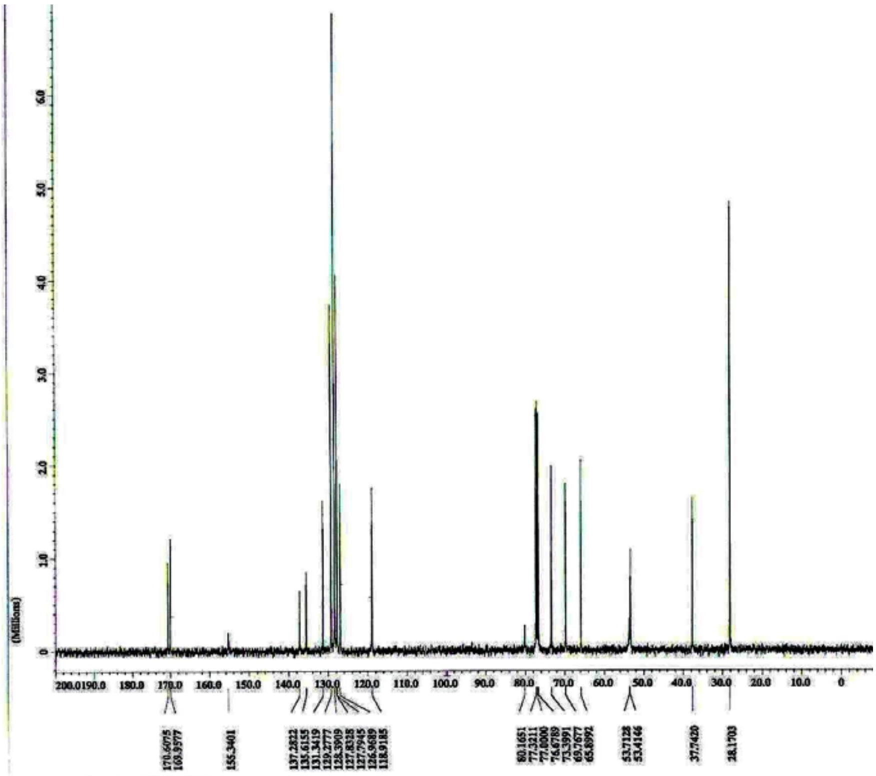

(*S*,*S*)-*N*,*N'*-bis(1-allyloxycarbonyl-2-phenylethyl)urea (S1)

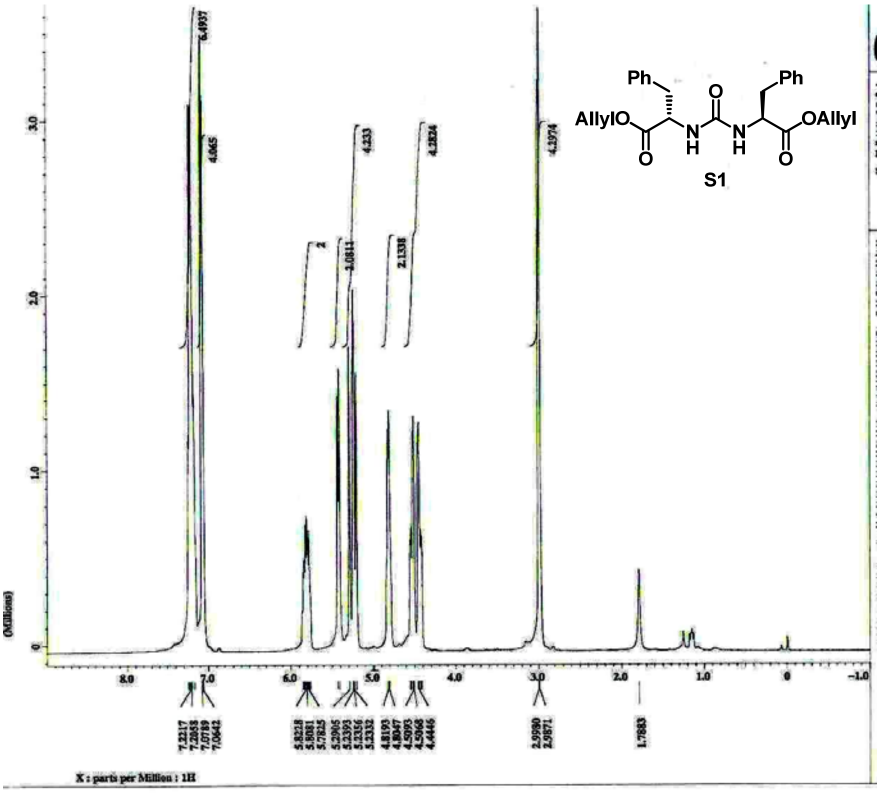

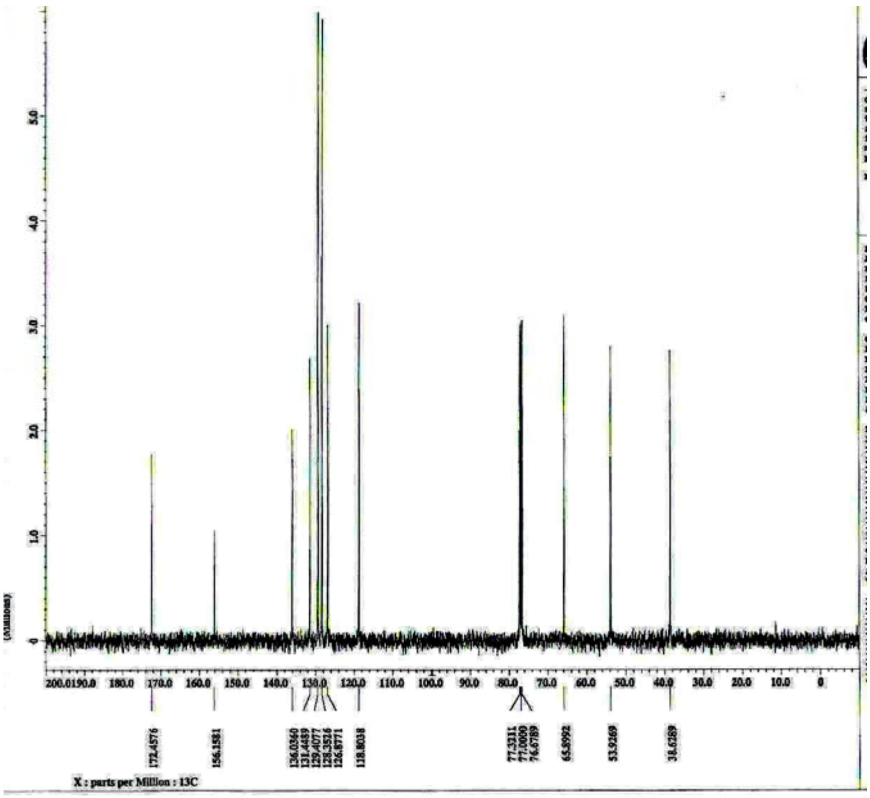

Boc-L-Ser(*O*-Bn)-OH (1) (recovered)

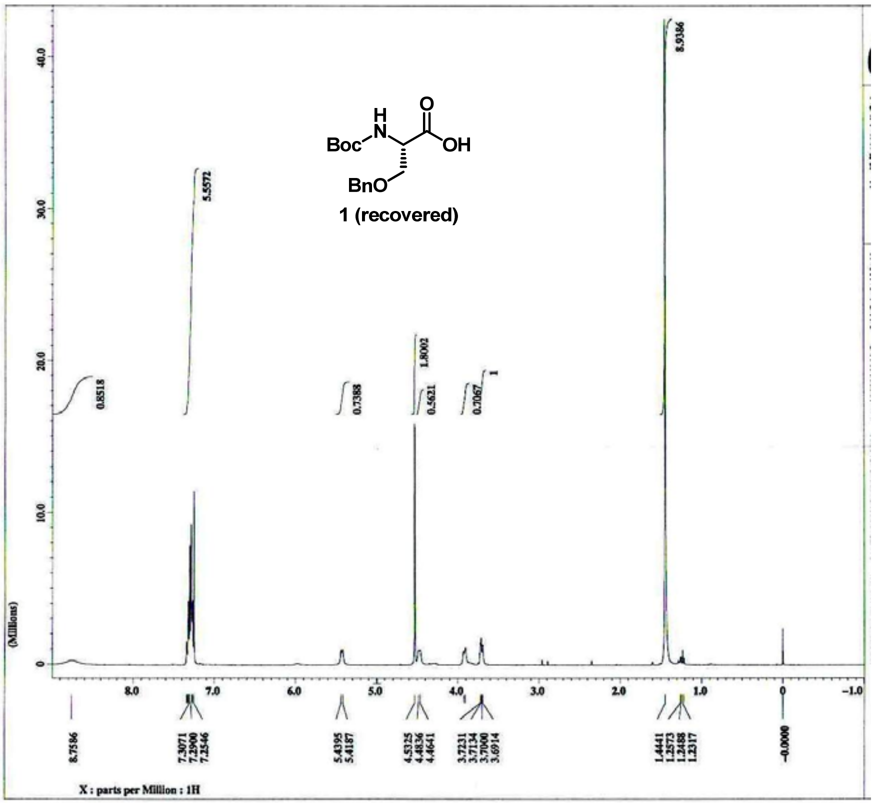

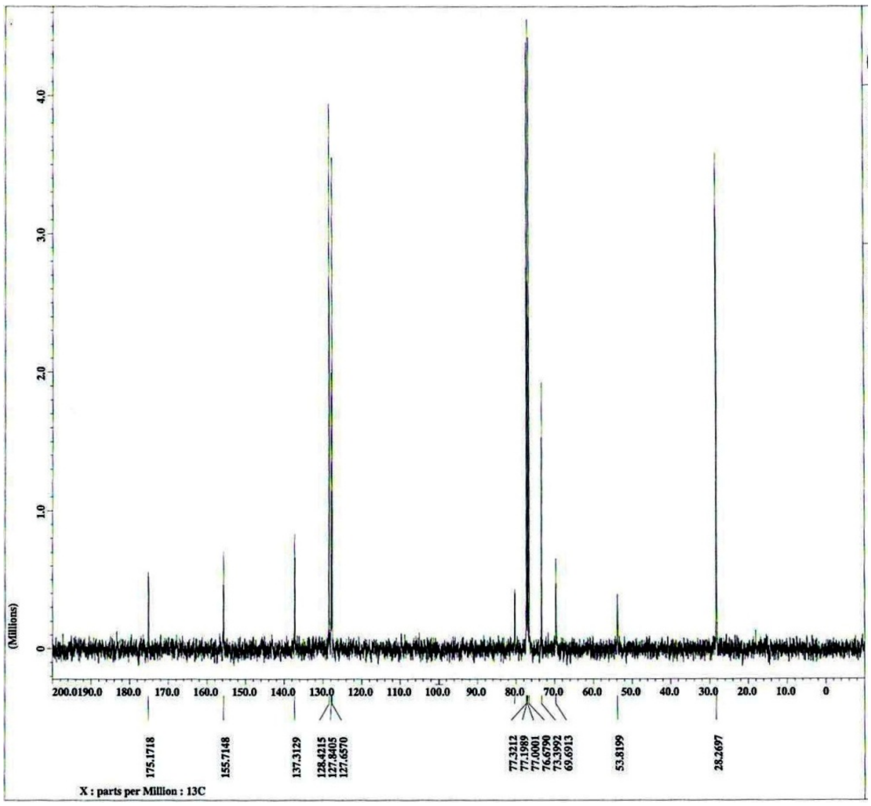

Boc-L-Tyr(*O*-Bn)-L-Phe-OAllyl (5)

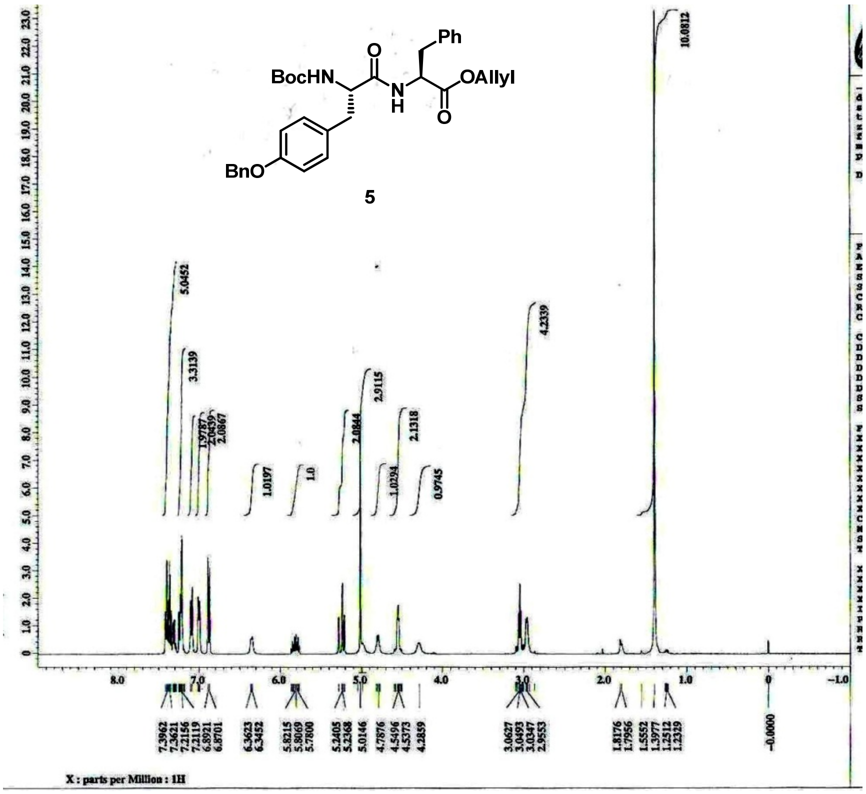

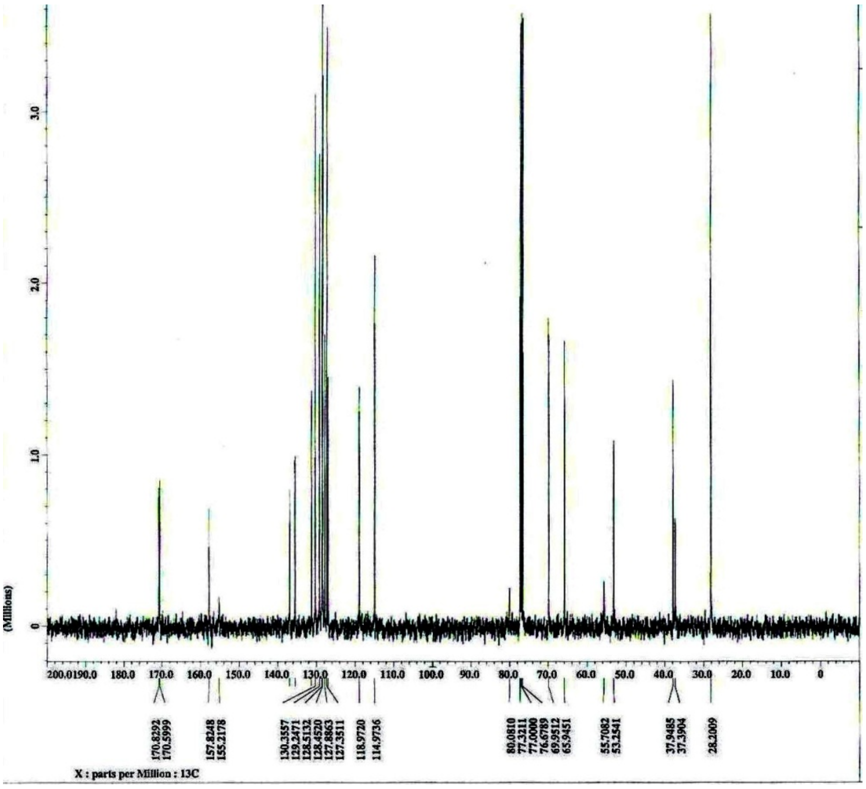

Fmoc-L-His(*N*-Trt)-L-Phe-OAllyl (6)

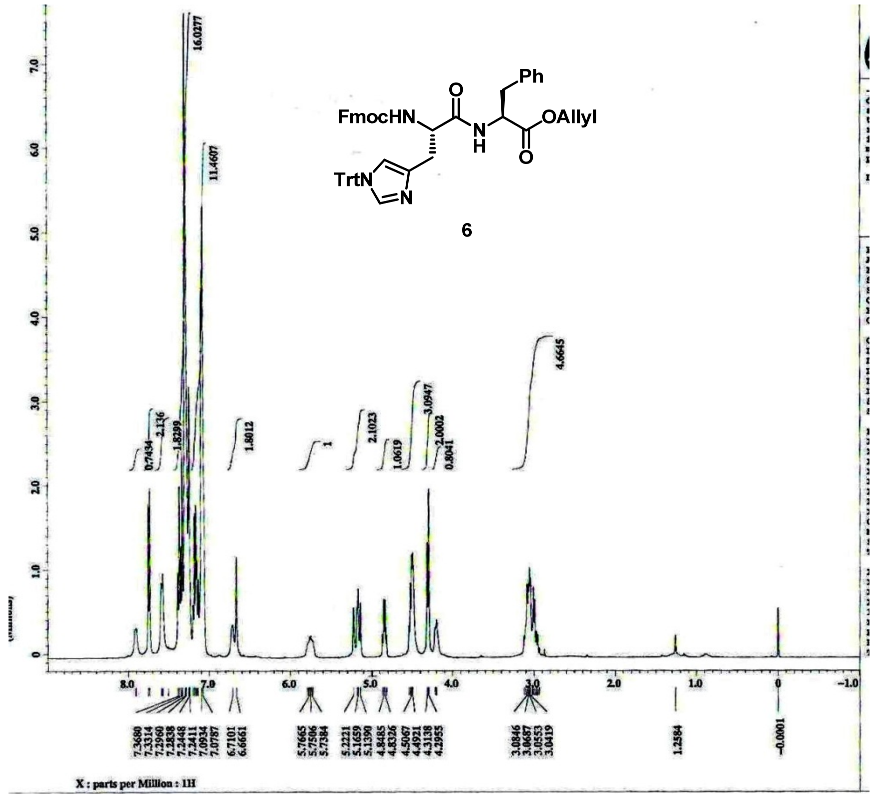

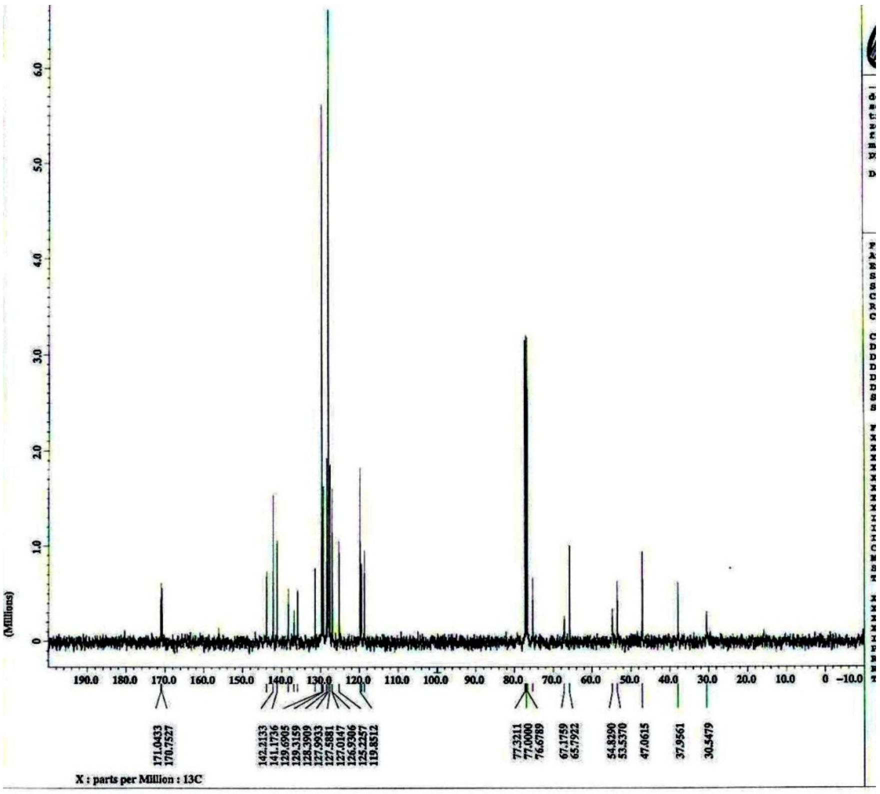

Fmoc-L-Cys(*S*-Trt)-L-Phe-OAllyl (7)

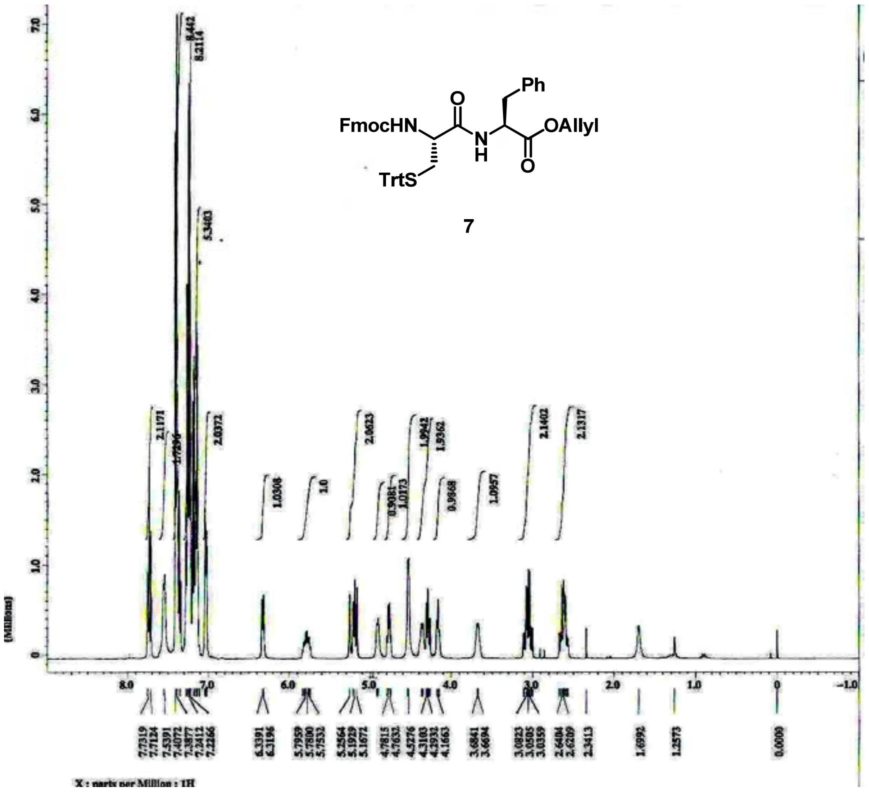

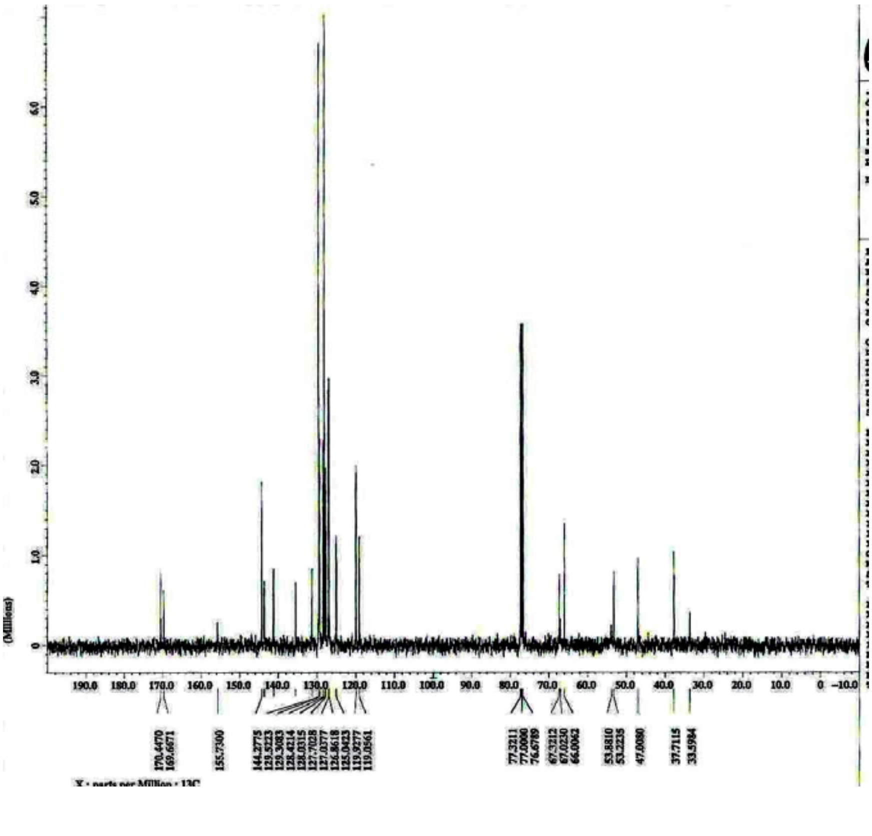

Boc-D-Phg-L-Phe-OAllyl (8)

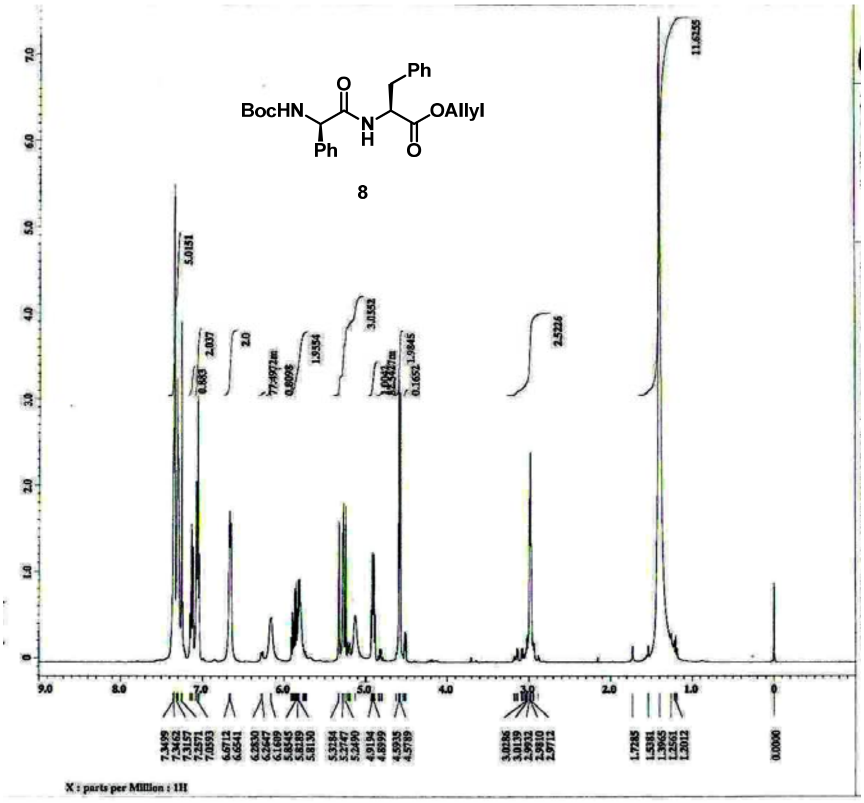

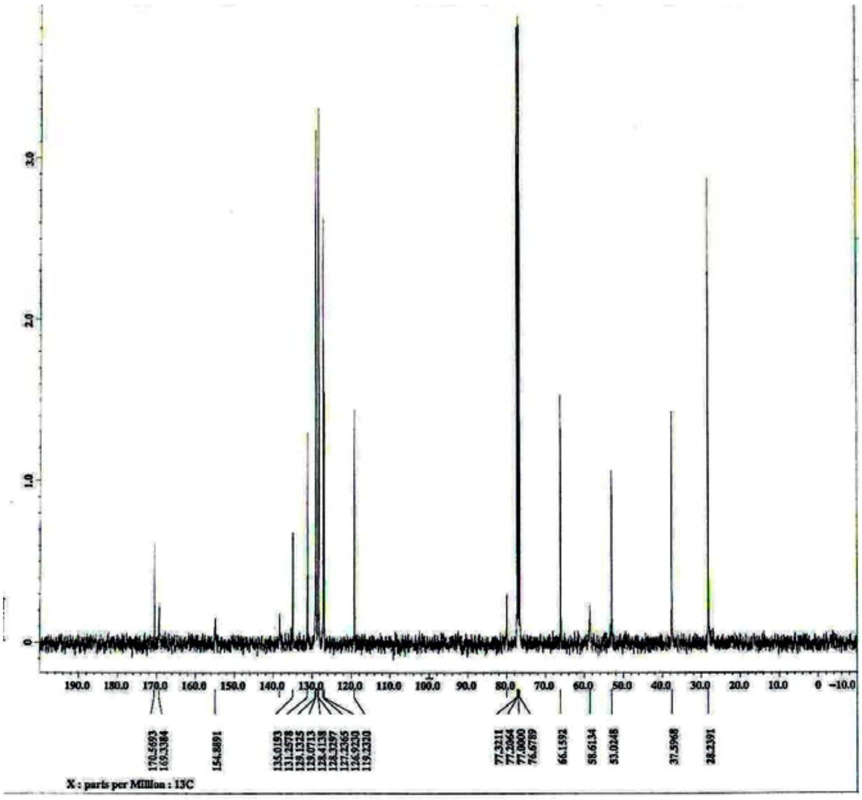

Boc-L-Ser(*O*-Bn)-L-Ile-OAllyl (9)

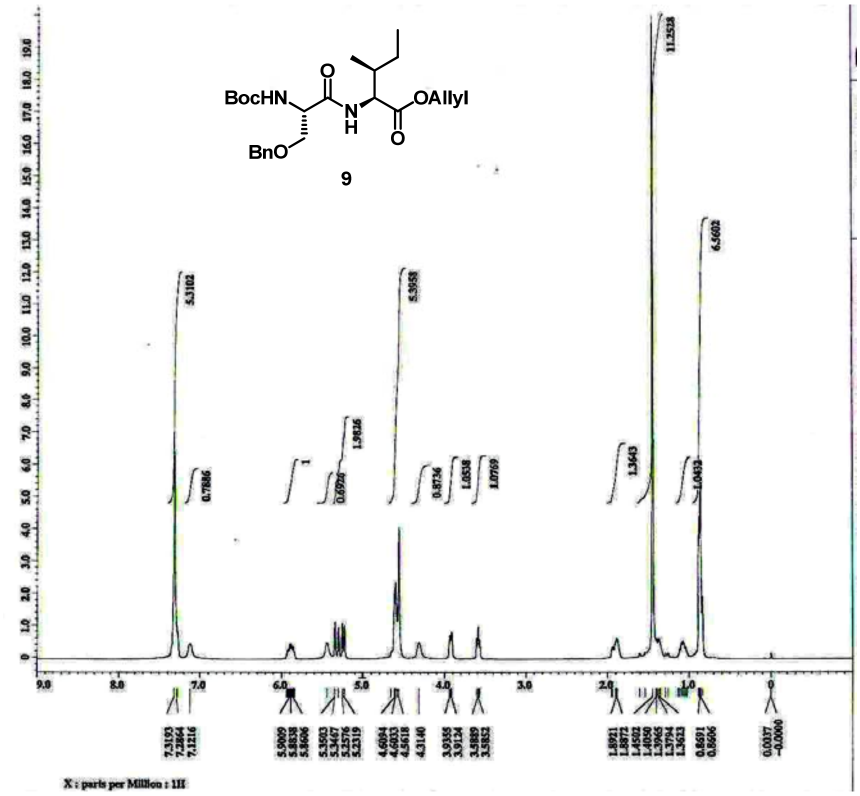

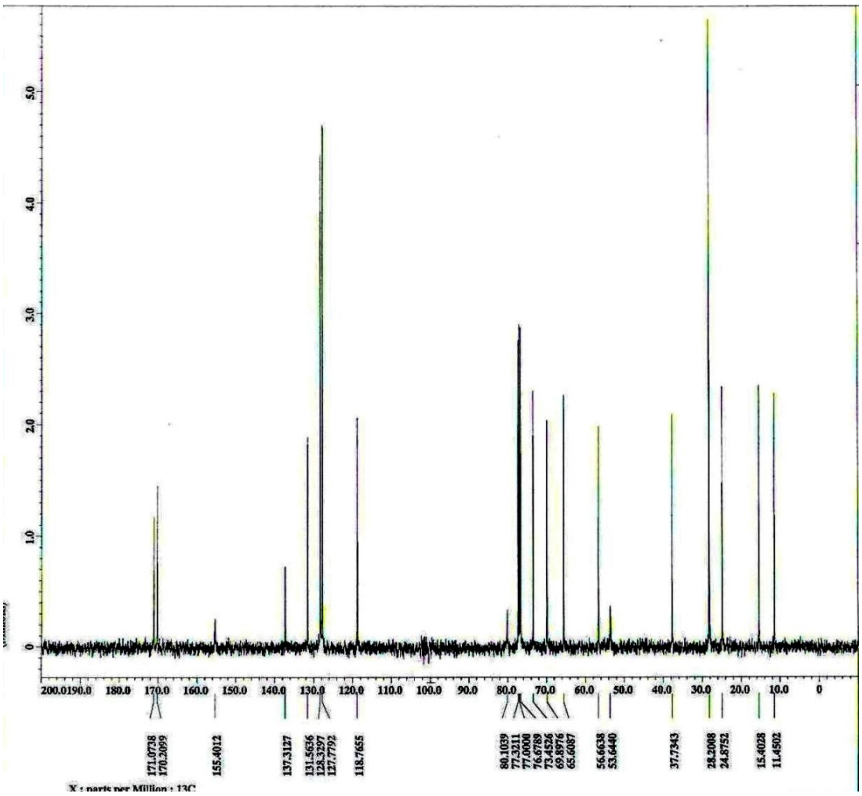

Boc-L-Ser(*O*-Bn)-L-Pro-OAllyl (10)

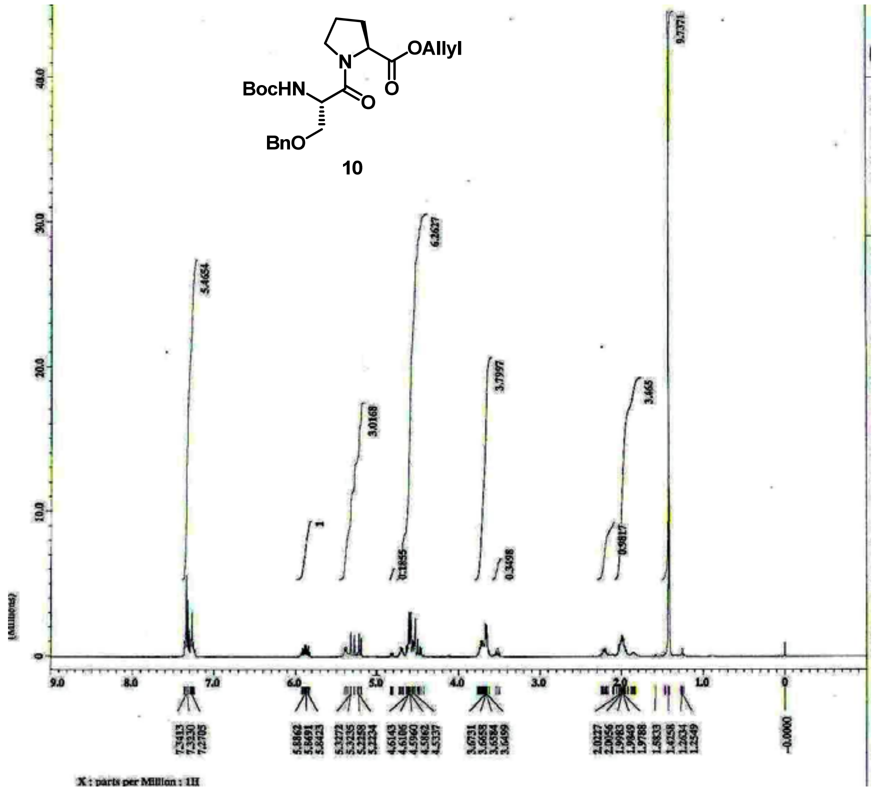

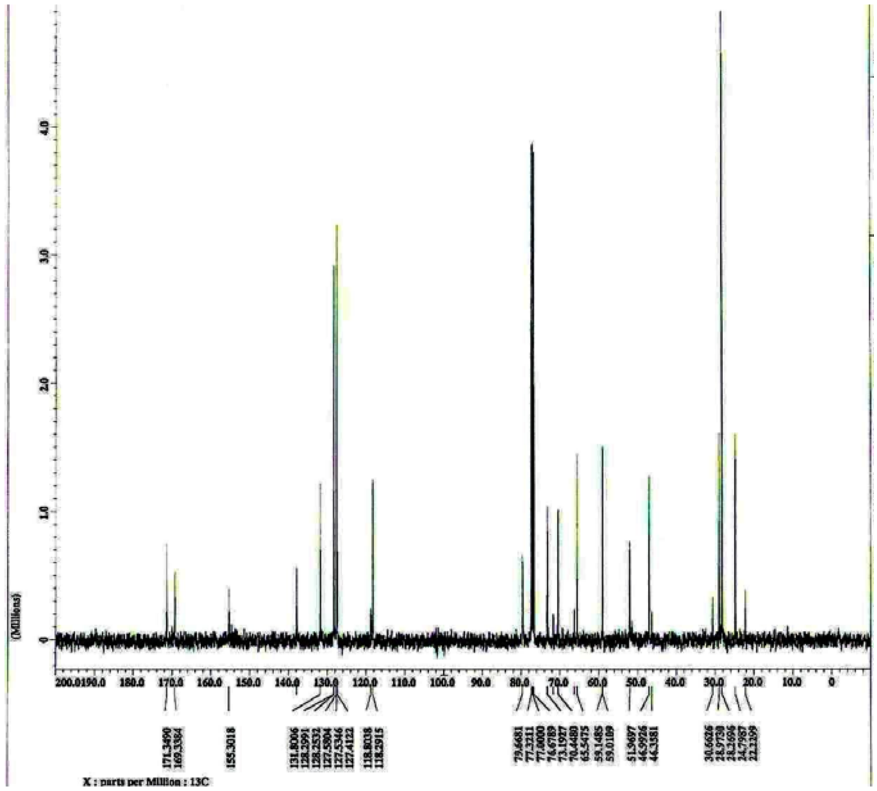

Boc-L-Ser(*O*-Bn)-Sar-OAllyl (11)

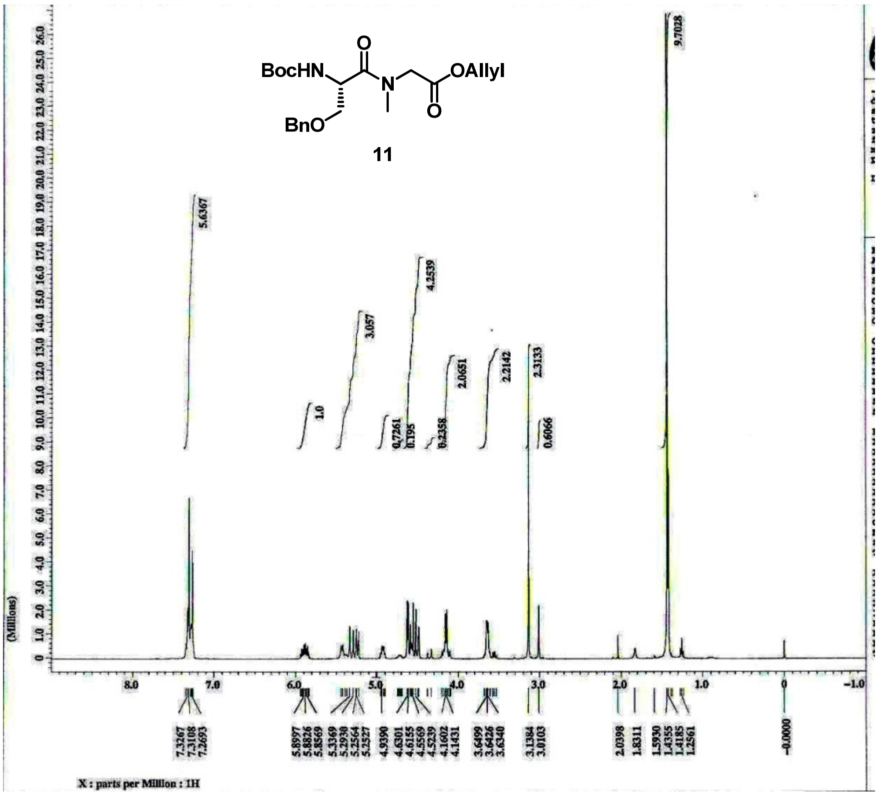

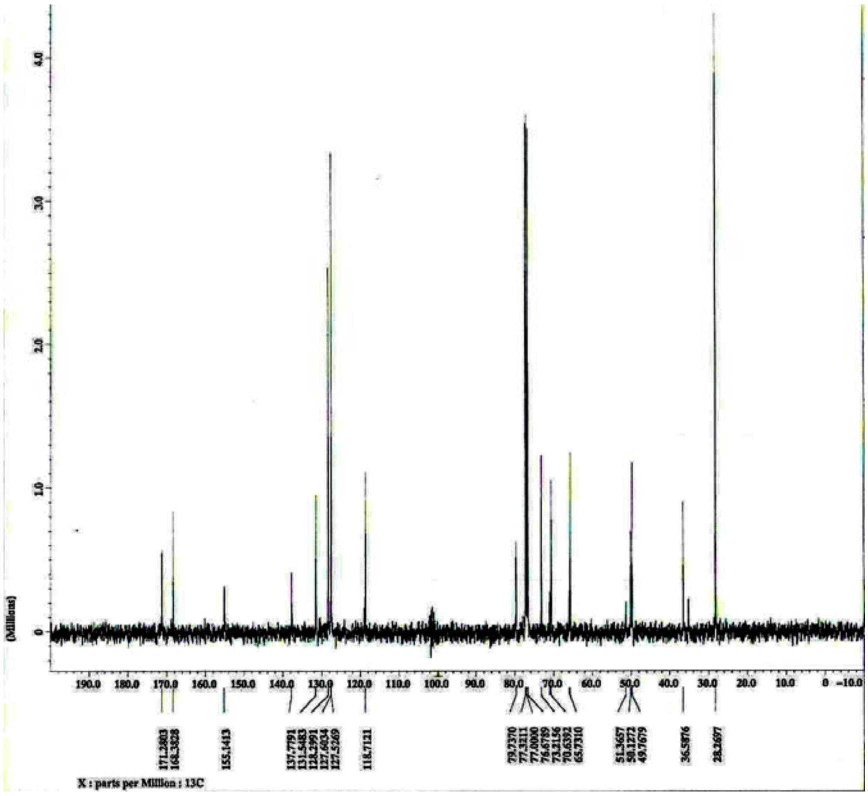

L-Lac-L-Phe-OAllyl (12)

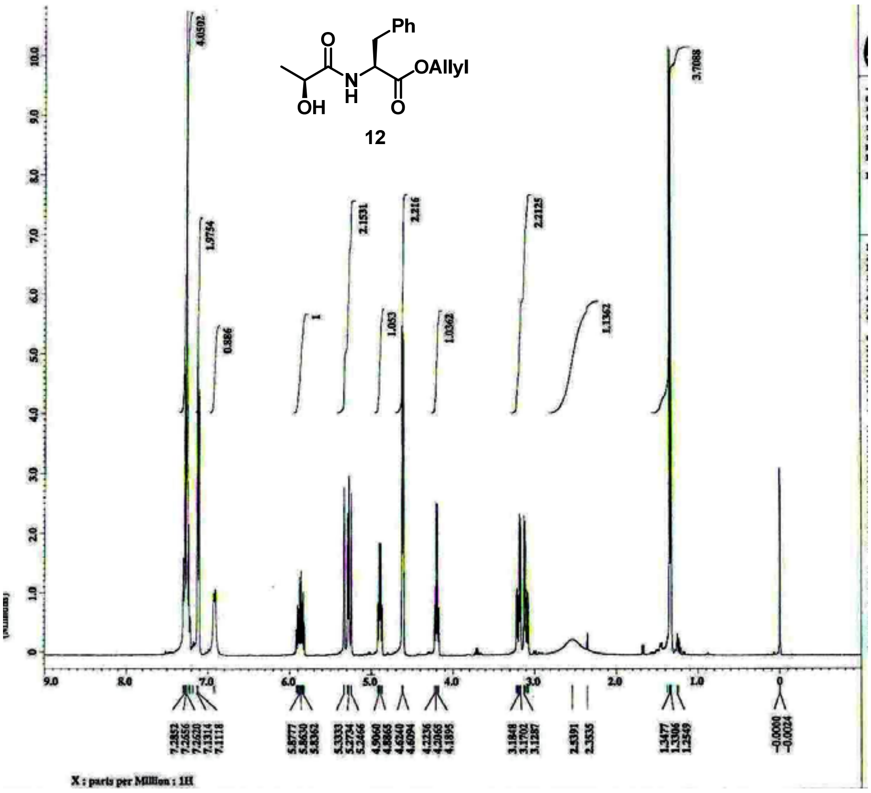

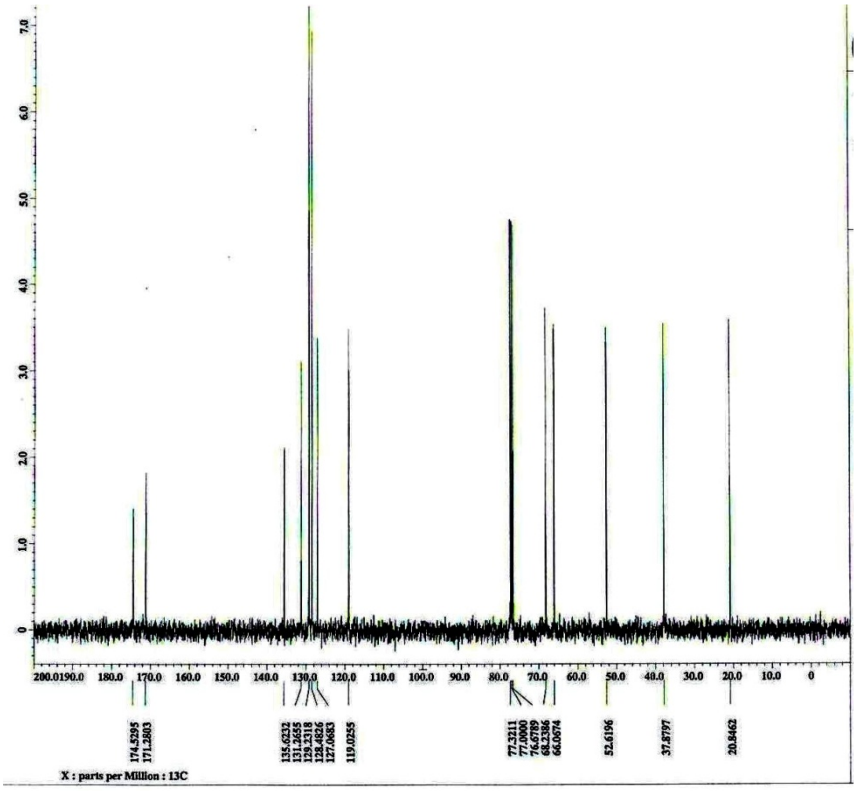

Boc-D-Ser(*O*-Bn)-L-Phe-OAllyl (4)

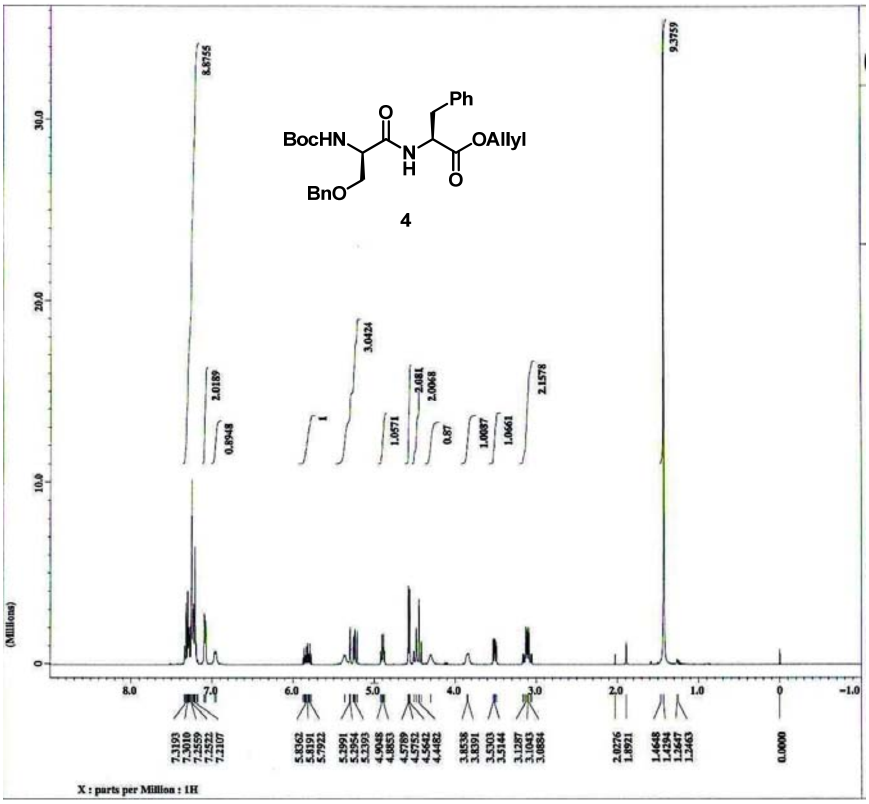

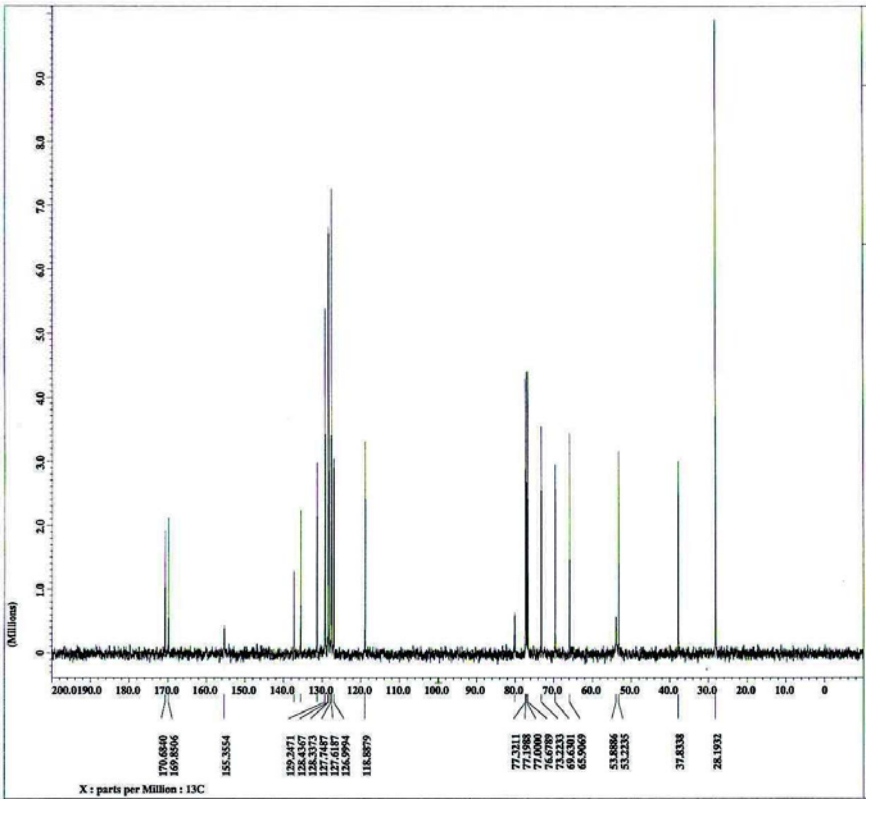

Boc-D-Tyr(*O*-Bn)-L-Phe-OAllyl (S2)

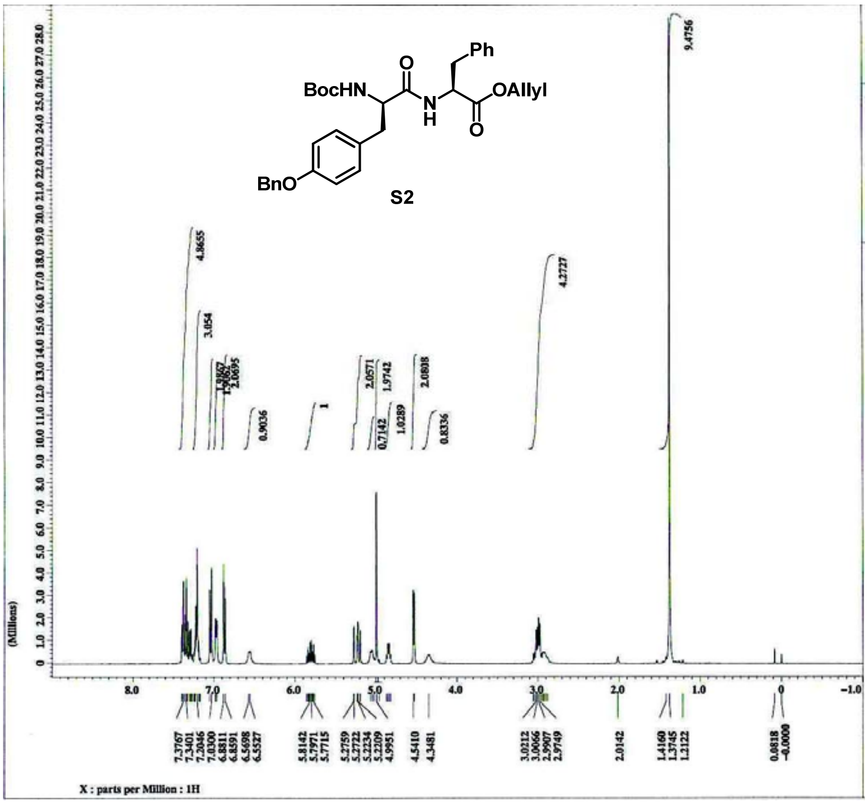

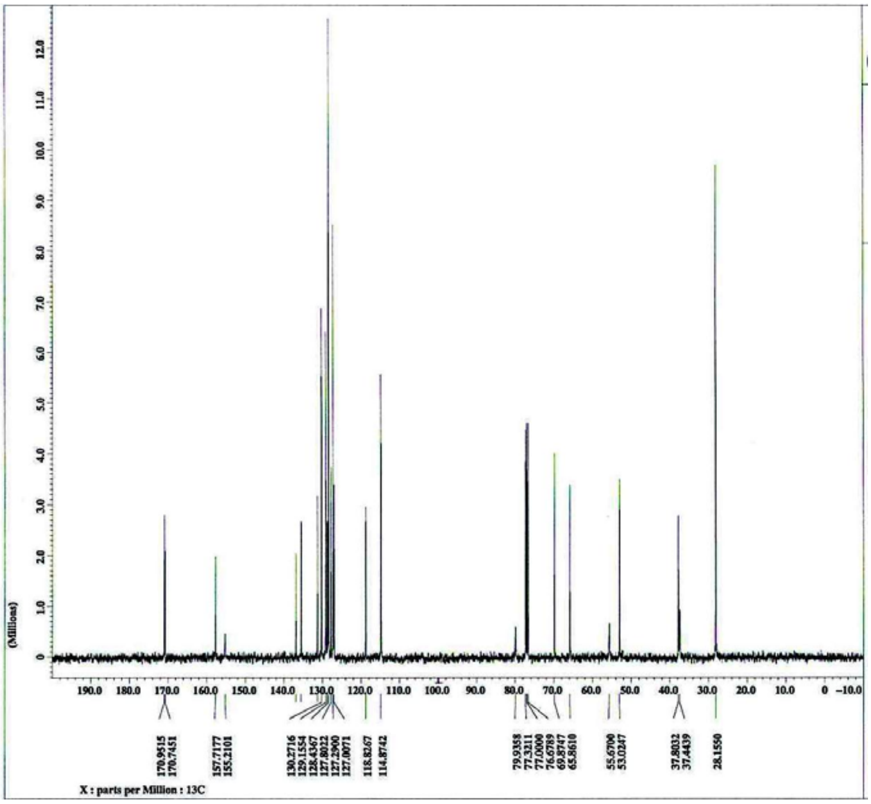

Fmoc-D-His(*N*-Trt)-L-Phe-OAllyl (S3)

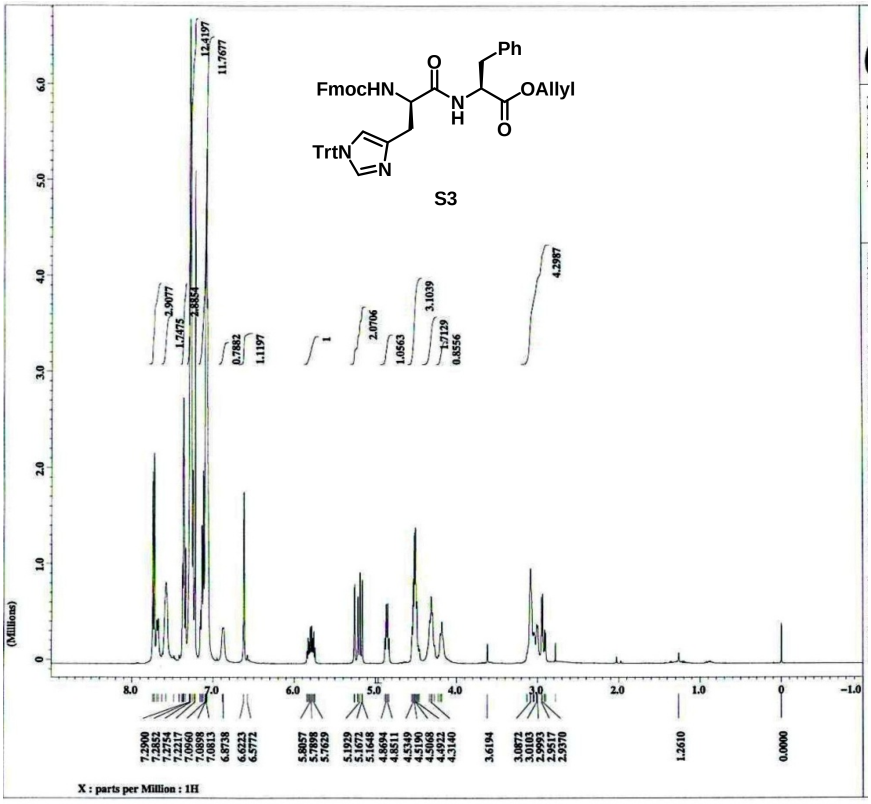

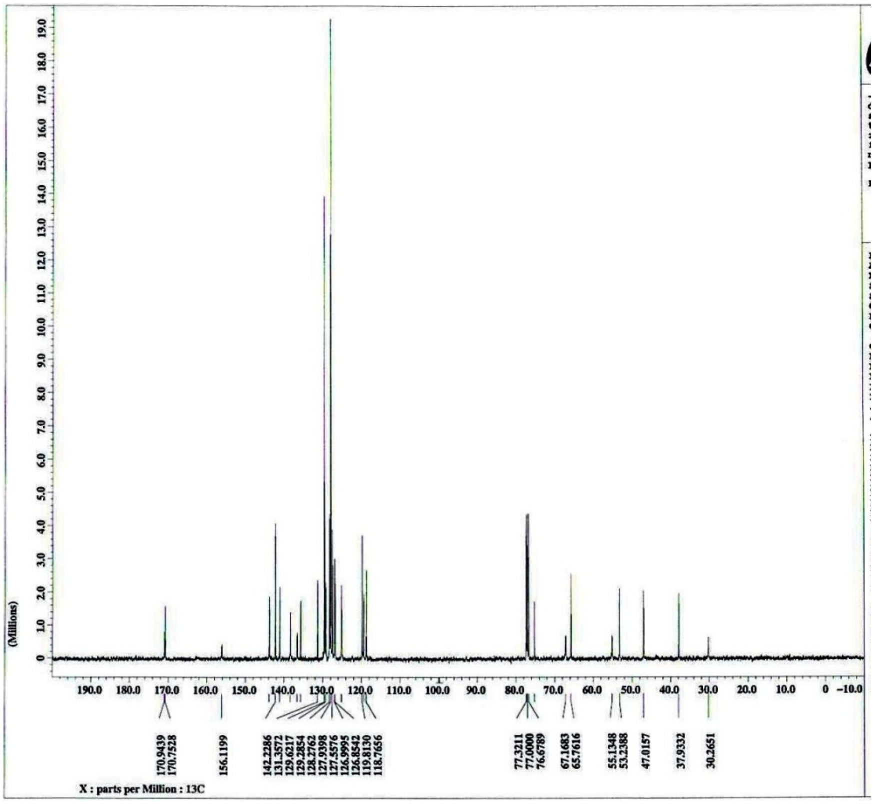

Fmoc-D-Cys(*S*-Trt)-L-Phe-OAllyl (S4)

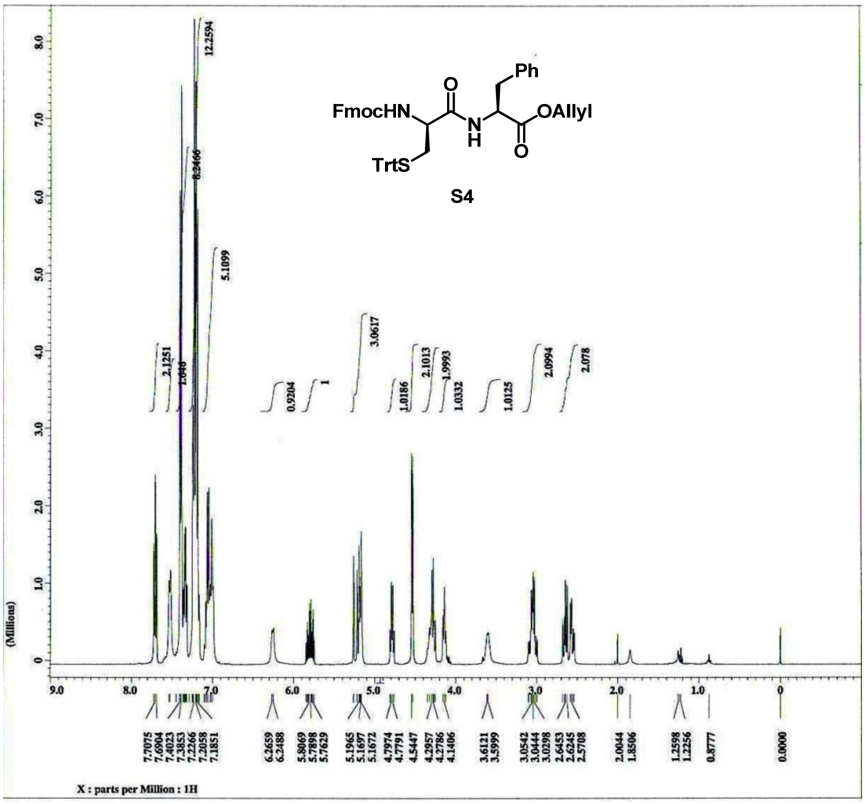

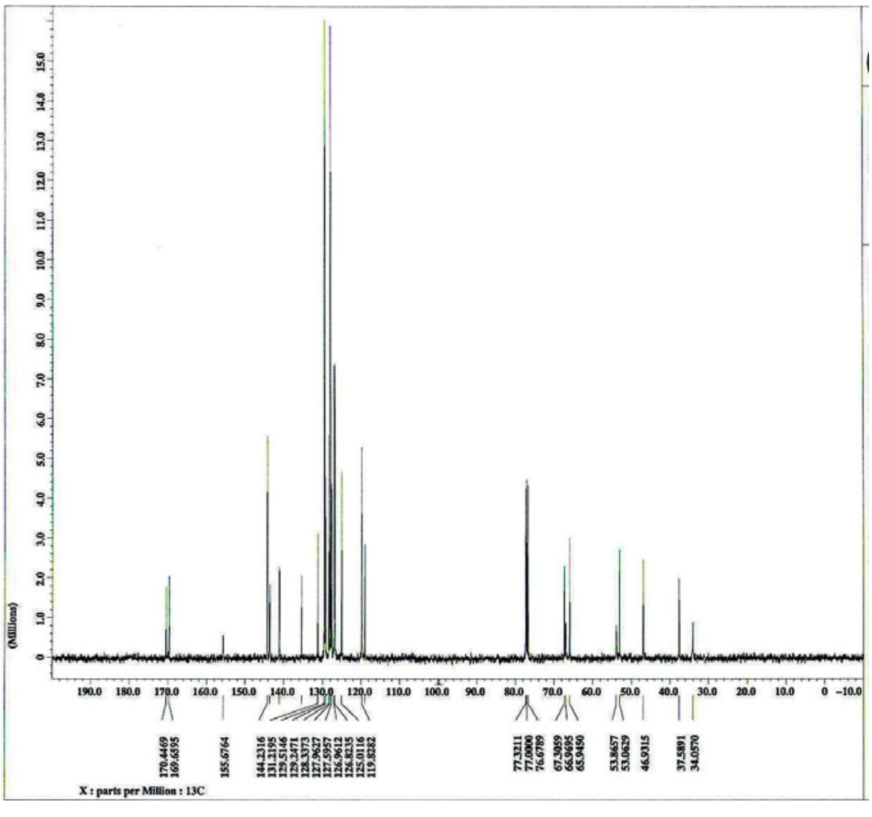

Boc-L-Phg-L-Phe-OAllyl (S5)

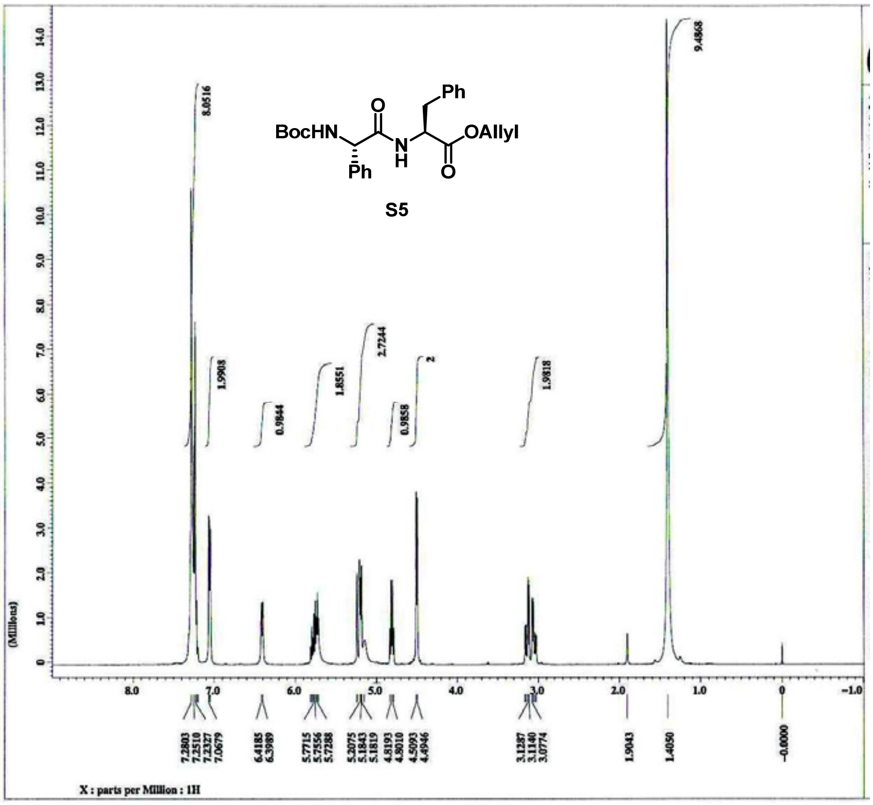

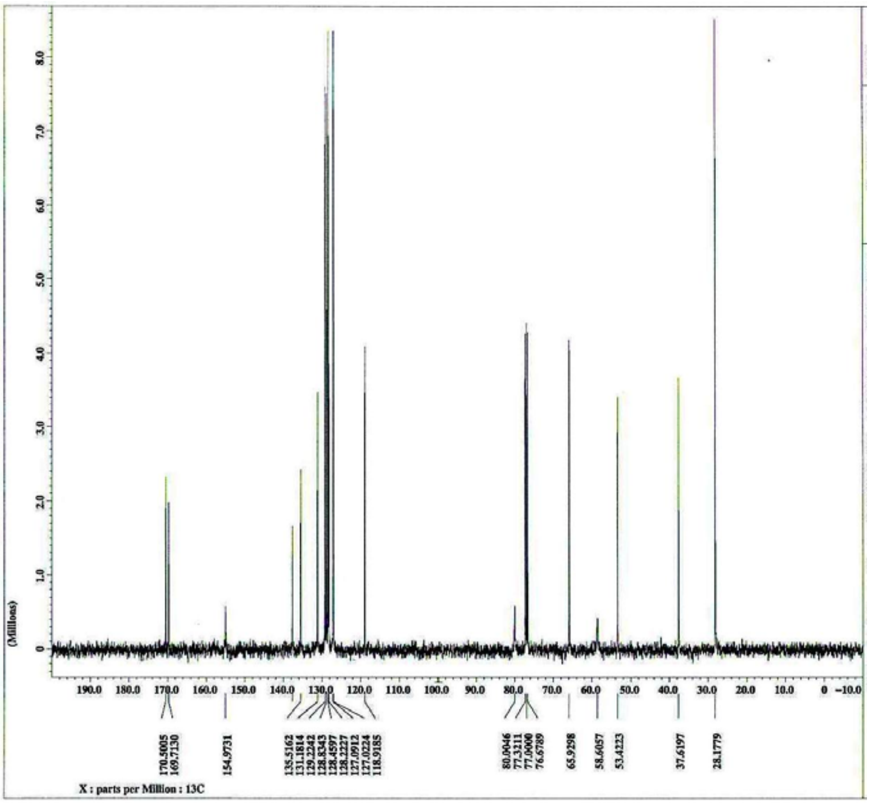

Boc-D-Ser(*O*-Bn)-L-Ile-OAllyl (S6)

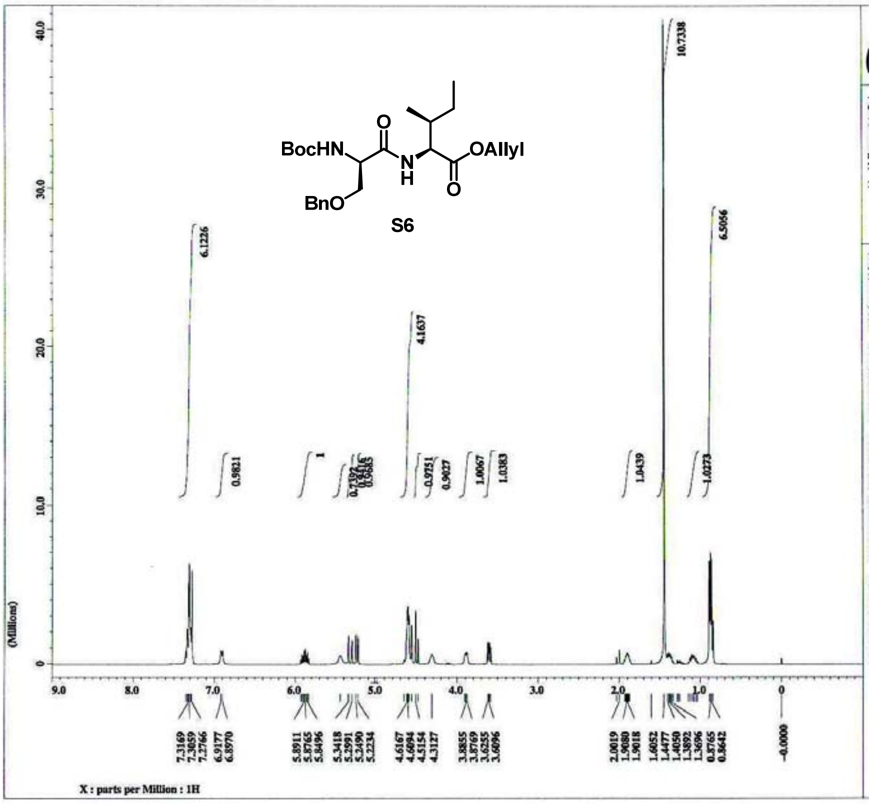

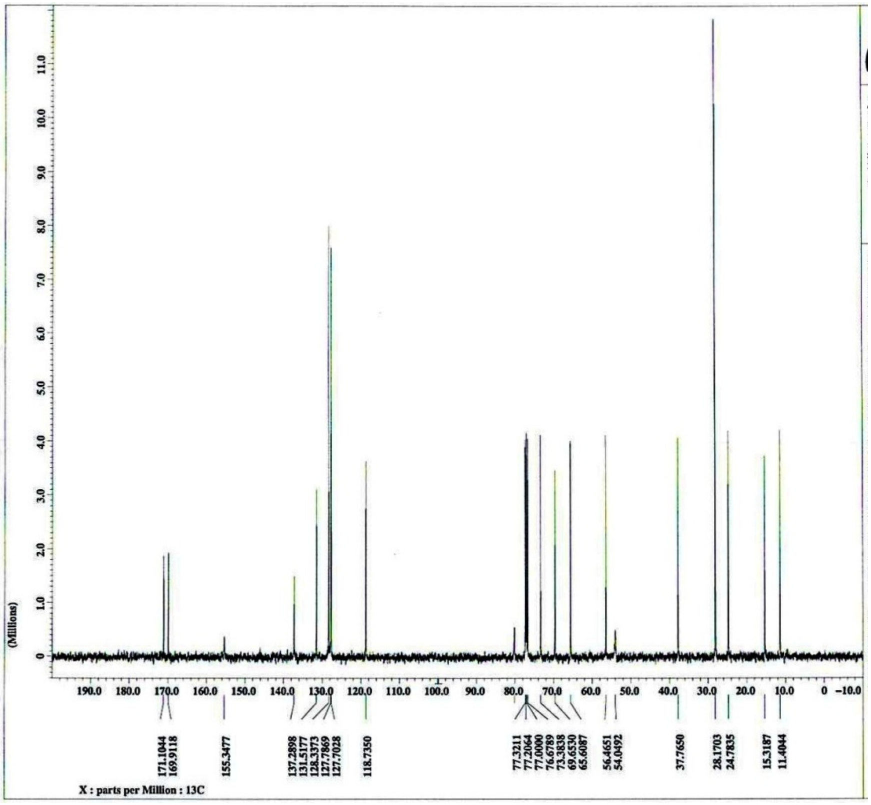

Boc-D-Ser(*O*-Bn)-L-Pro-OAllyl (S7)

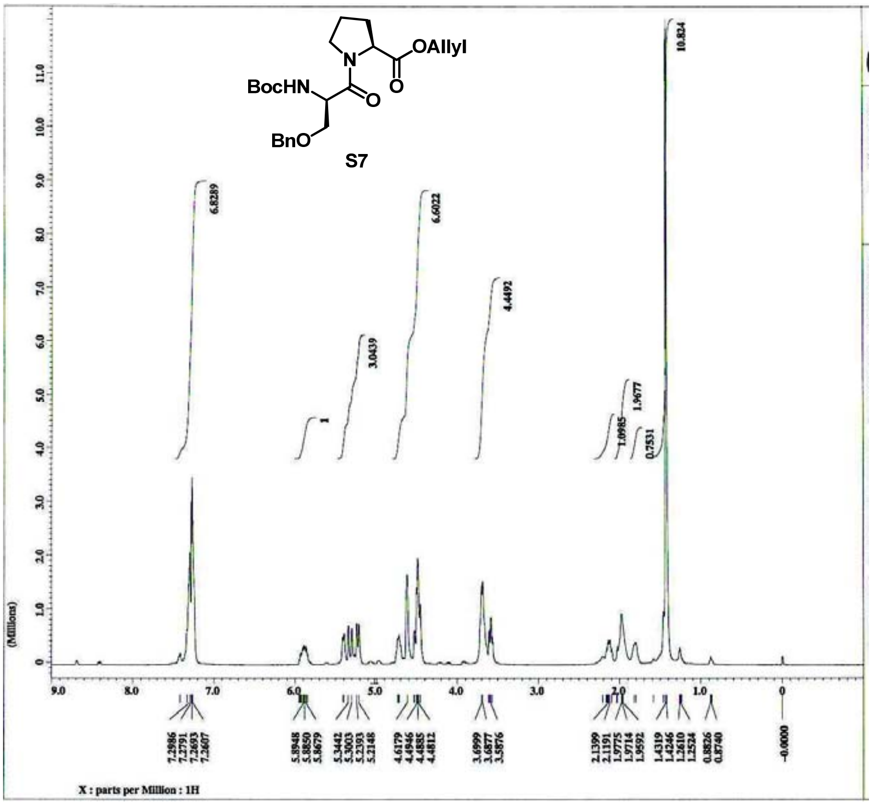

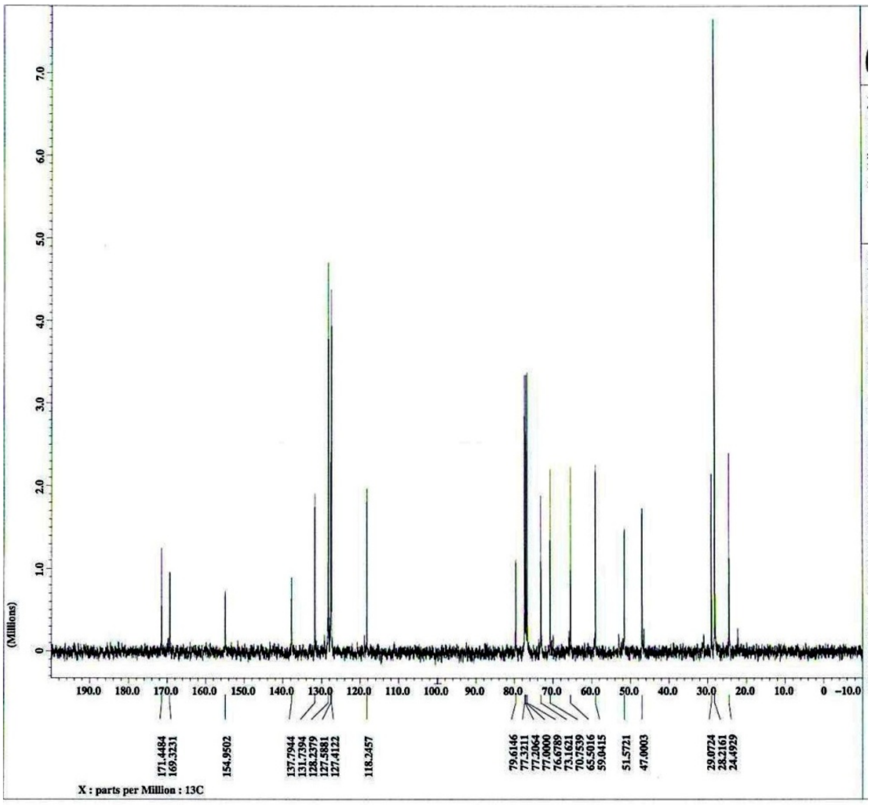

Boc-D-Ser(*O*-Bn)-Sar-OAllyl (S8)

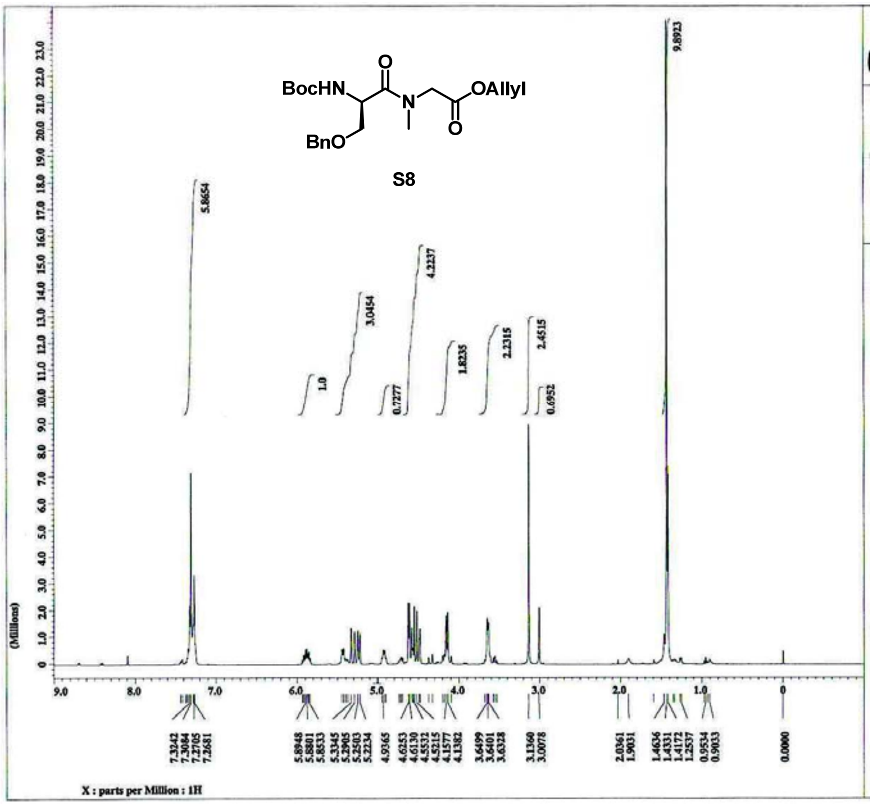

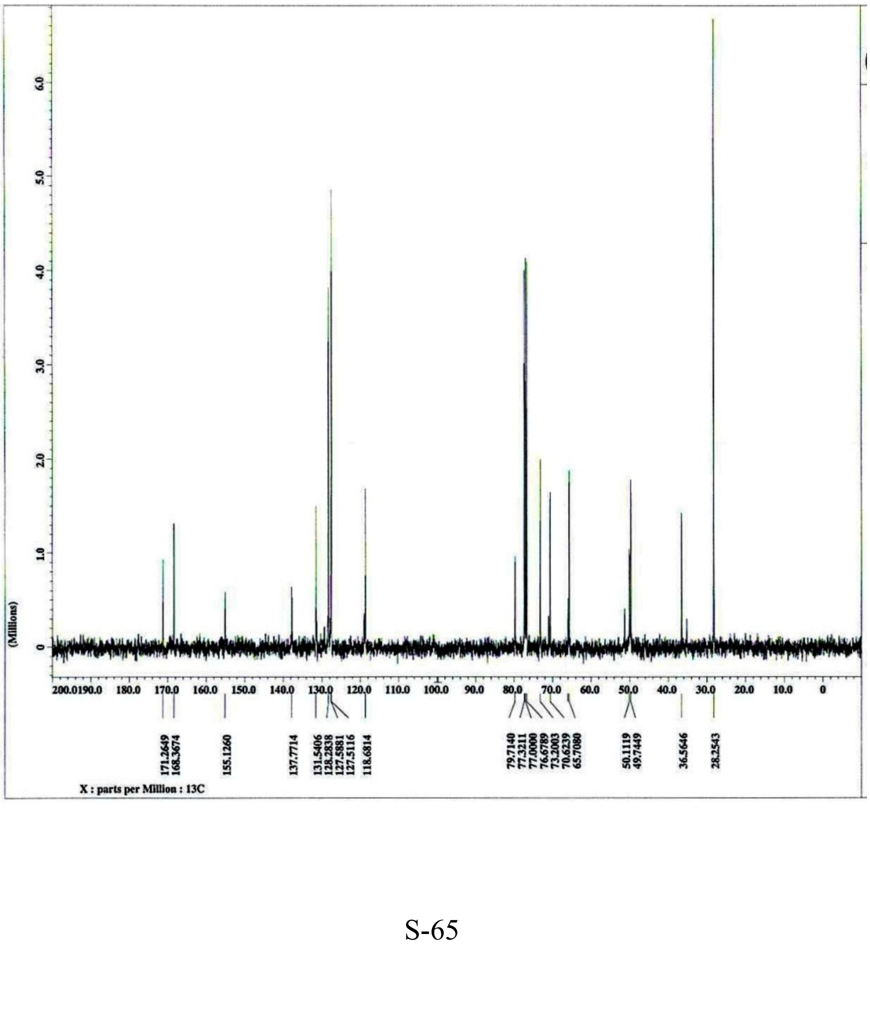

DL-Lac-L-Phe-OAllyl (S9)

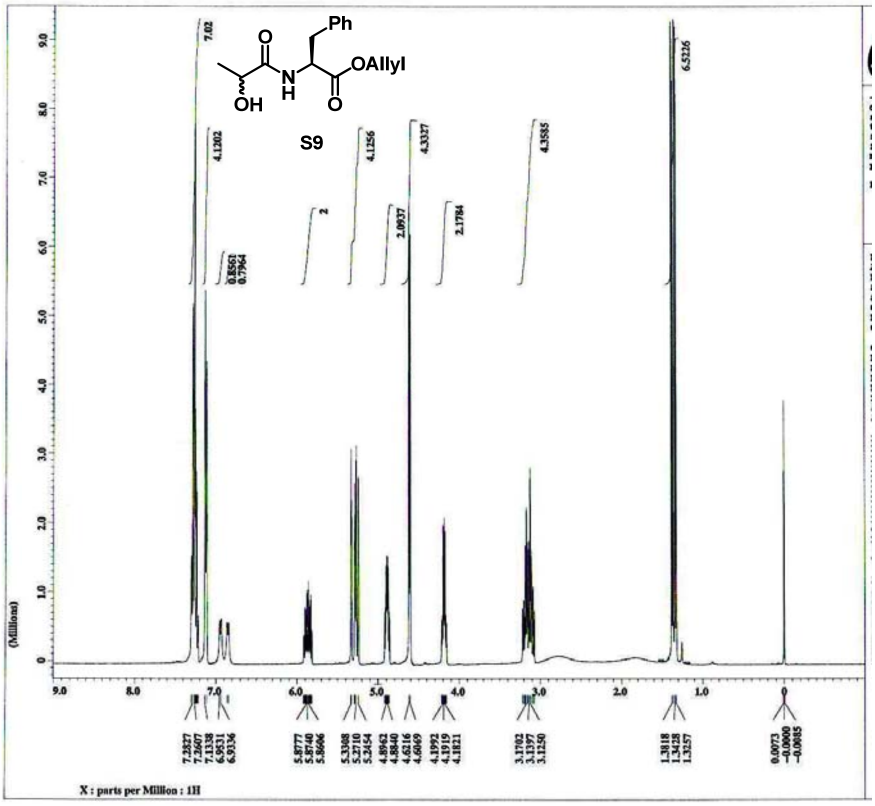

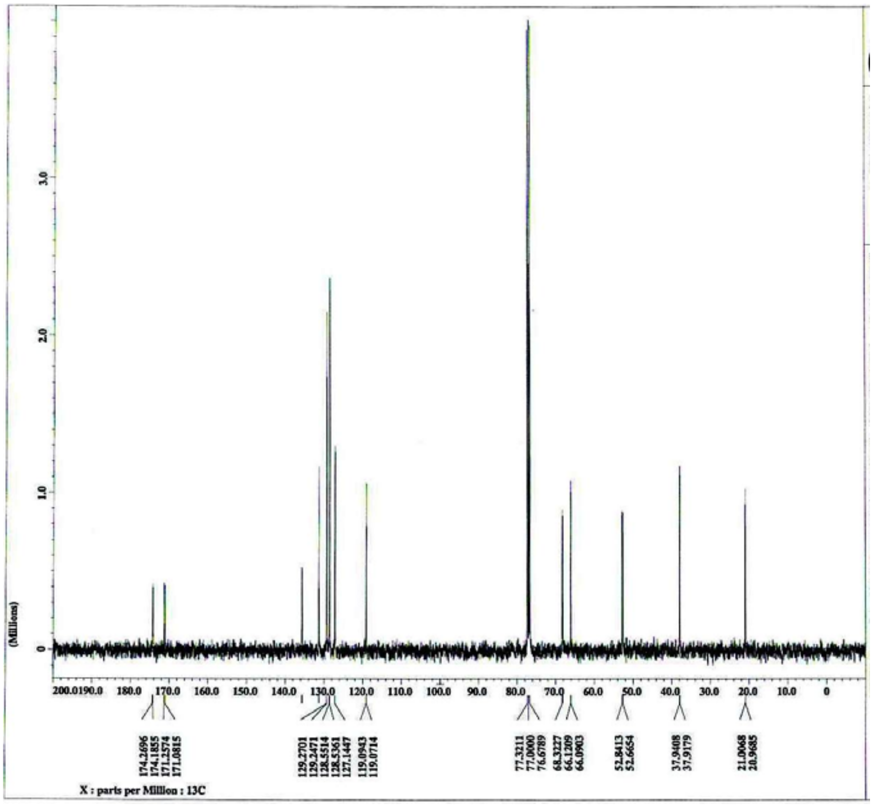

Fmoc-L-Val-OAllyl (S12)

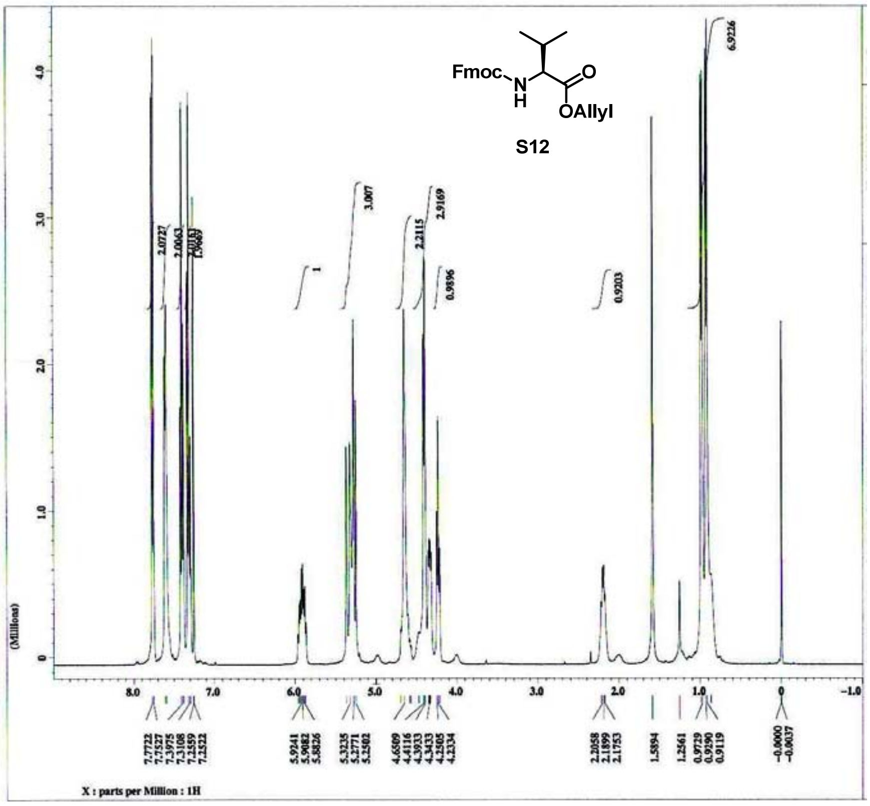

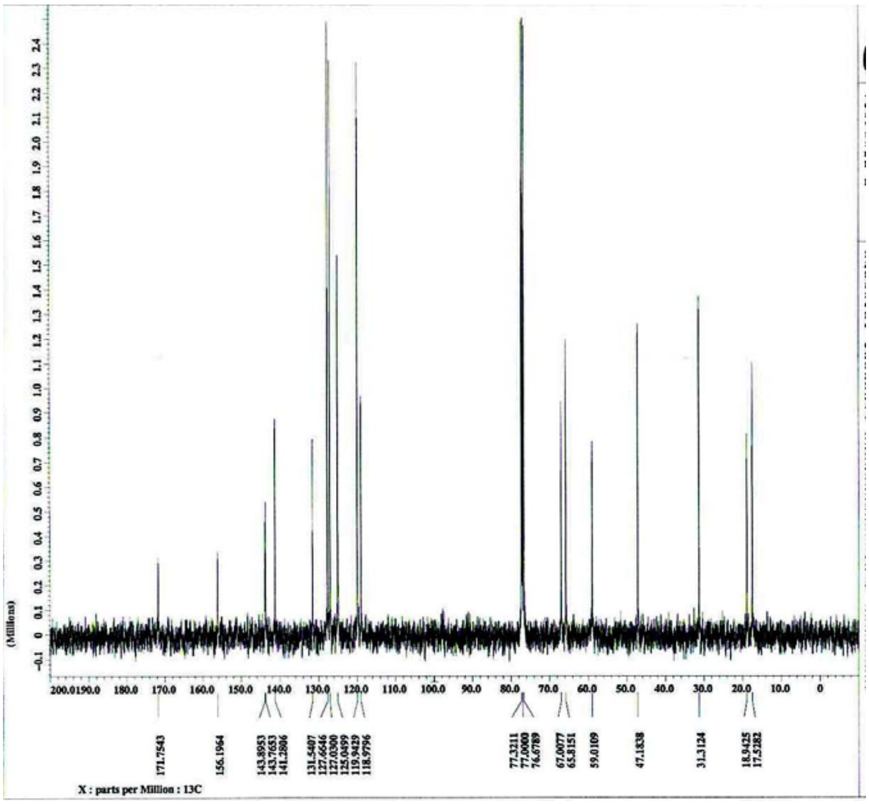

Fmoc-Sar-L-Val-OAllyl (15)

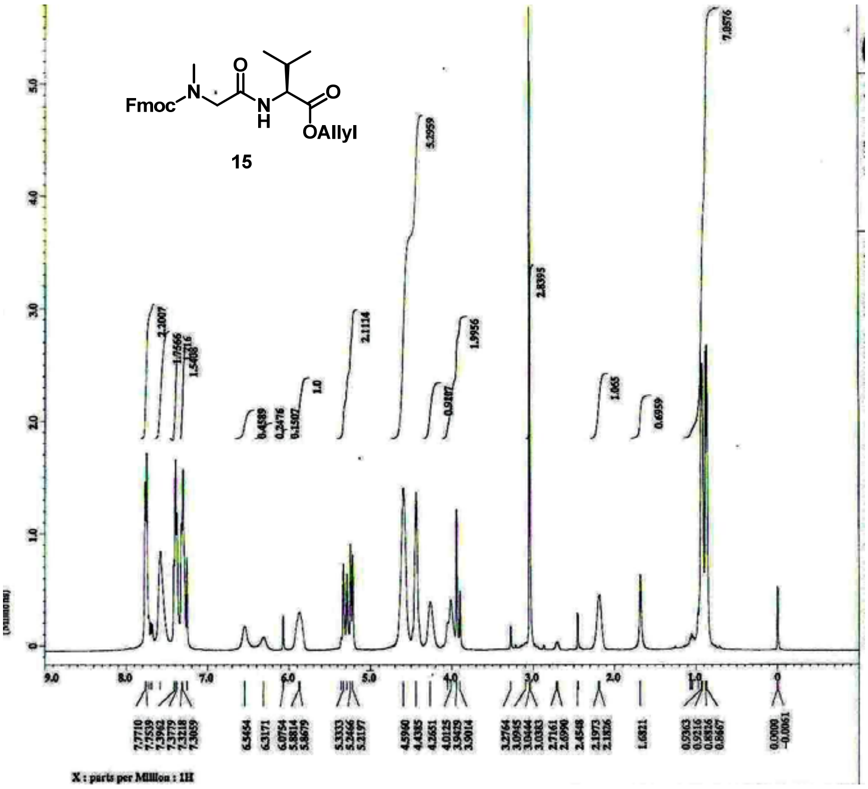

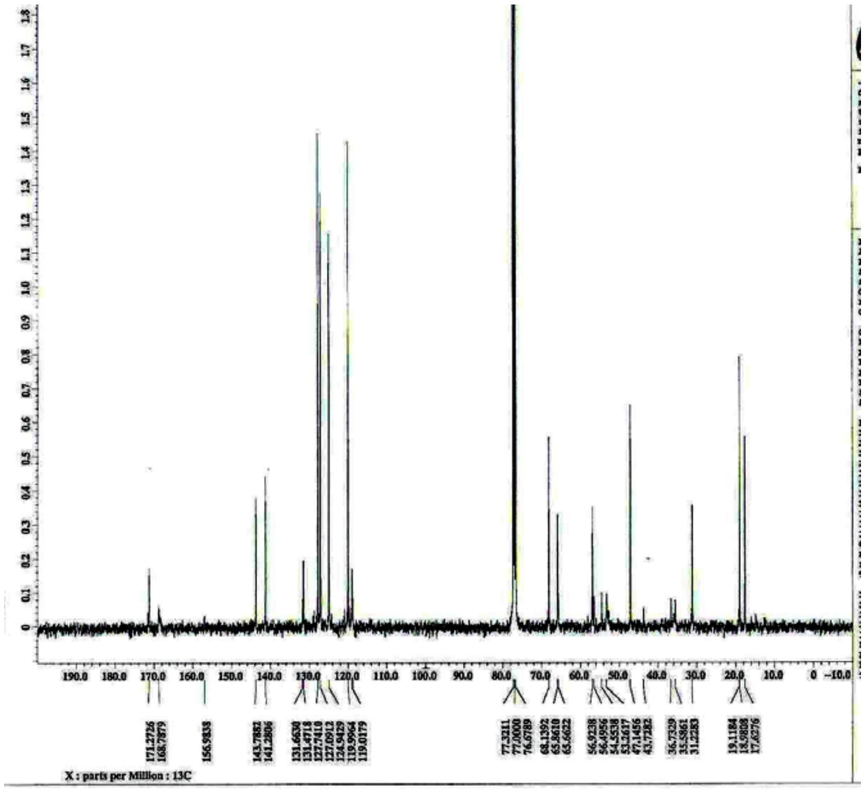

Fmoc-D-(*N*-Me)Leu-Sar-L-Val-OAllyl (16)

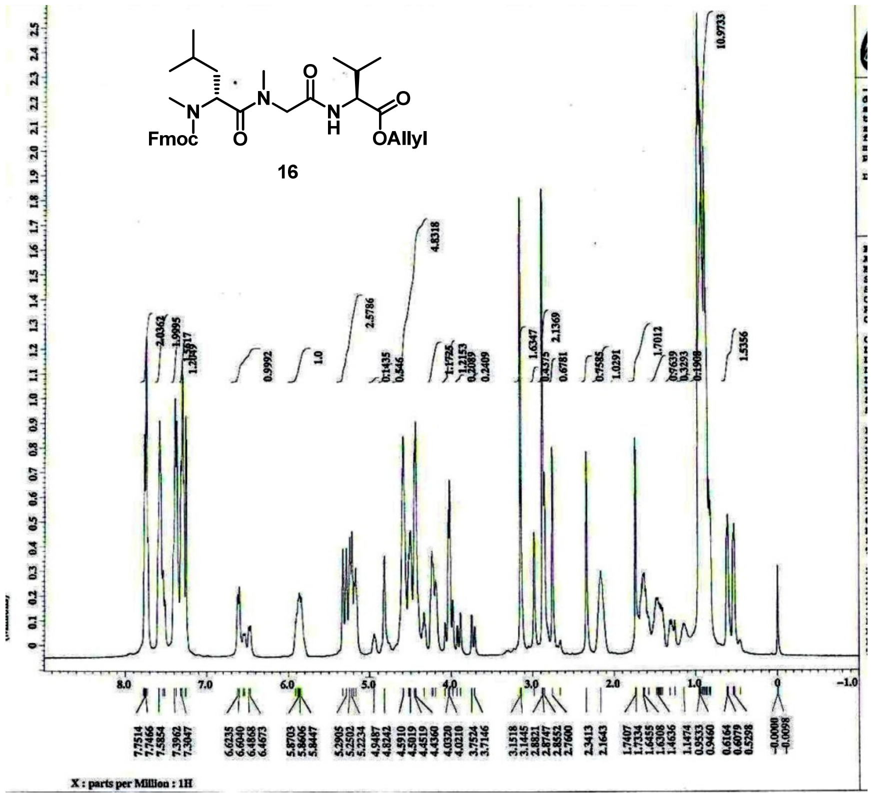

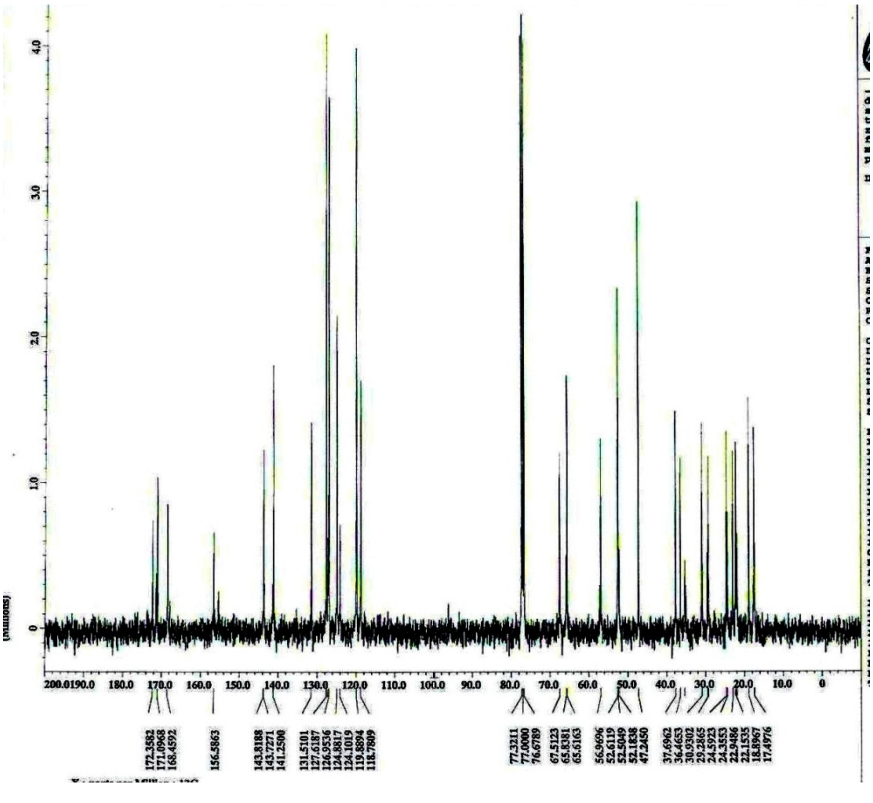

Fmoc-L-Val-D-(*N*-Me)Leu-Sar-L-Val-OAllyl (17)

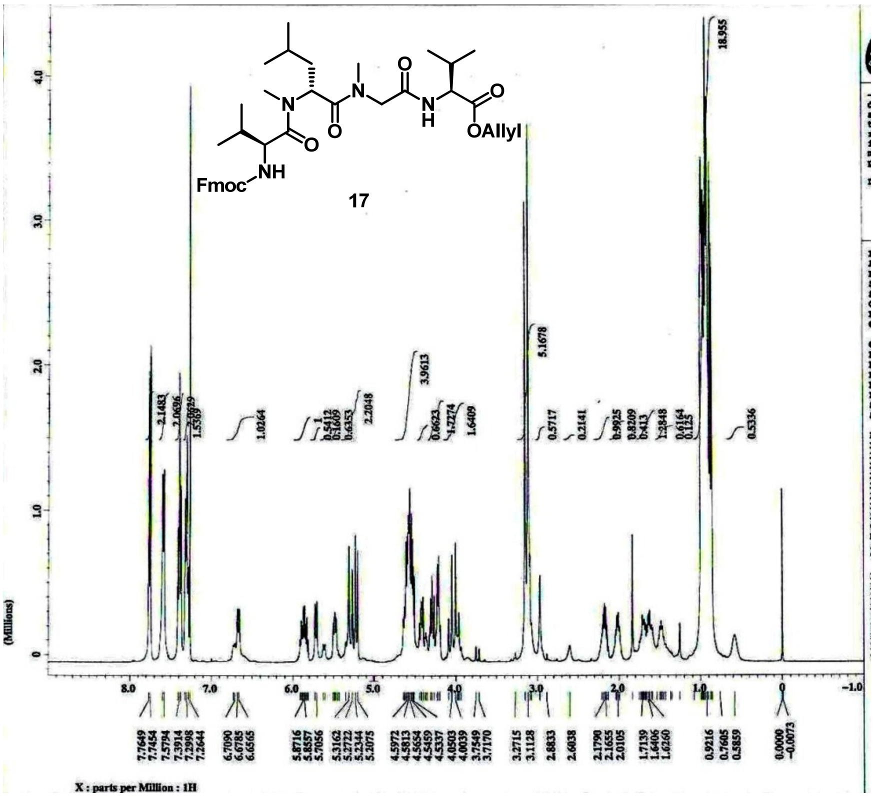

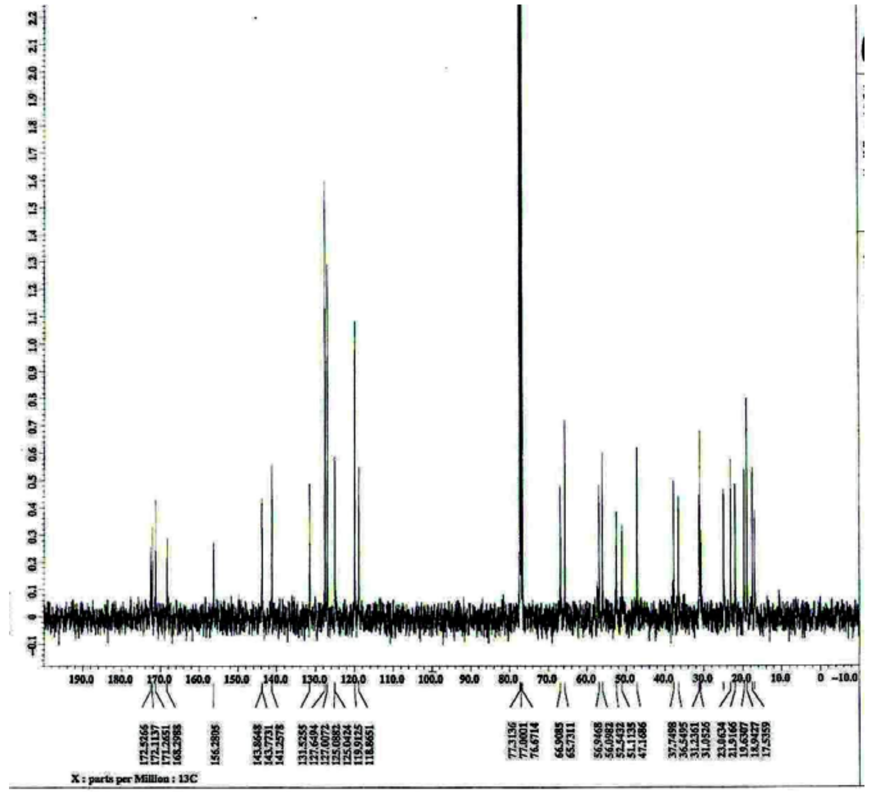

Fmoc-D-(*N*-Me)Leu-OAllyl (S17)

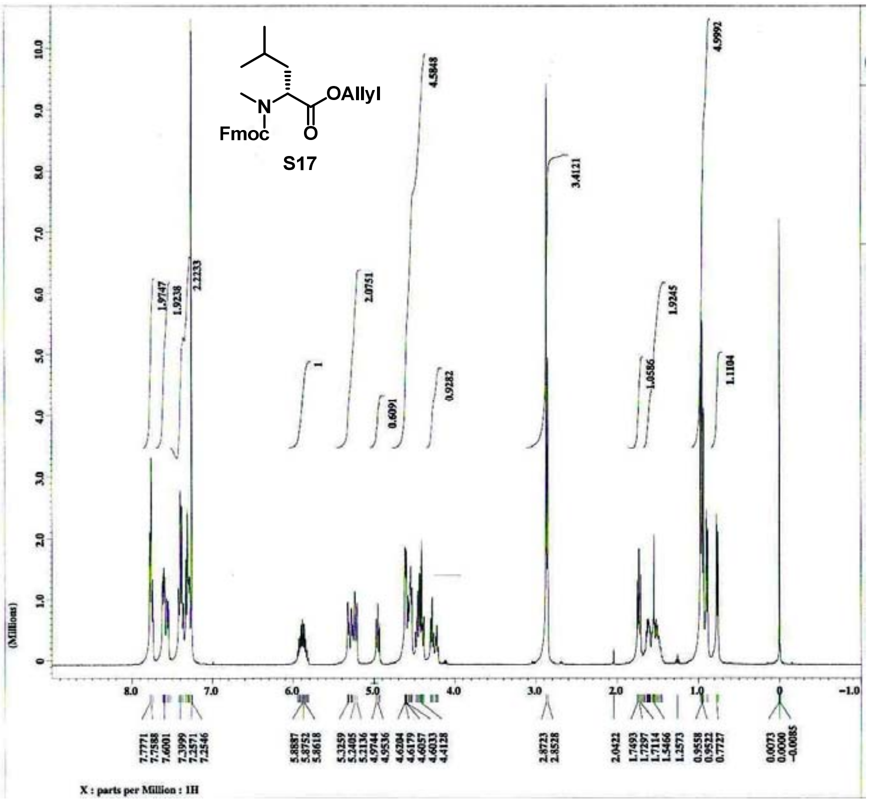

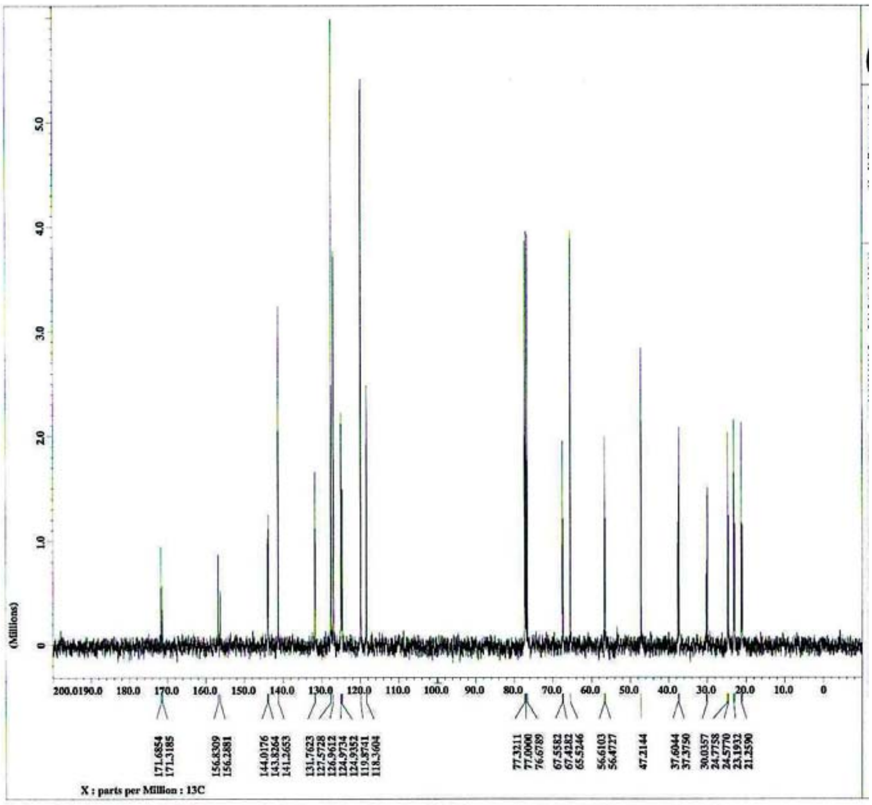

H-D-(*N*-Me)Leu-OAllyl (S18)

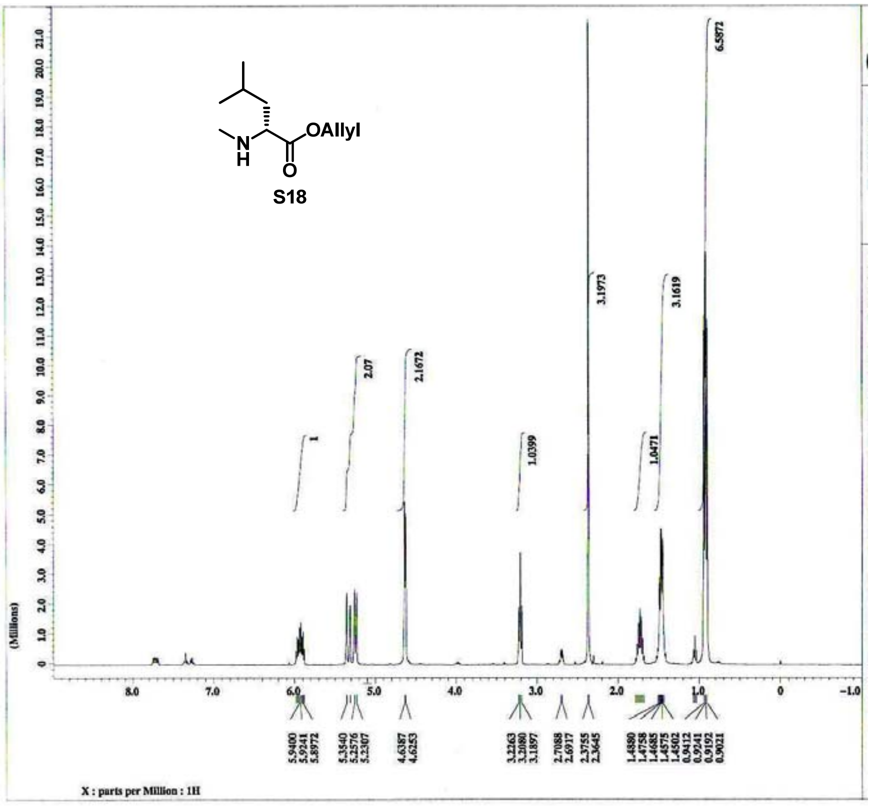

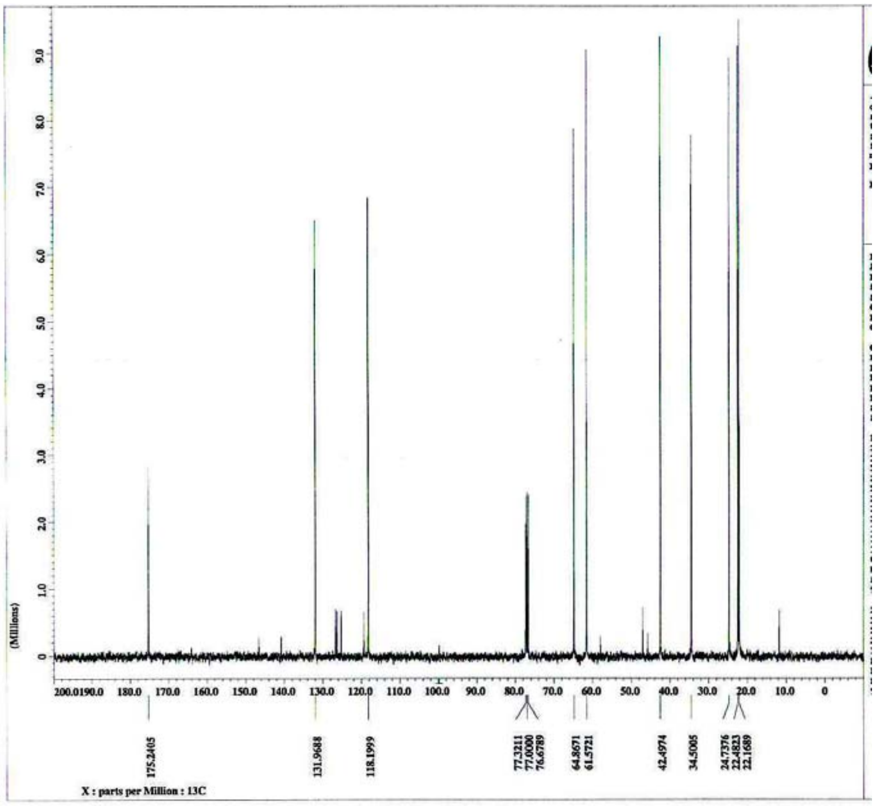

Fmoc-L-Val-D-(*N*-Me)Leu-OAllyl (S15)

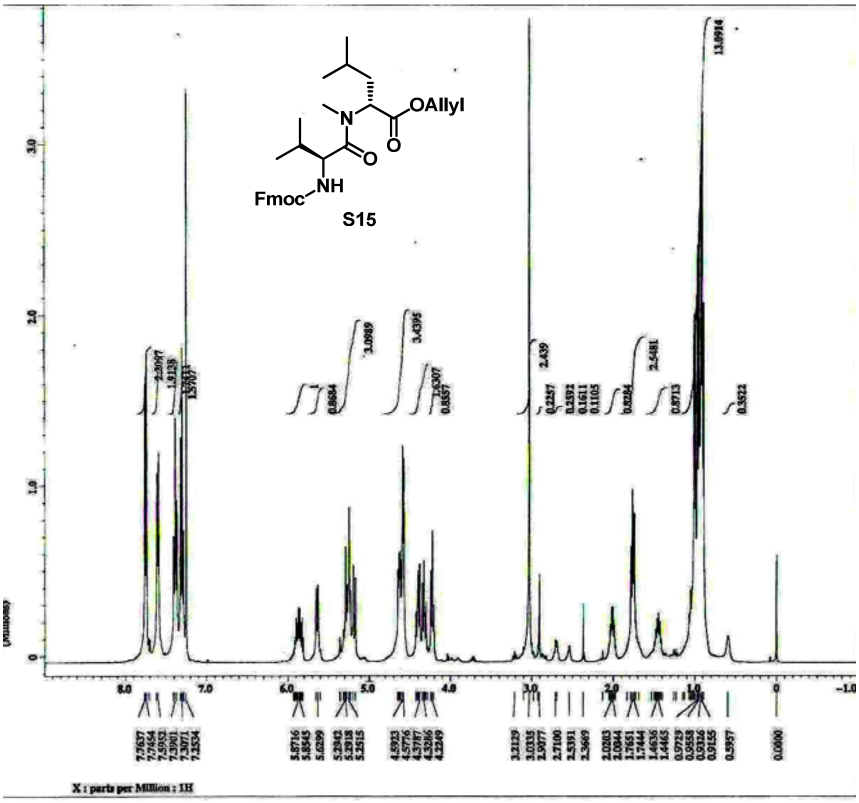

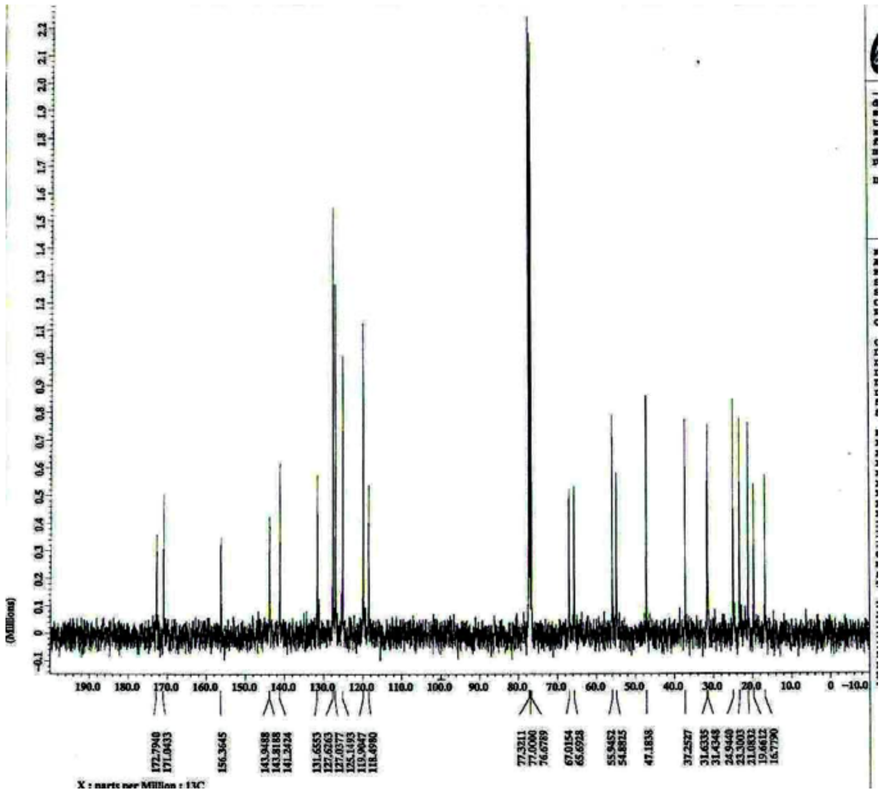

Fmoc-L-Val-D-(*N*-Me)Leu-OH (S16)

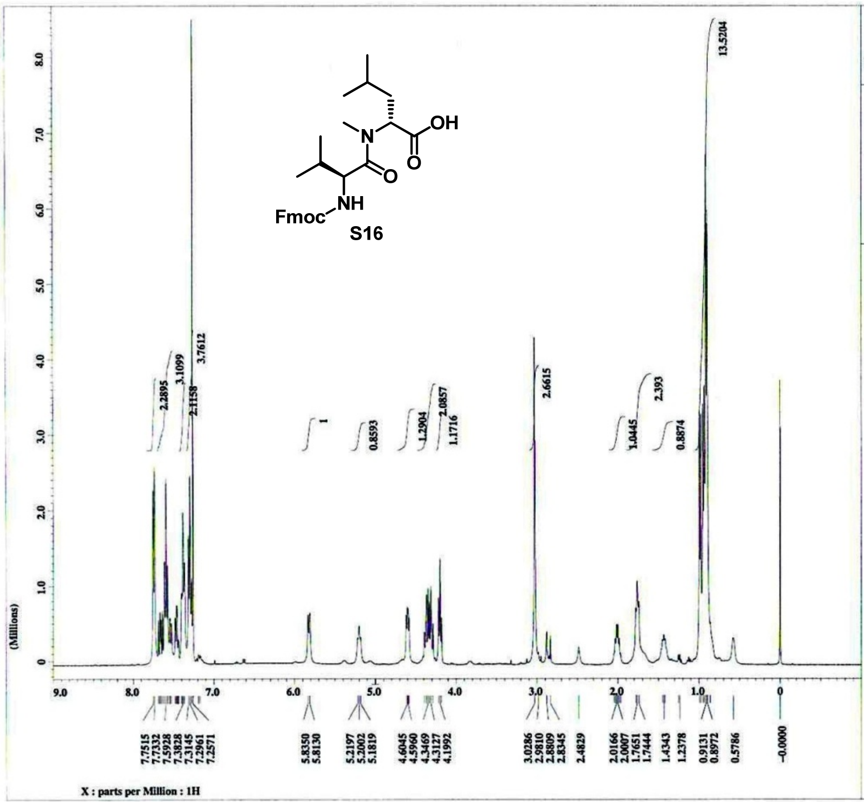

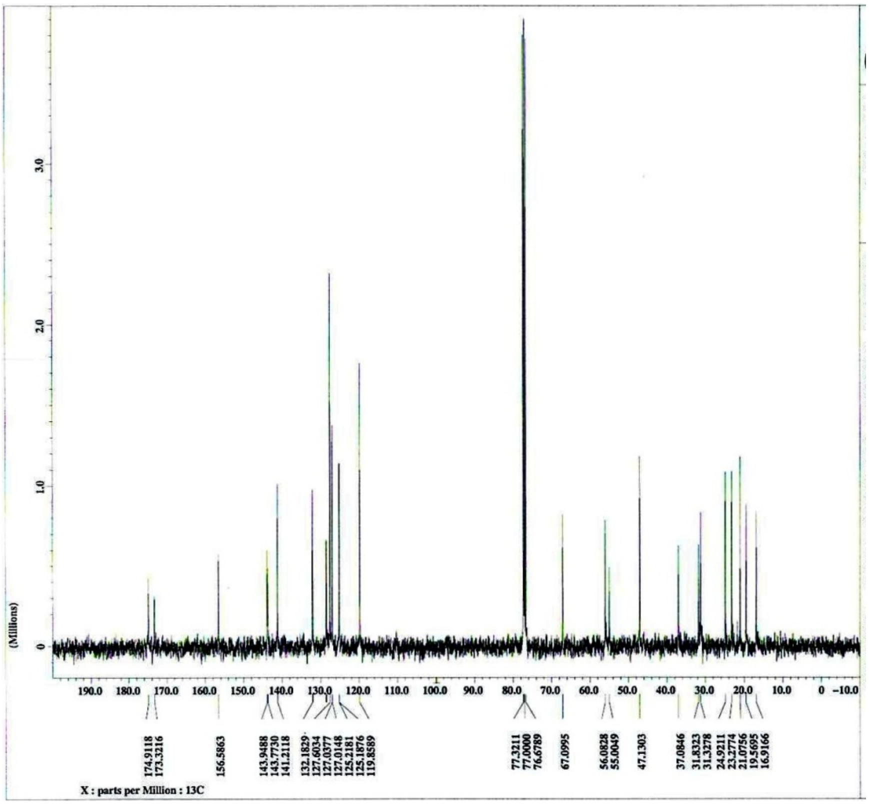

Fmoc-L-(*N*-Me)Leu-Sar-L-Val-OAllyl (S20)

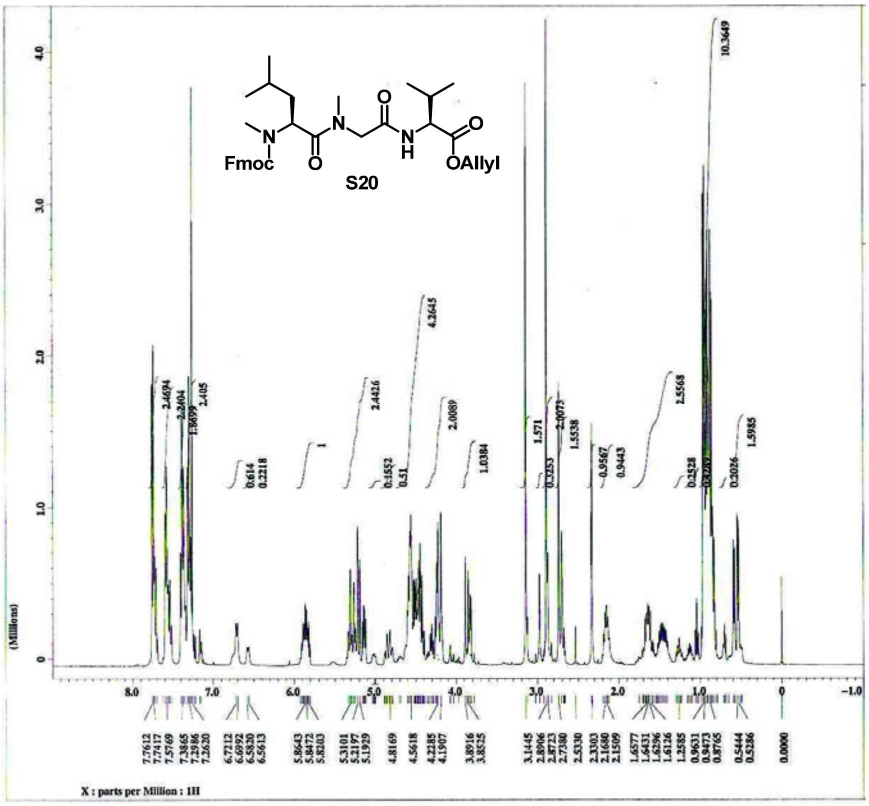

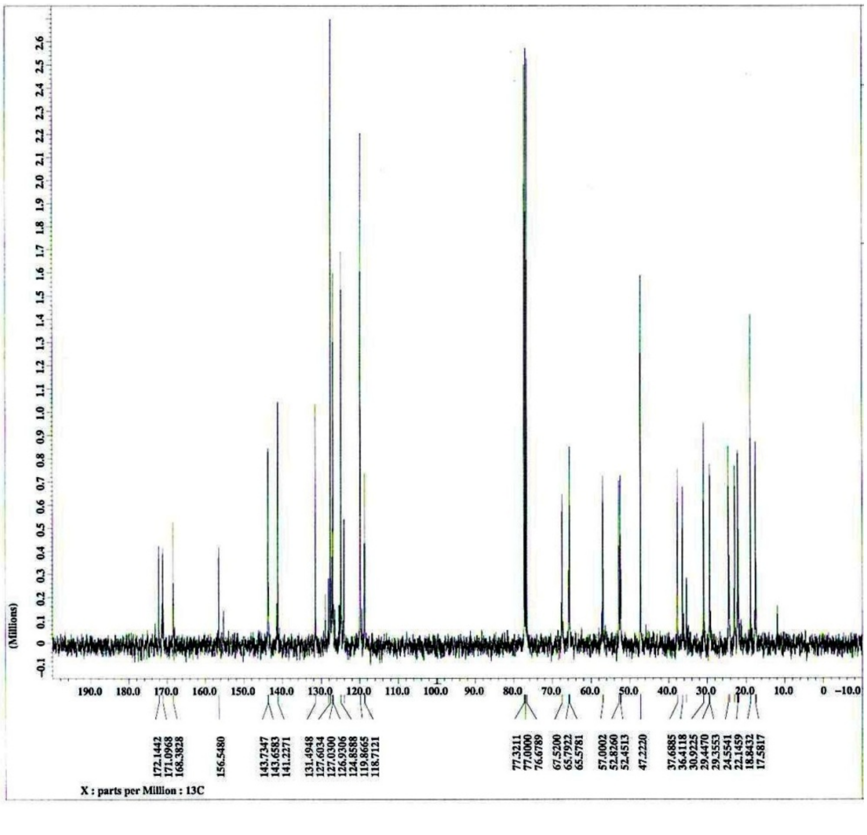

Fmoc-L-Val-L-(*N*-Me)Leu-Sar-L-Val-OAllyl (S21)

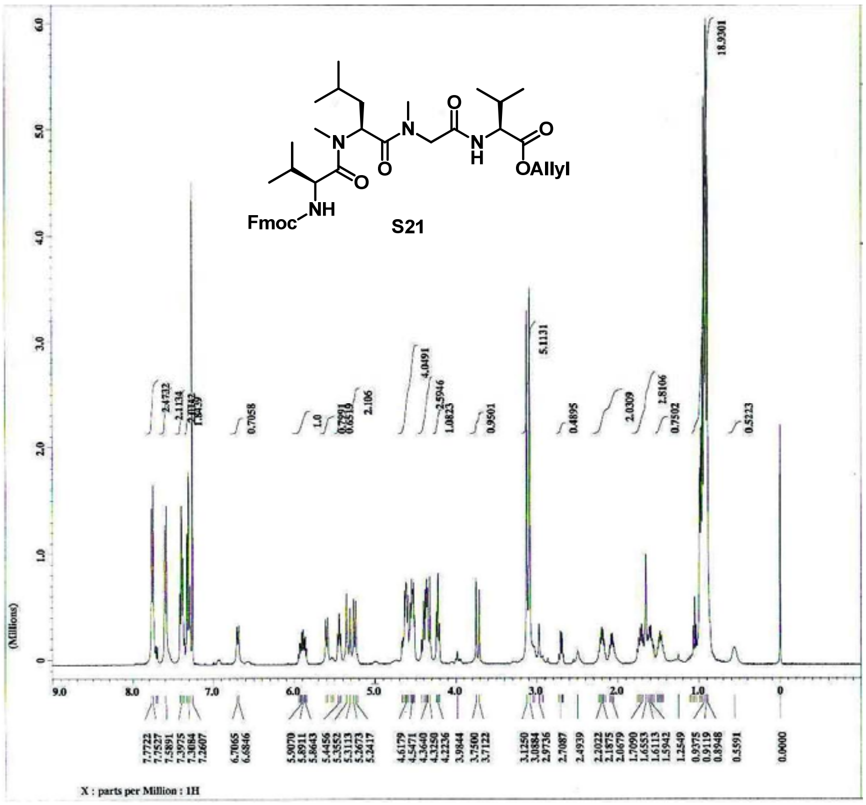

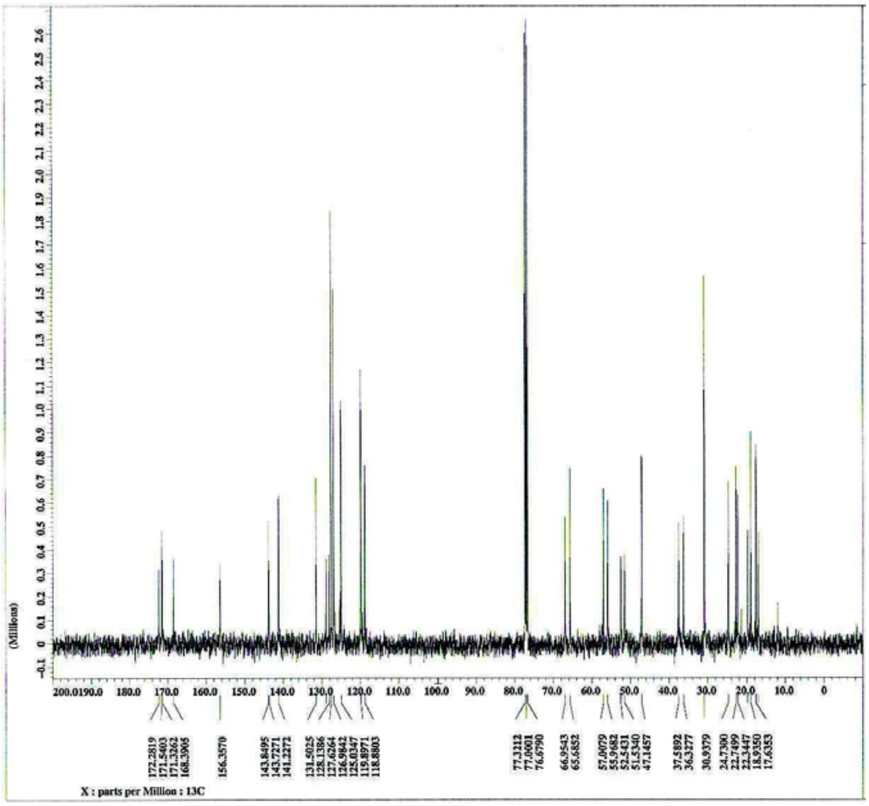

Fmoc-D-Val-D-(*N*-Me)Leu-OAllyl (S23)

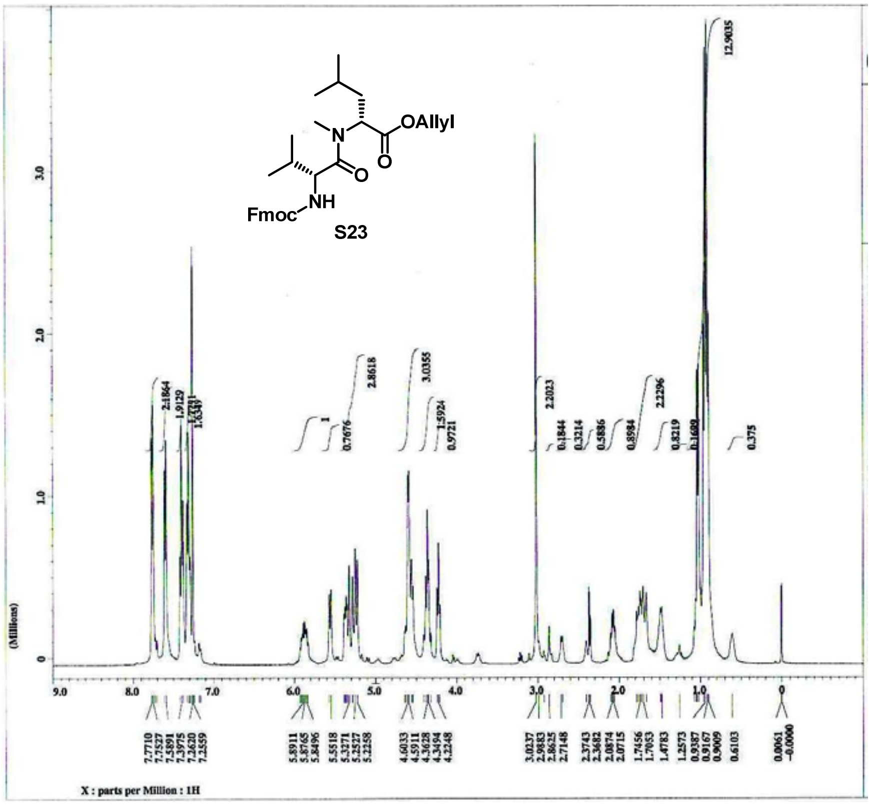

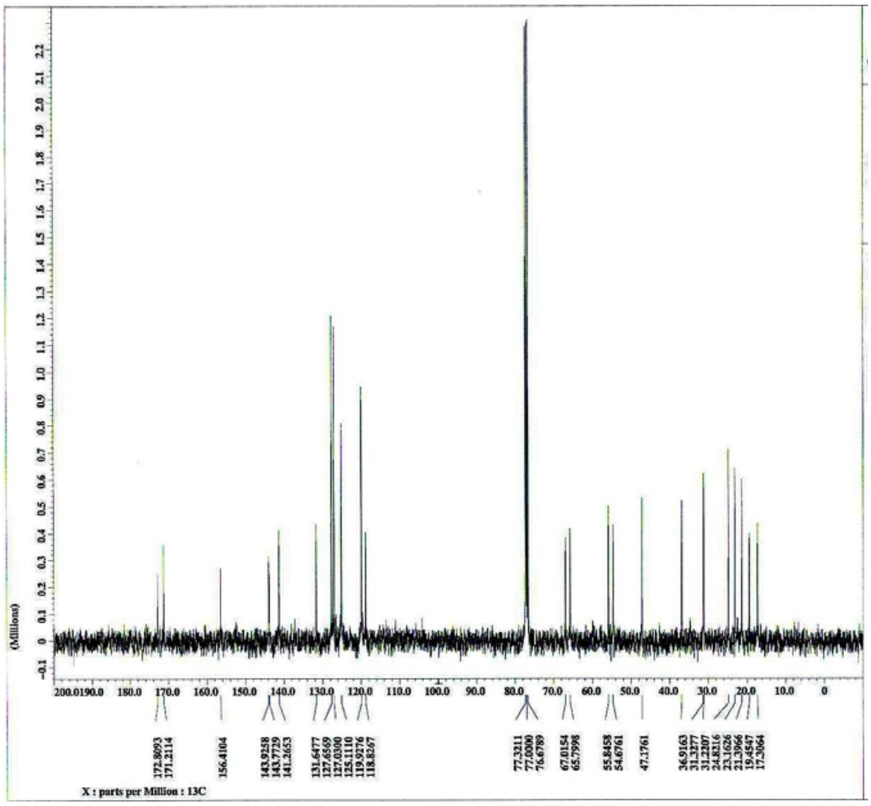

Fmoc-D-Val-D-(*N*-Me)Leu-OH (S24)

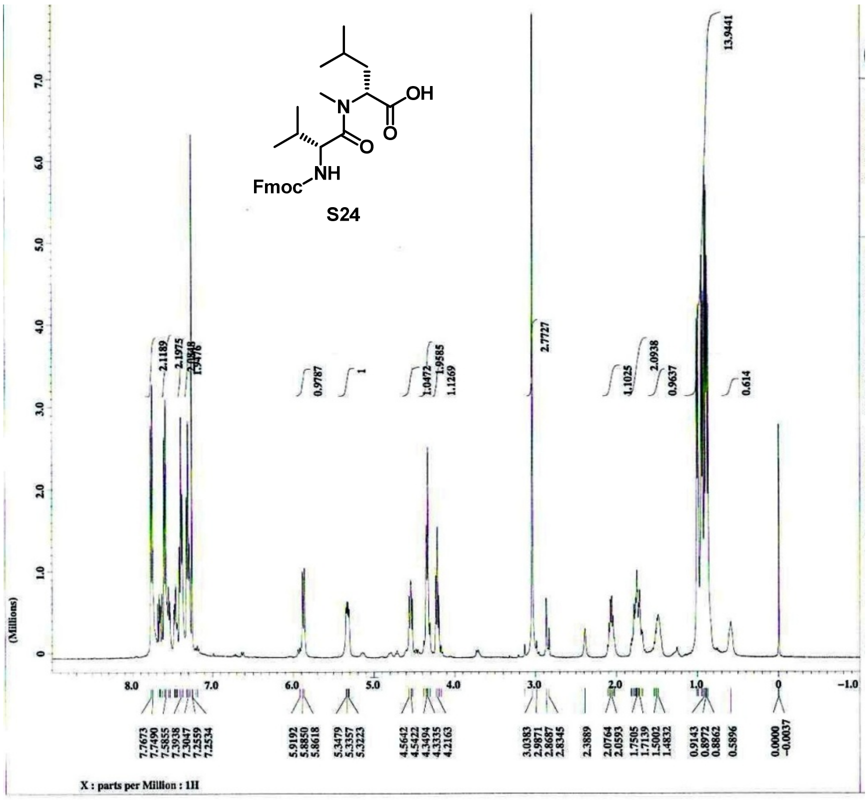

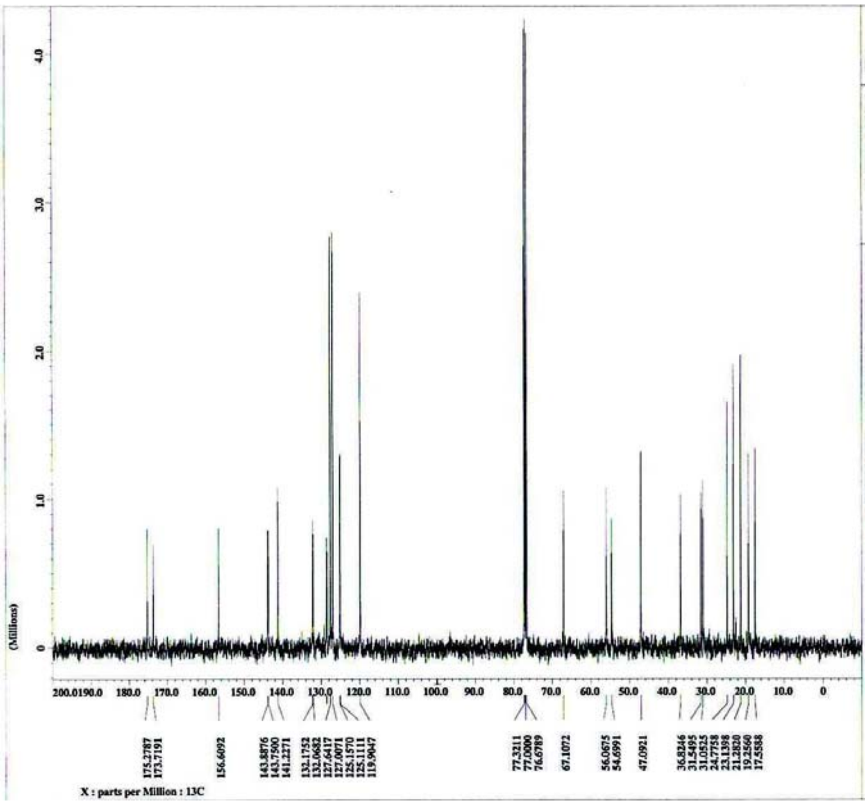

Fmoc-D-Val-D-(*N*-Me)Leu-Sar-L-Val-OAllyl (S25)

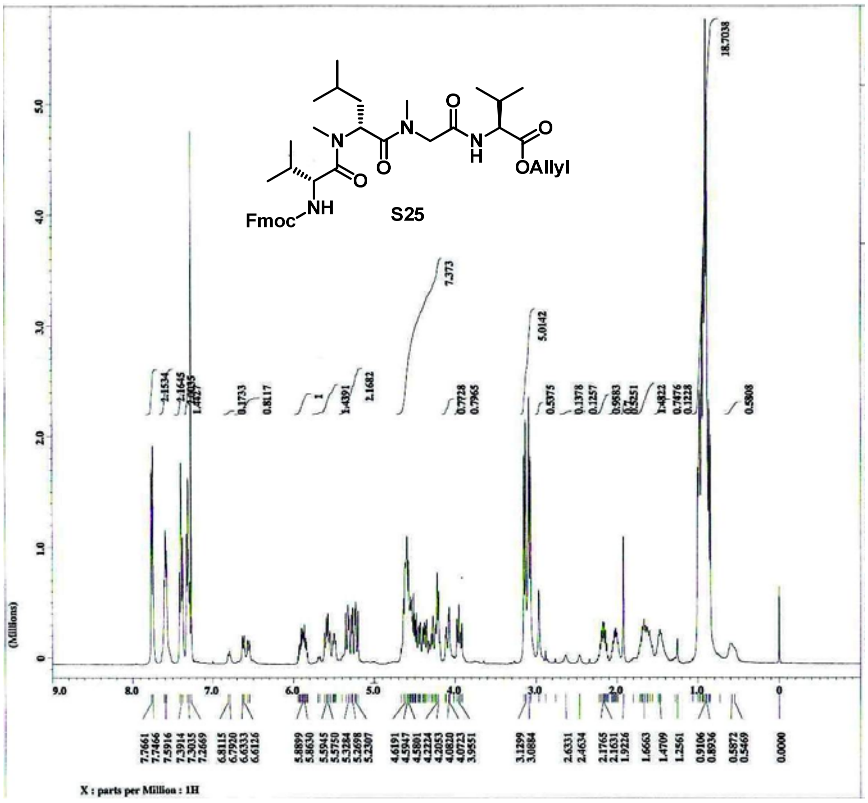

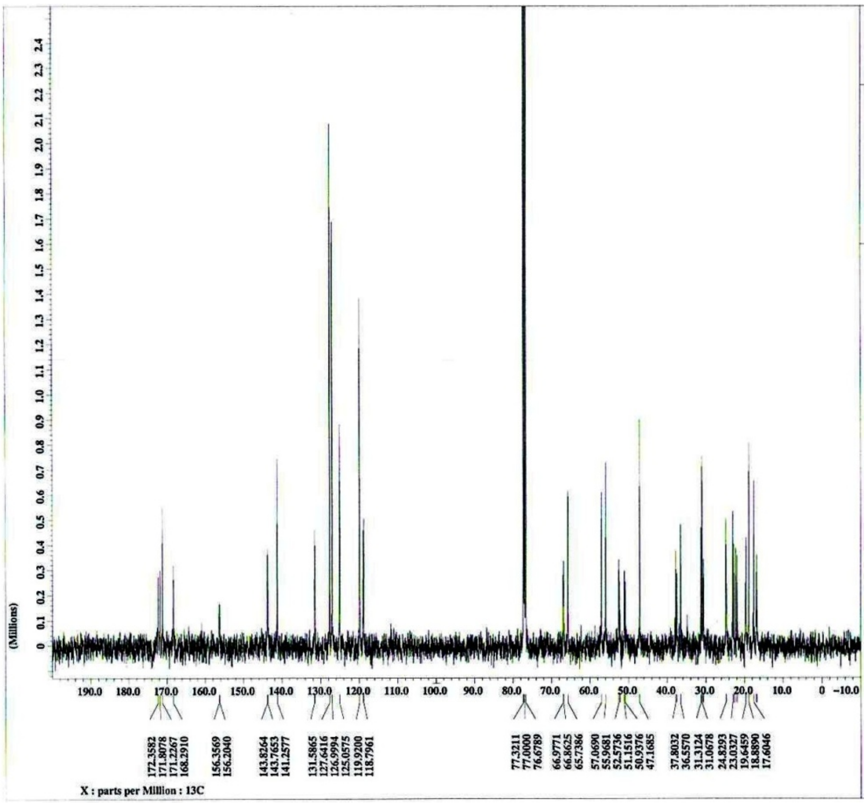

Supplement: Supplementary file 1 [file anie0053-0851-sd1.pdf]
